## [Editor Report · eLife Assessment]

This is an **important** study that reports the mechanism by which Ankle2 (LEM4 in humans) interacts with and recruits PP2A and the ER protein Vap33 to promote BAF dephosphorylation and mediate nuclear membrane reformation, using *Drosophila* as their model. Using Ankle2 mutants, they find that the ER protein Vap33 is key for the normal interphase localisation of Ankle2/LEM4 and also impacts on the function of Ankle2/LEM4 during mitosis. The conclusions on the subcellular localization of Ankle2 are drawn from overexpression of constructs. Overall, the authors use a variety of complementary techniques and provide **convincing** evidence to support the claims and advance our knowledge in the field of mitosis and nuclear envelope biology.

---

## [Referee Report · Reviewer #1 (Public review)]

Summary:

In organisms with an open mitosis, nuclear envelope breakdown at mitotic entry and re-assembly of the nuclear envelope at the end of mitosis are important, highly regulated processes. One key regulator of nuclear envelope re-assembly is the BAF (Barrier-to-Autointegration) protein, which contributes to cross-linking of chromosomes to the nuclear envelope. Crucially, BAF has to be in a dephosphorylated form to carry out this function, and PP2A has been shown to be the phosphatase which dephosphorylates BAF. The Ankle2/LEM4 protein has previously been identified as an important regulator of PP2A in the dephosphorylation of BAF but its precise function is not fully understood, and Li and colleagues set out to investigate the function of Ankle2/LEM4 in both *Drosophila* flies and *Drosophila* cell lines.

Strengths:

The authors use a combination of biochemical and imaging techniques to understand the biology of Ankle2/LEM4. On the whole the experiments are well conducted and the results look convincing. A particular strength of this manuscript is that the authors are able to study both cellular phenotypes and organismal effects of their mutants by studying both *Drosophila* D-mel cells and whole flies.

The work presented in this manuscript significantly enhances our understanding of how Ankle2/LEM4 supports BAF dephosphorylation at the end of mitosis. Particularly interesting is finding that Ankle2/LEM4 appears to be a bona fide PP2A regulatory protein in *Drosophila*, as well as the localisation of Ankle2/LEM4 and how this is influenced by the interaction between Ankle2 and the ER protein Vap33. It would be interesting to see, though, whether these insights are conserved in mammalian cells, e.g. does mammalian Vap33 also interact with LEM4? Is LEM4 also a part of the PP2A holoenzyme complex in mammalian cells?

Weaknesses:

This work is certainly impactful but more discussion and comparison of the *Drosophila* versus mammalian cell system would be helpful. Also, to attract the largest possible readership, the Ankle2 protein should be referred to as Ankle2/LEM4 throughout the paper to make it clear that this is the same molecule.

A schematic model at the end of the final figure would be very useful to summarise the findings.

Comments on revisions:

The authors have carefully revised the manuscripts and have satisfactorily addressed the issues that were raised by the reviewers.

---

## [Referee Report · Reviewer #2 (Public review)]

The authors first identify Ankle2 as a regulatory subunit and direct interactor of PP2A, showing they interact both in vitro and in vivo to promote BAF dephosphorylation. The Ankyrin domain of Ankle2 is important for the interaction with PP2A. They then show Ankle2 also interacts with the ER protein Vap33 through FFAT motifs and they particularly co-localize during mitosis. The recruitment of Ankle2 to Vap33 is essential to ER and nuclear envelop membrane in telophase while earlier in mitosis, it relies on the C terminus but not the FFAT motifs for recruitments to the nuclear membrane and spindle envelop in early mitosis. The molecular determinants and receptors are currently not known. The authors check the function of the PP2A recruitment to Ankle2/Vap33 in the context of embryos and show this recruitment pathway is functionally important. While the Ankle2/Vap33 interaction is dispensable in adult flies -looking at wing development, the PP2A/Ankle2 interaction is essential for correct wing and fly development. Overall, this is a very complete paper that reveals the molecular mechanism of PP2A recruitment to Ankle2 and studies both the cellular and the physiological effect of this interaction in the context of fly development.

The paper is well-written and the narrative is well developed. The figures are of high quality, well-controlled, clearly labelled and easy to understand. They support the claims made by the authors.

Comments on revisions:

There are still issues with the statistics. On graphs where multiple conditions are shown, you cannot perform a T-test. You have to use other tests such as ANOVA if the data is normal, and other tests such as KS test if the data is not normally distributed.

---

## [Referee Report · Reviewer #3 (Public review)]

The authors were interested in how Ankle2 regulates nuclear envelope reformation after cell division. They show that Ankle2 can bind in a PP2A complex without other known regulatory subunits of PP2A. The authors also identity a novel interaction with ER protein Vap33 that could be important for localization. This manuscript is a useful finding linking Ankle2 function during nuclear envelope reformation to the PP2A complex. The authors present solid data showing that Ankle2 can form a complex with PP2A-29B and Mts and generate a phosphoproteomic resource that is fundamentally important to understand Ankle2 biology. The caveat should be remembered that most experiments, including subcellular localization, are based on overexpression data. Keeping this in mind, the manuscript is a valuable resource.

---

## [Author Response]

The following is the authors’ response to the original reviews.

**Public Reviews:**

**Reviewer #1 (Public review):**
Summary:In organisms with open mitosis, nuclear envelope breakdown at mitotic entry and re‐assembly of the nuclear envelope at the end of mitosis are important, highly regulated processes. One key regulator of nuclear envelope re‐assembly is the BAF (Barrier‐to‐Autointegration) protein, which contributes to cross‐linking of chromosomes to the nuclear envelope. Crucially, BAF has to be in a dephosphorylated form to carry out this function, and PP2A has been shown to be the phosphatase that dephosphorylates BAF. The Ankle2/LEM4 protein has previously been identified as an important regulator of PP2A in the dephosphorylation of BAF but its precise function is not fully understood, and Li and colleagues set out to investigate the function of Ankle2/LEM4 in both *Drosophila* flies and *Drosophila* cell lines.Strengths:The authors use a combination of biochemical and imaging techniques to understand the biology of Ankle2/LEM4. On the whole, the experiments are well conducted and the results look convincing. A particular strength of this manuscript is that the authors are able to study both cellular phenotypes and organismal effects of their mutants by studying both *Drosophila* D‐mel cells and whole flies.The work presented in this manuscript significantly enhances our understanding of how Ankle2/LEM4 supports BAF dephosphorylation at the end of mitosis. Particularly interesting is the finding that Ankle2/LEM4 appears to be a bona fide PP2A regulatory protein in *Drosophila*, as well as the localisation of Ankle2/LEM4 and how this is influenced by the interaction between Ankle2 and the ER protein Vap33. It would be interesting to see, though, whether these insights are conserved in mammalian cells, e.g. does mammalian Vap33 also interact with LEM4? Is LEM4 also a part of the PP2A holoenzyme complex in mammalian cells?We feel that conducting experiments to test the level of conservation of our findings in mammalian cells is outside the scope of our study, and we will leave it for other labs to investigate.Weaknesses:This work is certainly impactful but more discussion and comparison of the *Drosophila* versus mammalian cell system would be helpful. Also, to attract the largest possible readership, the Ankle2 protein should be referred to as Ankle2/LEM4 throughout the paper to make it clear that this is the same molecule.We have reinforced our presentation and discussion of similarities and differences between Ankle2 from *Drosophila* vs humans where relevant throughout the Introduction and Discussion sections. Additionally, we have added the mention that Ankle2 is also called LEM4 in humans in the Abstract and Introduction. However, when referring to *Drosophila* Ankle2, we do not use LEM4 because it is not listed as an alternate name for this gene/protein in FlyBase.A schematic model at the end of the final figure would be very useful to summarise the findings.

We have already provided a schematic model in Figure S3, where we think it is better placed.

**Reviewer #2 (Public review):**
The authors first identify Ankle2 as a regulatory subunit and direct interactor of PP2A, showing they interact both in vitro and in vivo to promote BAF dephosphorylation. The Ankyrin domain of Ankle2 is important for the interaction with PP2A. They then show Ankle2 also interacts with the ER protein Vap33 through FFAT motifs and they particularly co‐localize during mitosis. The recruitment of Ankle2 to Vap33 is essential to ER and nuclear envelop membrane in telophase while earlier in mitosis, it relies on the C terminus but not the FFAT motifs for recruitments to the nuclear membrane and spindle envelop in early mitosis. The molecular determinants and receptors are currently not known. The authors check the function of the PP2A recruitment to Ankle2/Vap33 in the context of embryos and show this recruitment pathway is functionally important. While the Ankle2/Vap33 interaction is dispensable in adult flies ‐looking at wing development, the PP2A/Ankle2 interaction is essential for correct wing and fly development. Overall, this is a very complete paper that reveals the molecular mechanism of PP2A recruitment to Ankle2 and studies both the cellular and the physiological effect of this interaction in the context of fly development.Strengths:The paper is well written and the narrative is well‐developed. The figures are of high quality, wellcontrolled, clearly labelled, and easy to understand. They support the claims made by the authors.Weaknesses:The study would benefit from being discussed in the context of what is already known on Ankle2 biology in *C. elegans* and human cells. It is important to highlight the structures shown in the paper are alphafold models, rather than validated structures.

We have enhanced our presentation of what is known about LEM‐4L/Ankle2 in *C. elegans* and humans in the Introduction, and further developed comparisons of our findings regarding *Drosophila* Ankle2 with these orthologs in the Results and Discussion sections. We have also specified in all sections and figure legends that the structures shown are AlphaFold3 models.

**Reviewer #3 (Public review):**
Summary:The authors were interested in how Ankle2 regulates nuclear envelope reformation after cell division. Other published manuscripts, including those from the authors, show without a doubt that Ankle2 plays a role in this critical process. However, the mechanism by which Ankle2 functions was unclear. Previous work using worms and humans (Asencio et al., 2012) established that human ANKLE2 could bind endogenous PP2A subunits. The binding was direct and was mediated through a region before and including the first ankyrin repeat in human ANKLE2. In addition to its interaction with PP2A, Asencio et al., 2012 also show that ANKLE2 regulates VRK1 kinase activity. Together PP2A and VRK1 regulate BAF phosphorylation for proper nuclear envelope reformation. Here, the authors provide more evidence for interaction with PP2A by also mapping the domain of interaction to the ankyrin repeat in *Drosophila*. In addition, the ankyrin repeat is essential for nuclear envelope reformation after division. They show that Ankle2 can bind in a PP2A complex without other known regulatory subunits of PP2A. The authors also identify a novel interaction with ER protein Vap33, but functional relevance for this interaction in nuclear envelope reformation is not provided in the manuscript, which the authors explicitly state. This manuscript does not comment on the activity of Ballchen/VRK1 in relation to Ankle2 loss and BAF phosphorylation or nuclear envelope reformation, even though links were previously shown by multiple studies (Asencio et al., Link et al., Apridita Sebastian et al.,). Nuclear envelope defects were rescued by the reduction of VRK1 in two of these manuscripts. It is possible that BAF phosphorylation phenotypes can be contributed by both PP2A inactivity and VRK1 overactivity due to the loss of Ankle2.Strengths:This manuscript is a useful finding linking Ankle2 function during nuclear envelope reformation to the PP2A complex. The authors present solid data showing that Ankle2 can form a complex with PP2A‐29B and Mts and generate a phosphoproteomic resource that is fundamentally important to understanding Ankle2 biology.Weaknesses:However, the main findings/conclusions about subcellular localization might be incomplete since they are drawn from overexpression experiments. In addition, throughout the text, some conclusions are overstated or are not supported by data.

It is true that all experiments studying subcellular localization were done with tagged proteins overexpressed in flies and cell culture. Nevertheless, we show that Ankle2‐GFP is functional since it rescues phenotypes resulting from the loss of endogenous Ankle2 in both flies and cultured cells. The antibodies we generated against Ankle2 were unable to reliably detect the endogenous protein by immunofluorescence. We have now stated this caveat in our manuscript. Regarding the validity of our conclusions in relation to our data, we address each point raised by the reviewer under the Recommendations for the authors. In some cases, we have adjusted our conclusions and in other cases, we have provided additional clarification or justification.

**Recommendations for the authors:**

**Reviewer #1 (Recommendations for the authors):**
There are a few experimental issues that should be addressed, specific comments are listed below:(1) Figure 1F: In this experiment, the authors immunoprecipitate GFP‐PP2A‐29B or PP2A‐B29BGFP and Western blot for Ankle2 and Mts to demonstrate that both are co‐immunoprecipitated. To demonstrate that these interactions are specific, the authors should also blot for a protein that is expected to definitely NOT co‐immunoprecipitate with PP2A‐B29; e.g. tubulin.

Our conclusion that GFP‐PP2A‐29B and PP2A‐29B‐GFP specifically interact with Ankle2 and Mts is also based on mass spectrometry analysis of the purification products from embryos and cells in culture, comparing with products of purification of GFP alone (Fig 1E‐F, S1C‐D and Tables S2, S3). The lists of identified proteins reveal that most proteins (including tubulins) are not enriched with GFP‐PP2A‐29B or PP2A‐29B‐GFP like Ankle2 and Mts are.

(2) Figure 2A: The colour coding of the dots is not explained in the figure legend.

We have now added the explanation.

(3) Figure 2B: The competition experiment is a good idea. Do the authors get the same results when they conduct the experiment the other way round, i.e. keep the concentration of Tws the same but increase the concentration of Ankle2?

We have tried this reverse experiment but saw little effect. The failure to observe displacement of Tws by Ankle2 in this context could be due to a higher affinity of Tws than Ankle2 in the PP2A complex, or to lower expression levels achieved for Ankle2 (a larger protein) relative to Tws.

(4) Figure 5D: The hyperphosphorylation of BAF is very difficult to see, and it is impossible to tell whether the hyperphosphorylation has been rescued or not by the different Ankle2 constructs. Can the phosphorylated and the hyperphosphorylated bands be separated better? This panel needs significant improvements to support the claims in the text.

In our opinion, the hyperphosphorylated (upper band) and unphosphorylated (lower band) forms of BAF are well resolved and readily distinguishable. The fainter band in the middle could correspond to a partially phosphorylated form of BAF but we do not venture to speculate on its precise identity nor do we need it to draw our conclusions. The important information from this blot is that the level of unphosphorylated BAF after Ankle2 RNAi increases when Ankle2WT‐GFP and Ankle2Fm+FL1‐GFP are expressed but not when Flag‐GFP or Ankle2ΔANK‐GFP are expressed. In these experiments, the rescue of unphosphorylated BAF is incomplete because not all cells express the GFP‐tagged protein in our non‐clonal stable cell lines.

**Reviewer #2 (Recommendations for the authors):**
(1) The alphafold models need to be labelled as such better on the figures, to distinguish them from X‐ray crystallography structures. Alphafold will always propose a solution but it is not necessarily correct.

We have added the note “MODEL” directly in Figures 2C, 2D, 4F and S3B, in addition to the information already provided in the text and figure legends specifying that these are models generated by AlphaFold3.

(2) Figure 4 F. Annotate the Ankle2 FL1 peptide.

We have indicated the amino acid residues in the figure.

(3) Problems with the statistical tests. T‐tests cannot be used for comparing multiple groups, as this favors error propagation.

All of our t‐tests compare only two groups at a time, as indicated. In this regard, our labeling in Fig 5C may have been misleading. We have now changed it.

(4) Close‐ups of ring canal in Figure S2. In Figure S2, there seem to be lots of GFP‐Ankle2 vesicles in the cytoplasm of the oocyte.

We agree that the image showing Ankle2‐GFP alone in the RNAi Vap33 condition suggested a cytoplasmic granular localization of unknown nature. However, upon examination, we realized that this image did not correspond to the same z‐step as the matching merged image (which also included DNA staining). We have now replaced the image with the correct one.

**Reviewer #3 (Recommendations for the authors):**
Be more accurate about what conclusions can be made from reported data, particularly from overexpression and deletion studies.(1) The domain analysis for physical interaction is quite thorough. However, localization information is taken from overexpressed constructs. While these data show what could happen, the authors are not using endogenous levels of Ankle2 in cells or tissues that are known to require Ankle2. As a result, it is difficult to determine whether localization results are biologically meaningful.

We have added the following text at the end of the third Results section:

“We were unable to examine the localization of endogenous Ankle2 because the antibodies that we generated gave inconclusive results in immunofluorescence. For the remainder of our study, we relied on the overexpression of Ankle2‐GFP, which may not perfectly reflect the localization and function of endogenous Ankle2. However, Ankle2‐GFP is functional as it can rescue phenotypes observed when endogenous Ankle2 is depleted (see below).”

(2) The data showing that Ankle2 is a regulator unit of the PP2A complex also relies on in vitro binding assays in an over‐expression context. Data certainly show Ankle2 can bind proteins in the PP2A complex when overexpressed. However, the authors could not isolate enough of the complex from the animal to test function, so Ankle2 acting as a regulatory subunit isn't functionally shown. There are other possibilities, such as Ankle2 acts as a scaffold for complex assembly.

The competition experiments shown in Fig 2 are based on complexes assembling in cells and are not in vitro binding assays. We show 4 lines of evidence supporting the idea that Ankle2 functions as a regulatory subunit of PP2A: (1) Ankle2 interacts with the structural (PP2A‐29B) and catalytic (Mts) subunits of PP2A without any known regulatory subunit of PP2A. (2) Depletion of Ankle2 leads to the hyperphosphorylation of the known PP2A substrate BAF. (3) The PP2A regulatory subunit Tws/B55 competes with Ankle2 for formation of a complex with PP2A. (4) AlphaFold3 predicts that Ankle2 engages in a complex with PP2A at a position similar to that of known regulatory subunits of PP2A including Tws/B55, and consistent with their mutually exclusive presence in PP2A complexes. If Ankle2 acted as a scaffold for the formation of a PP2A complex containing other regulatory subunits, we would expect to detect Ankle2 and another regulatory subunit in the same complex.

(3) Throughout the text, some conclusions are overstated or are not supported by data. Examples are below:a. Page 1: "we show for the first time that Ankle2 is a regulatory subunit of PP2A" The authors show binding and changes in BAF phosphorylation levels, but changes in PP2A activity with modulation of Ankle2 weren't shown.

We have replaced this phrase with this one:

“…we provide several lines of evidence that suggest that Ankle2 is a regulatory subunit of PP2A…”

b. Page 3: "The requirement for Ankle2 in the development of the central nervous system was initially discovered through its targeting by the microcephaly‐causing Zika virus (Shah et al., 2018)."This is not the first paper showing ANKLE2 plays a role in the development of the CNS. Yamamoto et al., 2014 identified mutants in Ankle2 with defects in CNS development in flies and humans, establishing it as a human microcephaly‐causing gene.

We are sorry for this oversight. We have now cited this important work.

c. Page 6: "Moreover, BAF appears to be the only obligatory substrate of Ankle2‐dependent dephosphorylation for cell proliferation as lowering the dose of the BAF kinase NHK‐1/Ballchen rescues wing development defects caused by the partial depletion of Ankle2 (Li et al., 2024)." It is unclear why the authors conclude this since Ballchen/VRK1 can phosphorylate many things besides BAF.

Although the conclusion cannot be drawn categorically, it seems to be by far the most likely scenario. However, we agree that in principle, other mechanisms could also account for these genetic observations, such as the dephosphorylation of another, still unidentified obligatory substrate of PP2A‐Ankle2 that would also be phosphorylated by NHK‐1/Ballchen. However, we have also shown that expression of an unphosphorylatable mutant form of BAF rescues phenotypes observed upon loss of Ankle2 function (Li et al, 2024). We have changed our sentence as follows:

"Moreover, BAF could be the only obligatory substrate of Ankle2‐dependent dephosphorylation for cell proliferation as lowering the dose of the BAF kinase NHK‐1/Ballchen or expression of an unphosphorylatable mutant form of BAF rescues wing development defects caused by the partial depletion of Ankle2 (Li et al., 2024).”

d. Page 10: "These results suggest that a Vap33‐Ankle2‐PP2A complex can mediate the recruitment of a pool of PP2A at the NE."There is insufficient evidence to indicate that Vap33‐Ankle2‐PP2A exists in a stable state in the cell and that this complex mediates recruitment of PP2A at the NE. The images do not include Vap33, showing no evidence it is present when PP2A is at the NE and the complex could only be detected with overexpression.

We agree with this caveat and recognize the need to be cautious when proposing our model. In this regard, we feel that our wording is reasonable and appropriate, using “suggest” rather than “prove”, “show” or “indicate”.

e. Page 11: These results suggest that the interaction of Ankle2 with PP2A is essential for its function in BAF dephosphorylation and nuclear reassembly." Page 14: "these results indicate that the interaction of Ankle2 with PP2A is essential during embryo". Page 14: "These results indicate that the interaction of Ankle2 with PP2A but not with Vap33 is essential for its function during cell proliferation in imaginal wing disc development."These experiments show that the ankyrin repeat in Ankle2 is necessary for these processes. It does not say PP2A interaction with Ankle2 is necessary because other things could bind the domain.

We have revised the segments of the text mentioned, taking the reviewer’s legitimate concerns into consideration. We have also added the following sentence to the Discussion:

“However, it remains formally possible that the deletion of Ankyrin repeats used to disrupt the Ankle2‐PP2A interaction abrogated another, unknown aspect of Ankle2 function.”

f. Page 12: "Overall, we conclude that in addition to its N‐terminal PP2A‐interacting Ankyrin domain, Ankle2 requires the integrity of its C‐terminal portion for its essential function in nuclear reassembly."No data was shown for differences in nuclear reassembly, only the ability for ANKLE2 truncation mutants to localize to the nuclear envelope. It isn't clear whether the nuclear envelope reformation is normal in Figure S6 which the authors refer to. Lamin staining could help determine and conclude the C‐terminal region is important for nuclear envelope reformation.

Our conclusion is drawn from the results shown in Figures S4 and S5 (described in the same section), where a rescue assay in cells was performed to assess the functionality of different variants of Ankle2‐GFP when endogenous Ankle2 was depleted. In this assay, Lamin and DNA staining were used to examine nuclear reassembly (as in Figure 5). Figure S6 shows the localizations of the different variants of Ankle2‐GFP, but endogenous Ankle2 is not depleted in these cells.

g. Page 13: "We conclude that the ability of Ankle2 to interact with PP2A is required for the timely recruitment of BAF at reassembling nuclei and ensuing NE reassembly."It's possible the Ankyrin domain in ANKLE2 is interacting with proteins other than PP2A to recruit BAF at reassembling nuclei, especially since ANKLE2 is found to regulate VRK1 (Link 2019) which has been found to phosphorylate BAF during the cell cycle (Molitor 2014). Additionally, the images in Figure 6A appear to show fully reassembled nuclear envelopes in all mutants by 180s.

This point relates to point e, raised above by this reviewer. We have re‐written the sentence as follows:

“We conclude that the Ankyrin domain, required for the ability of Ankle2 to interact with PP2A, is necessary for the timely recruitment of BAF at reassembling nuclei and ensuing NE reassembly.”

Please note that in this paragraph, we discuss a delay in RFP‐BAF recruitment, rather than the complete elimination of this recruitment.

h. Page 16: "Our unbiased phosphoproteomic analysis confirmed that BAF dephosphorylation depends on Ankle2, despite the absence of a detectable interaction between *Drosophila* Ankle2 and BAF, which may be due to the lack of a LEM domain in the former (Fishburn et al., 2024). Moreover, while Ankle2 was shown to bind and inhibit the BAF counteracting kinase VRK1 in humans (Asencio et al., 2012), we detected no interaction between Ankle2 and NHK‐1/Ballchen (VRK1 ortholog) in *Drosophila*. This suggests that the loss of Ankle2 causes BAF hyperphosphorylation by preventing PP2A‐dependent dephosphorylation rather than by preventing inhibition of NHK‐1"There could be transient binding between Ankle2 and Ballchen/VRK1/NHK‐1 or activity can be indirect, but that doesn't mean there is not a contribution of BAF phosphorylation by Ballchen/VRK1/NHK‐1. Genetic evidence from three model systems, including *Drosophila*, indicates there is a strong genetic interaction between Ankle2 and Ballchen/VRK1/NHK‐1 that includes rescue of lethality.

We agree and we have re‐written in this way:

“While a putative interaction between Ankle2 and NHK‐1 in *Drosophila* could occur transiently, thereby escaping detection, the simplest interpretation of our results is that the loss of Ankle2 causes BAF hyperphosphorylation by preventing PP2A‐dependent dephosphorylation rather than by preventing inhibition of NHK‐1.”

We do not question the fact that Ballchen/VRK1/NHK‐1 phosphorylates BAF and genetically interacts with Ankle2. The antagonistic relationship between Ballchen/VRK1/NHK‐1 and Ankle2 observed genetically can be explained by the fact that the kinase phosphorylates BAF while PP2AAnkle2 dephosphorylates it, without the need to invoke an additional inhibition of the kinase by Ankle2.