# Supplementary figures and images for: Mechanisms of PP2A-Ankle2 dependent nuclear reassembly after mitosis
Source: eLife. 2025 Feb 18;13:RP104233. doi: 10.7554/eLife.104233 (PMC11835388; doi:10.7554/eLife.104233)

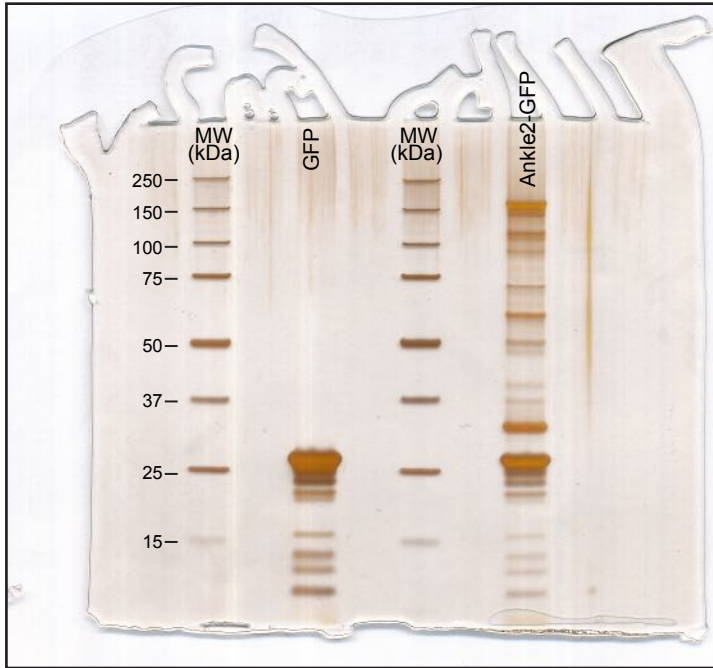

Silver staining

Supplement: Figure 1—source data 3. [file elife-104233-fig1-data3.zip › Figure 1/Figure 1C.pdf]

## WCL

## GFP purification

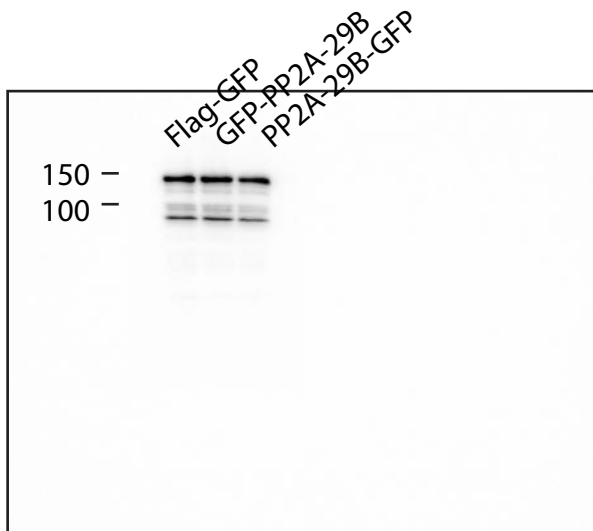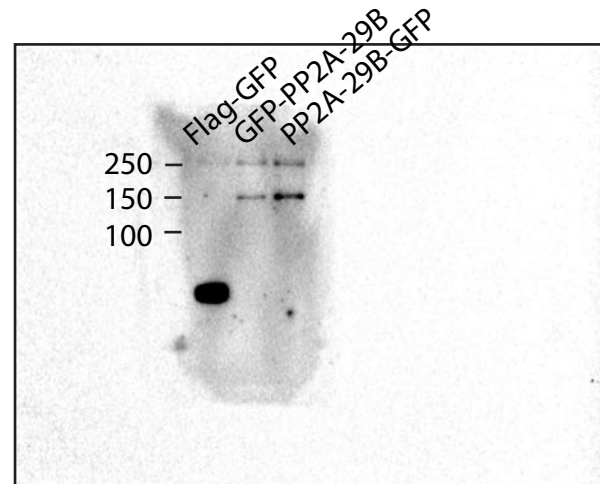

$\alpha$ -Ankle2

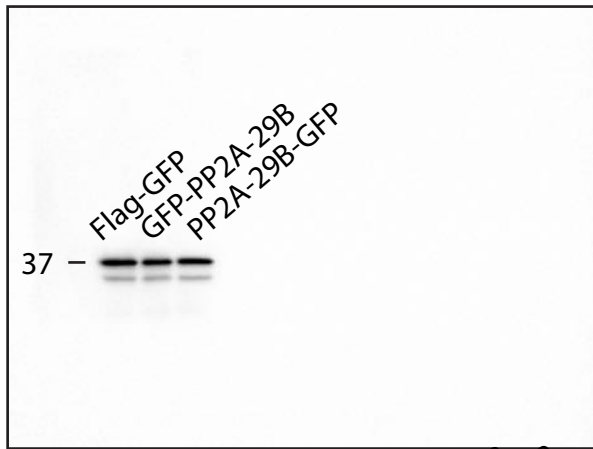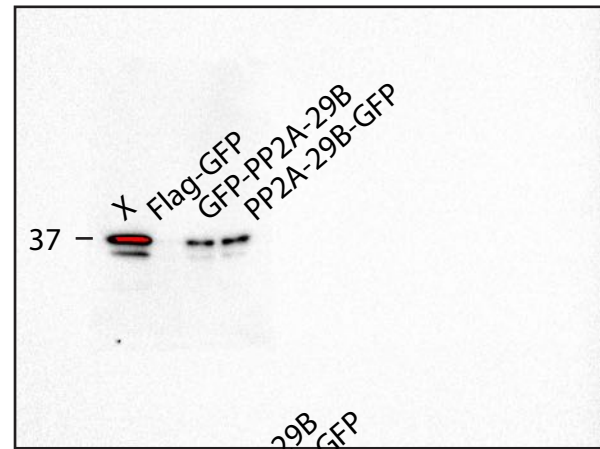

$\alpha$ -Mts

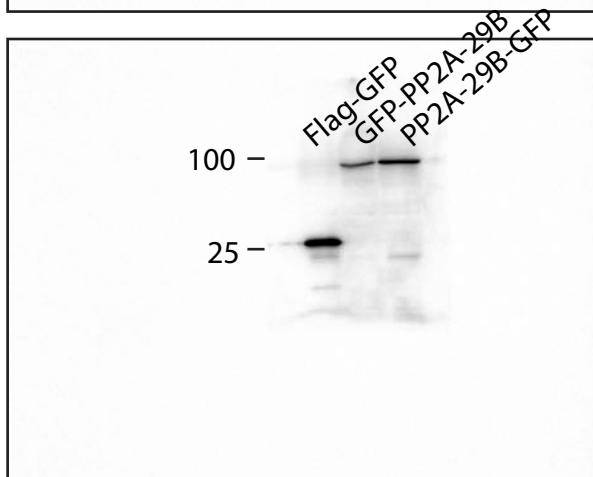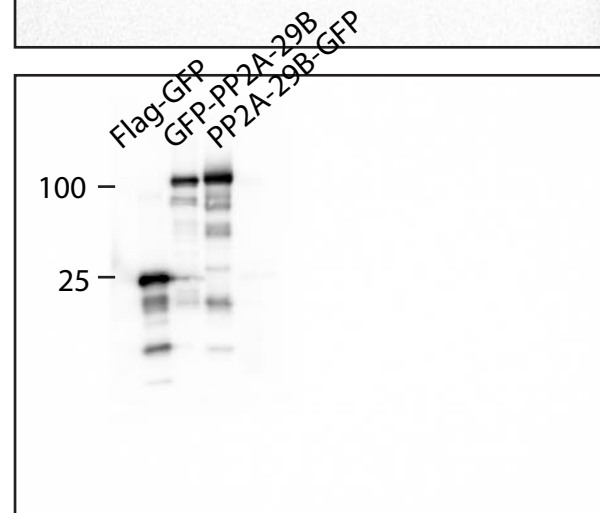

$\alpha$ -GFP

Supplement: Figure 1—source data 3. [file elife-104233-fig1-data3.zip › Figure 1/Figure 1F.pdf]

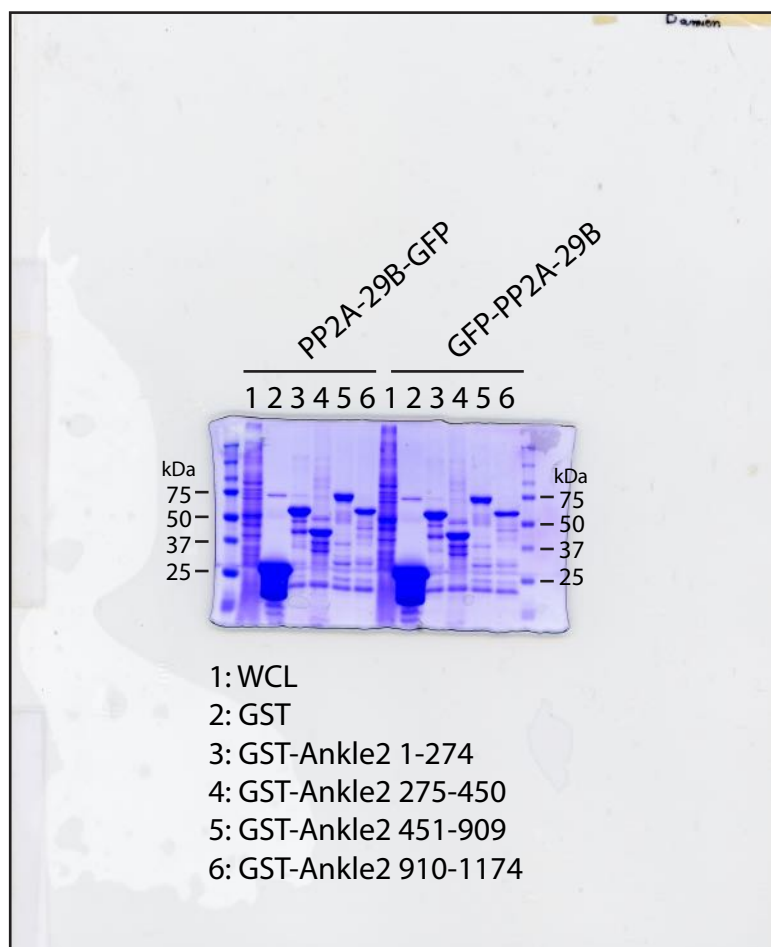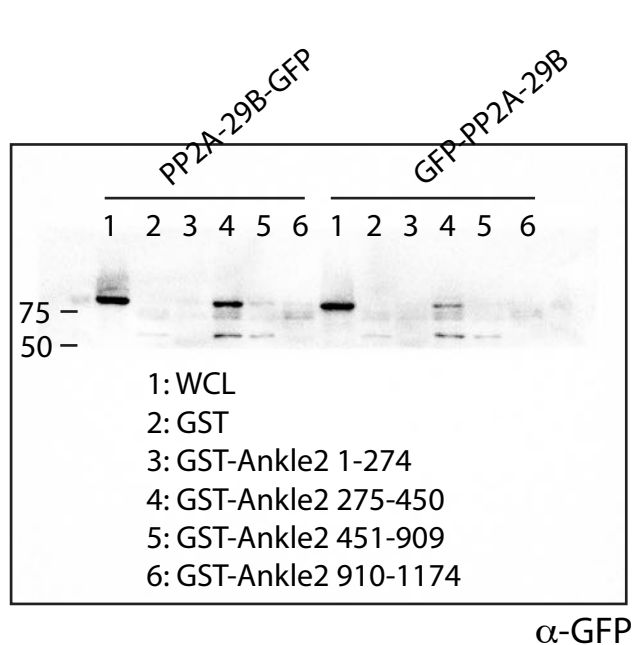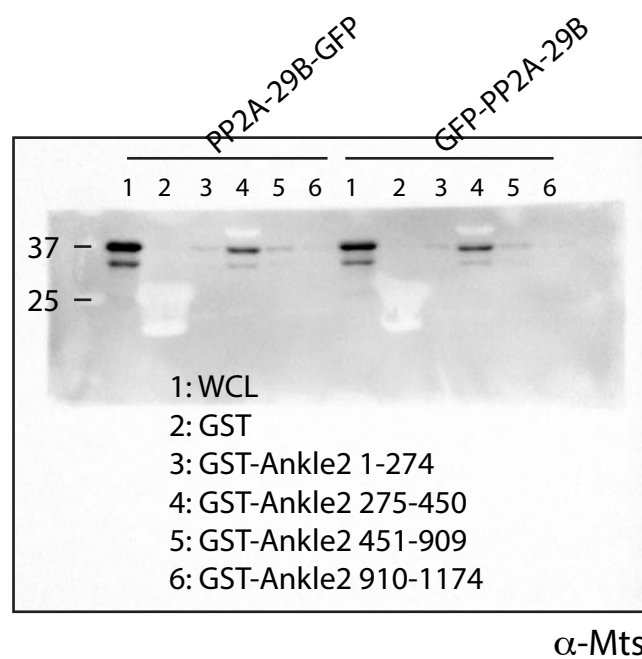

Supplement: Figure 1—source data 3. [file elife-104233-fig1-data3.zip › Figure 1/Figure 1G.pdf]

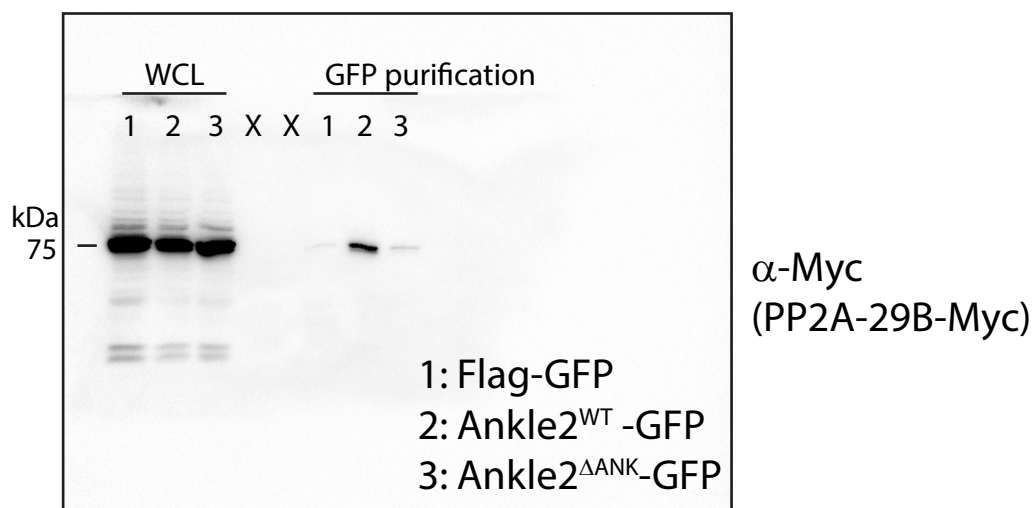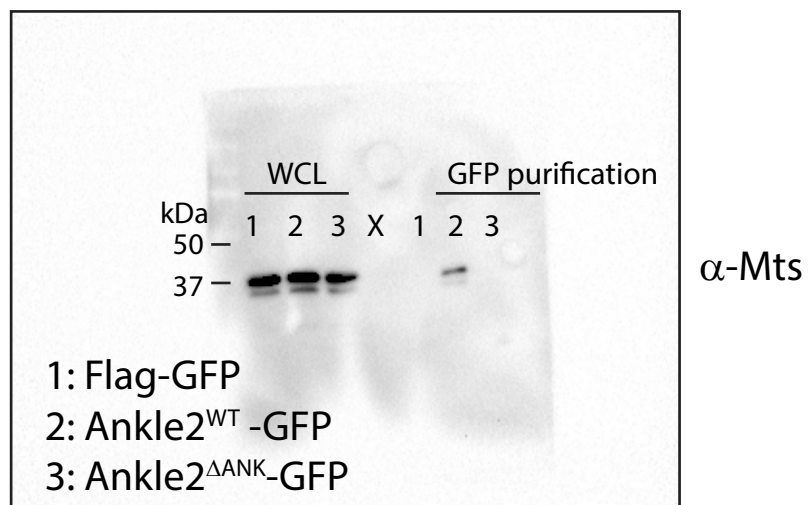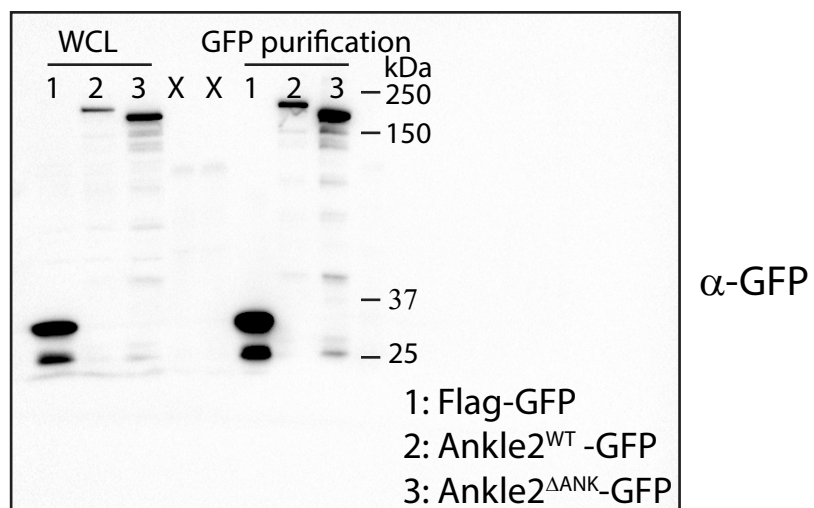

Supplement: Figure 1—source data 3. [file elife-104233-fig1-data3.zip › Figure 1/Figure 1I.pdf]

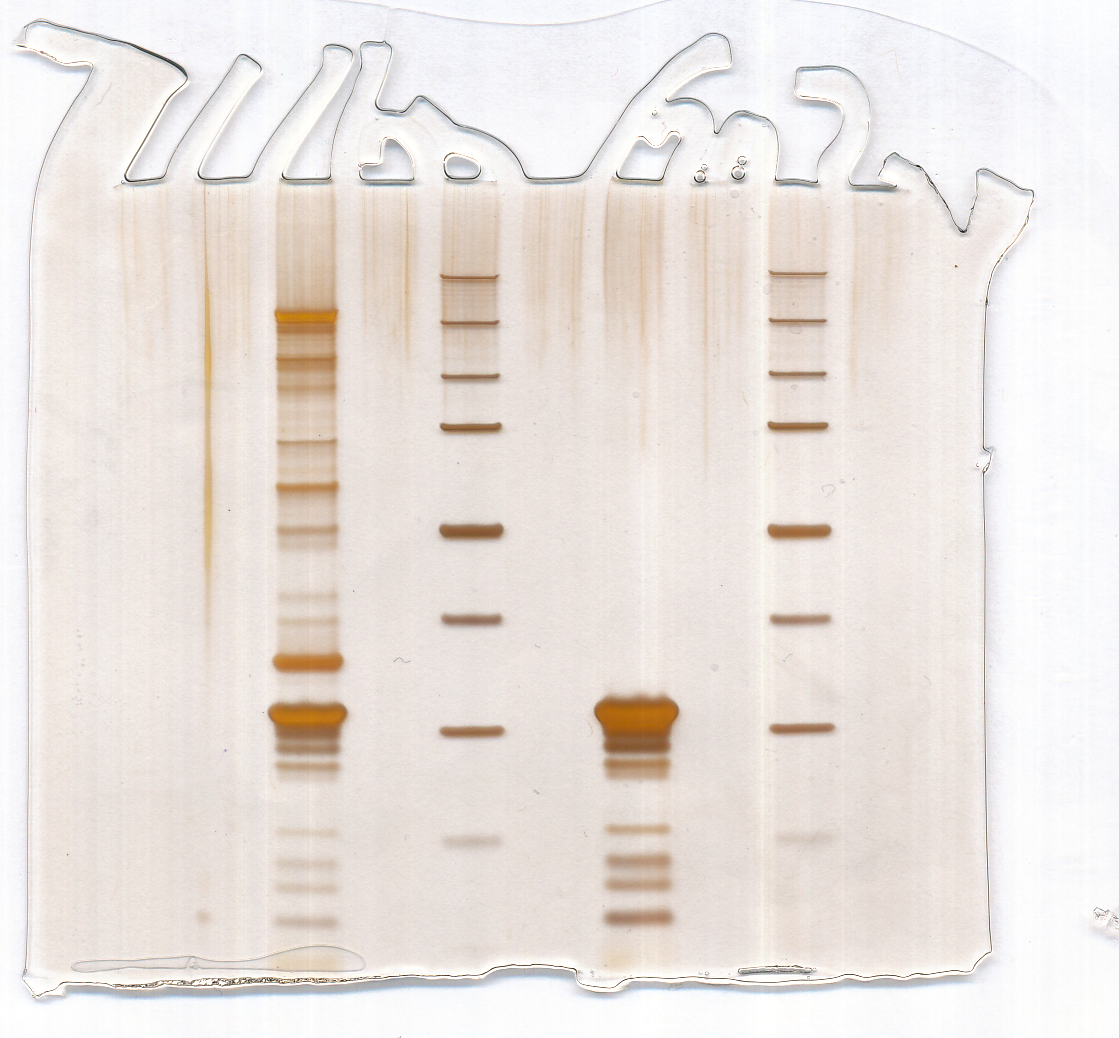

Supplement: Figure 1—source data 4. [file elife-104233-fig1-data4.zip › Figure 1/Fig 1C/Silver staining.tif]

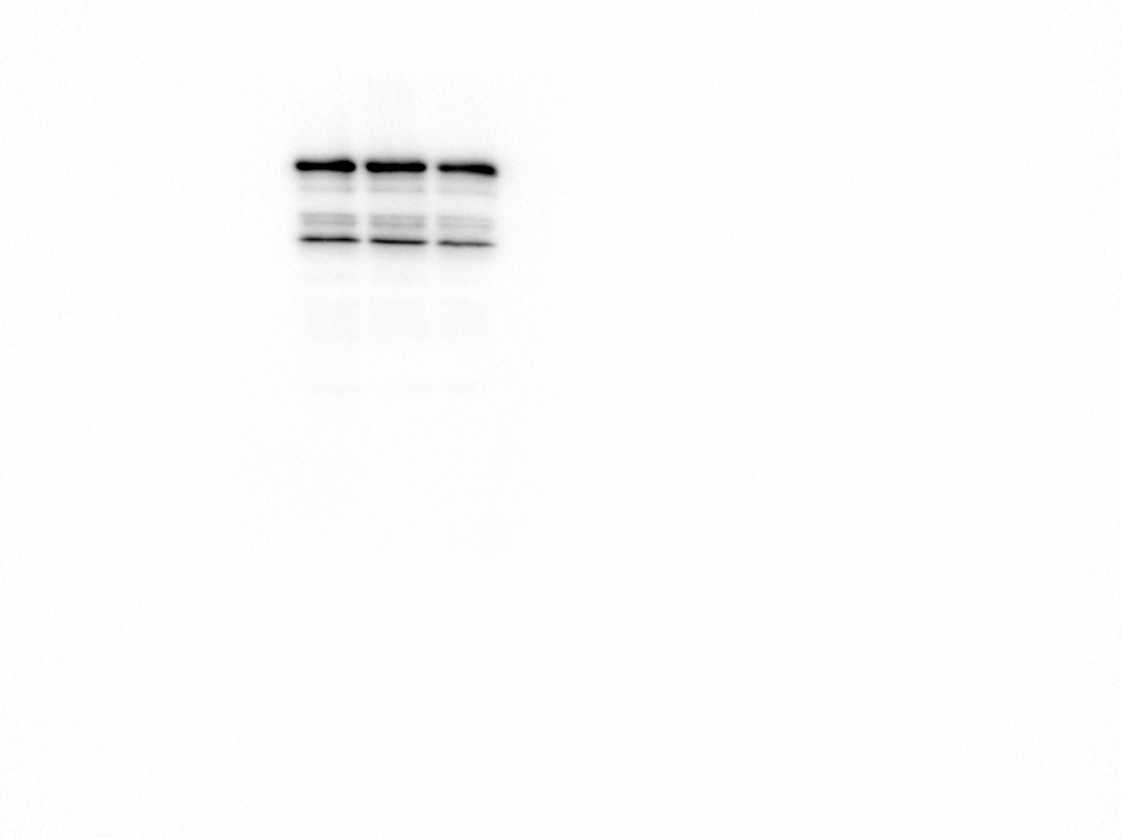

Supplement: Figure 1—source data 4. [file elife-104233-fig1-data4.zip › Figure 1/Fig 1F/Input-anti-Ankle2.tif]

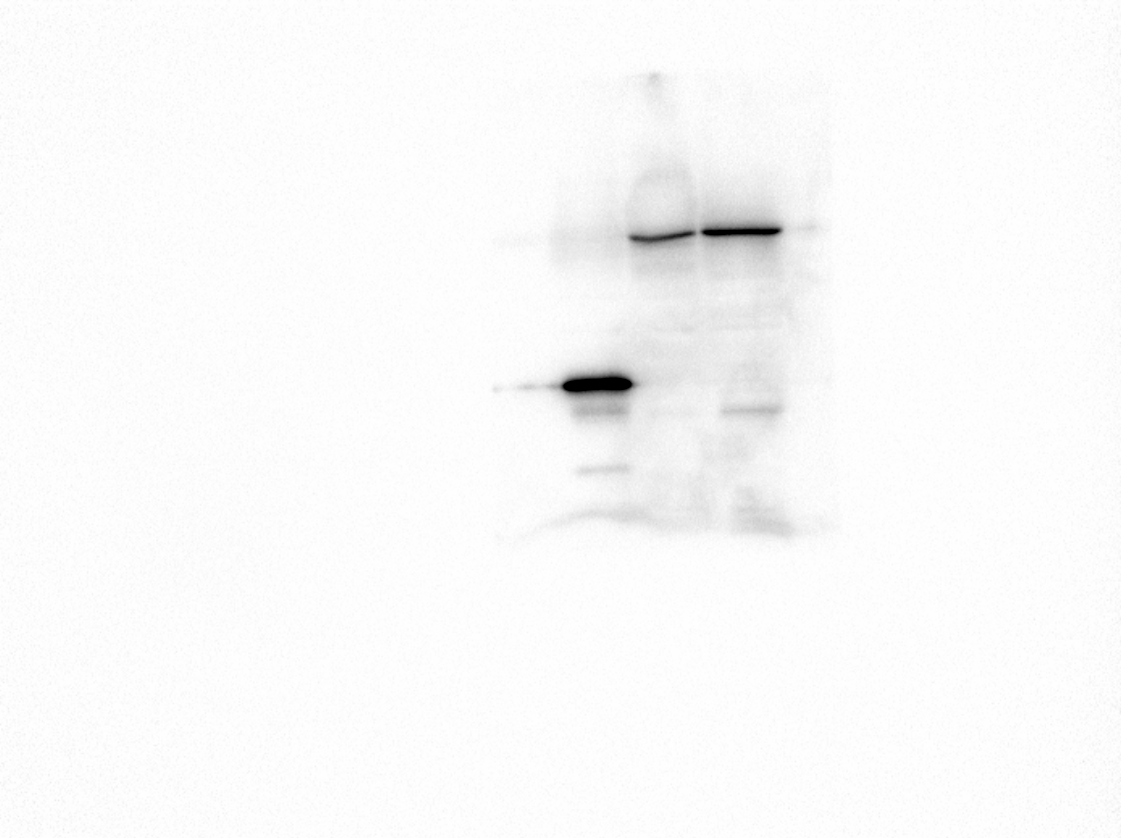

Supplement: Figure 1—source data 4. [file elife-104233-fig1-data4.zip › Figure 1/Fig 1F/Input-anti-GFP.tif]

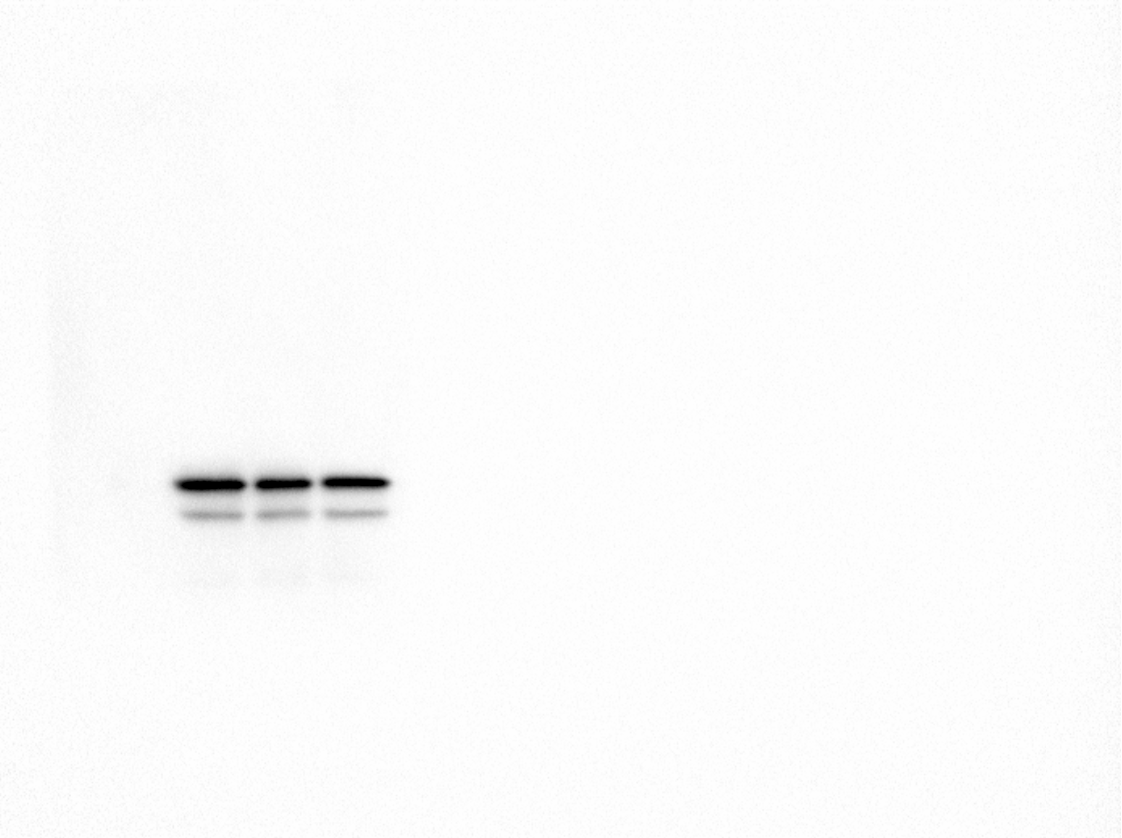

Supplement: Figure 1—source data 4. [file elife-104233-fig1-data4.zip › Figure 1/Fig 1F/Input-anti-Mts.tif]

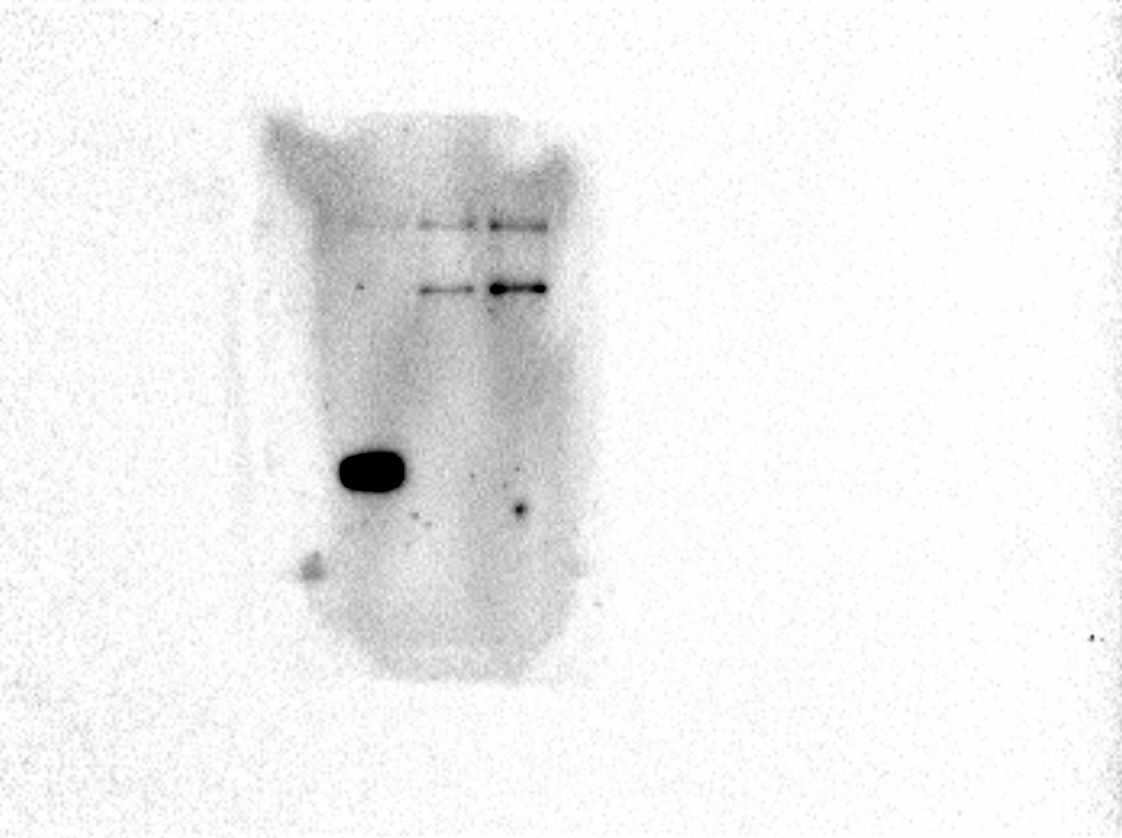

Supplement: Figure 1—source data 4. [file elife-104233-fig1-data4.zip › Figure 1/Fig 1F/Purif-anti-Ankle2.tif]

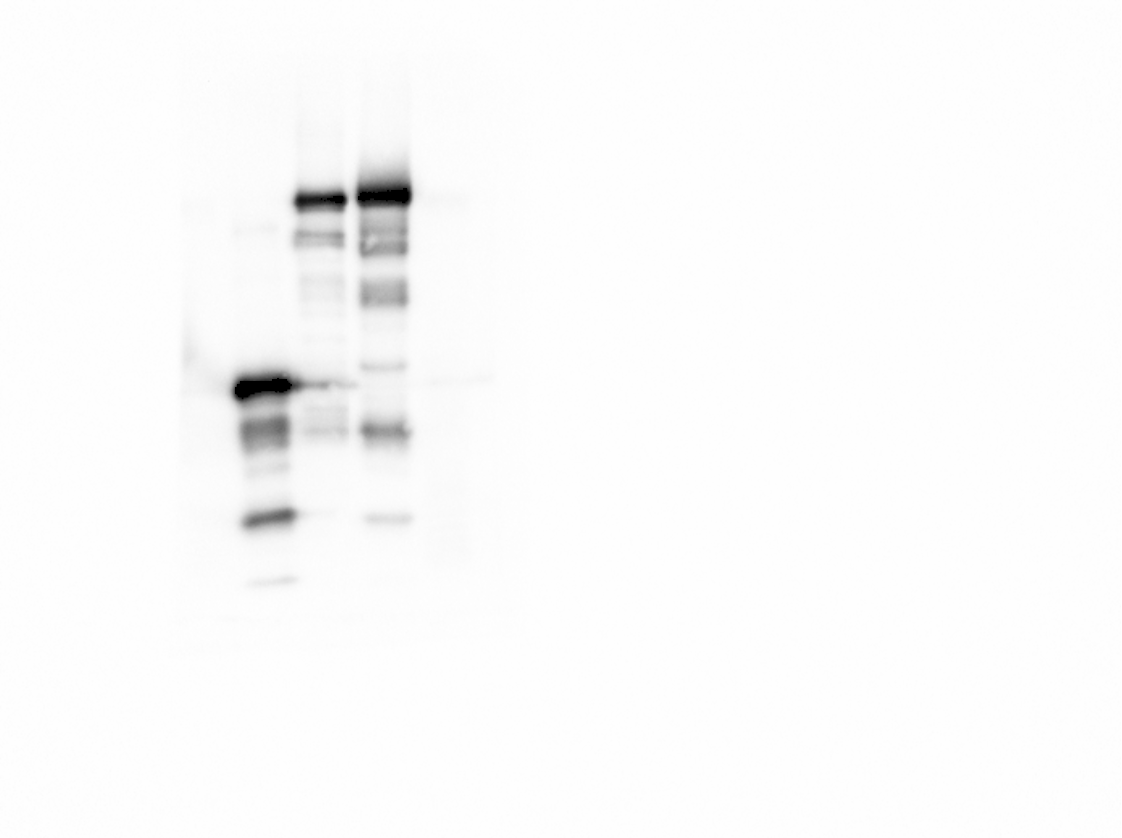

Supplement: Figure 1—source data 4. [file elife-104233-fig1-data4.zip › Figure 1/Fig 1F/Purif-anti-GFP.tif]

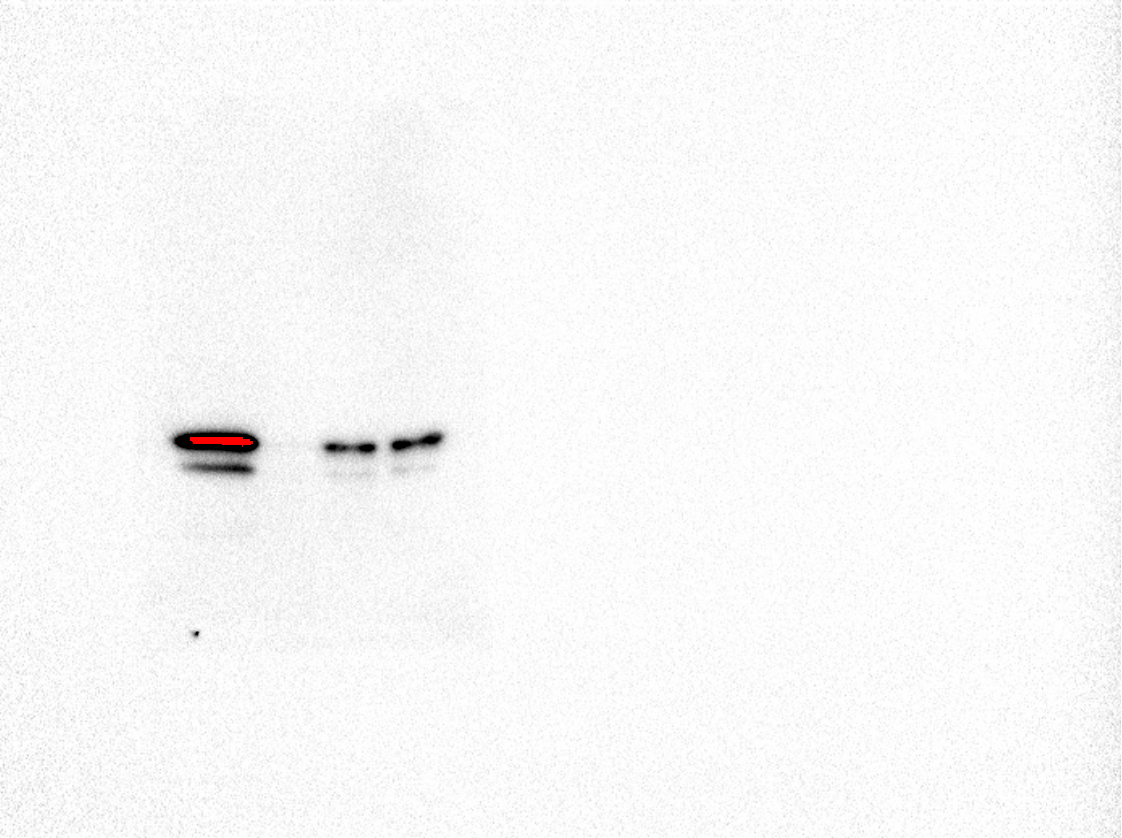

Supplement: Figure 1—source data 4. [file elife-104233-fig1-data4.zip › Figure 1/Fig 1F/Purif-anti-Mts.tif]

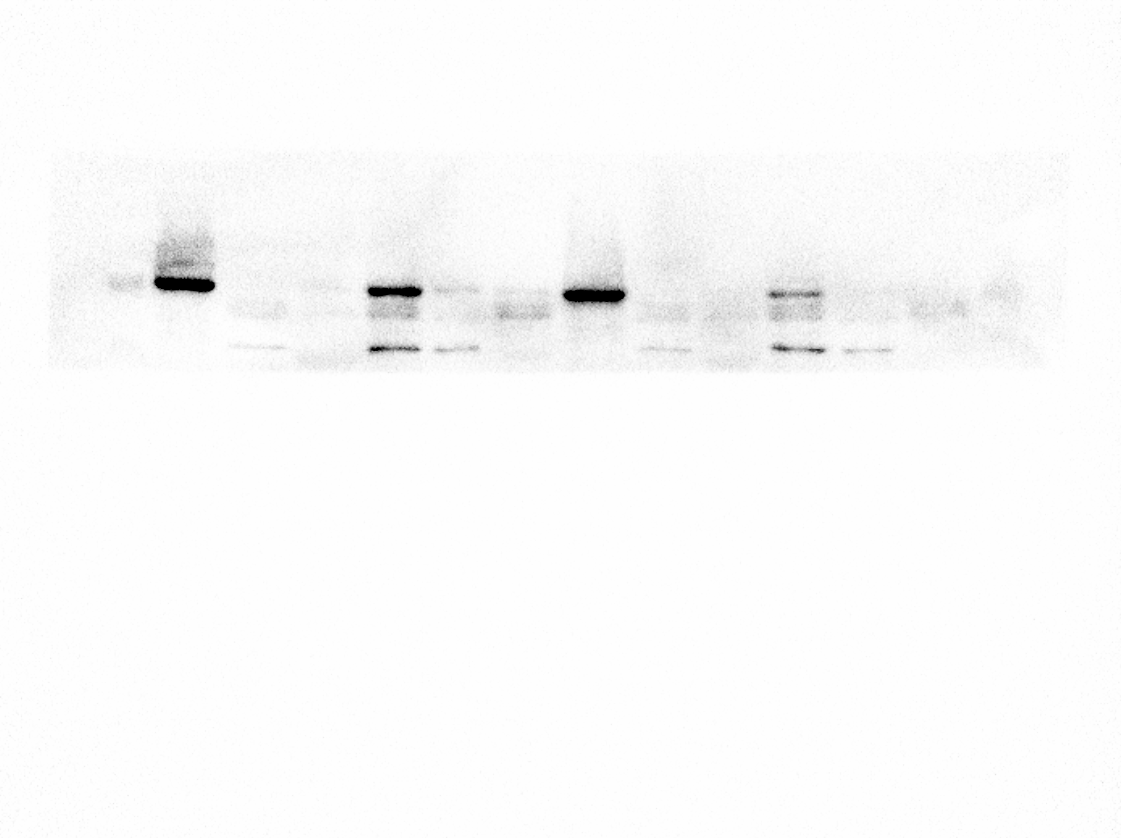

Supplement: Figure 1—source data 4. [file elife-104233-fig1-data4.zip › Figure 1/Fig 1G/anti-GFP.tif]

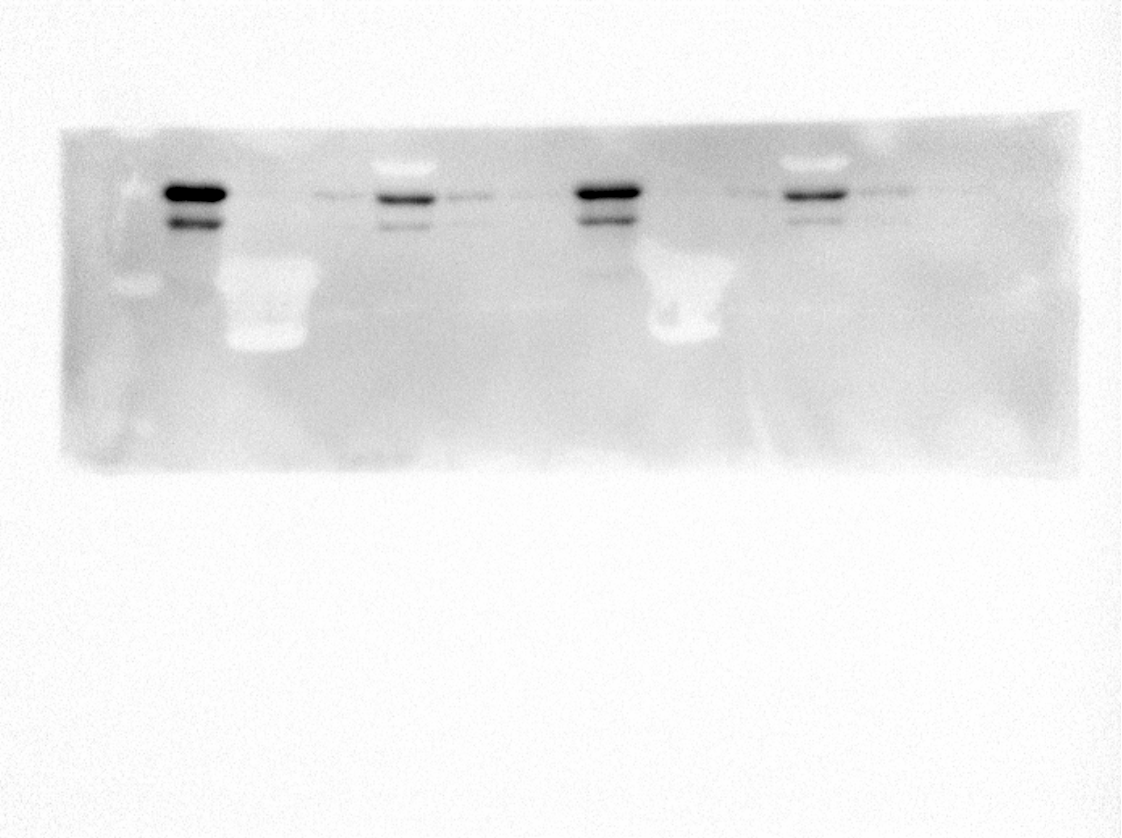

Supplement: Figure 1—source data 4. [file elife-104233-fig1-data4.zip › Figure 1/Fig 1G/anti-Mts.tif]

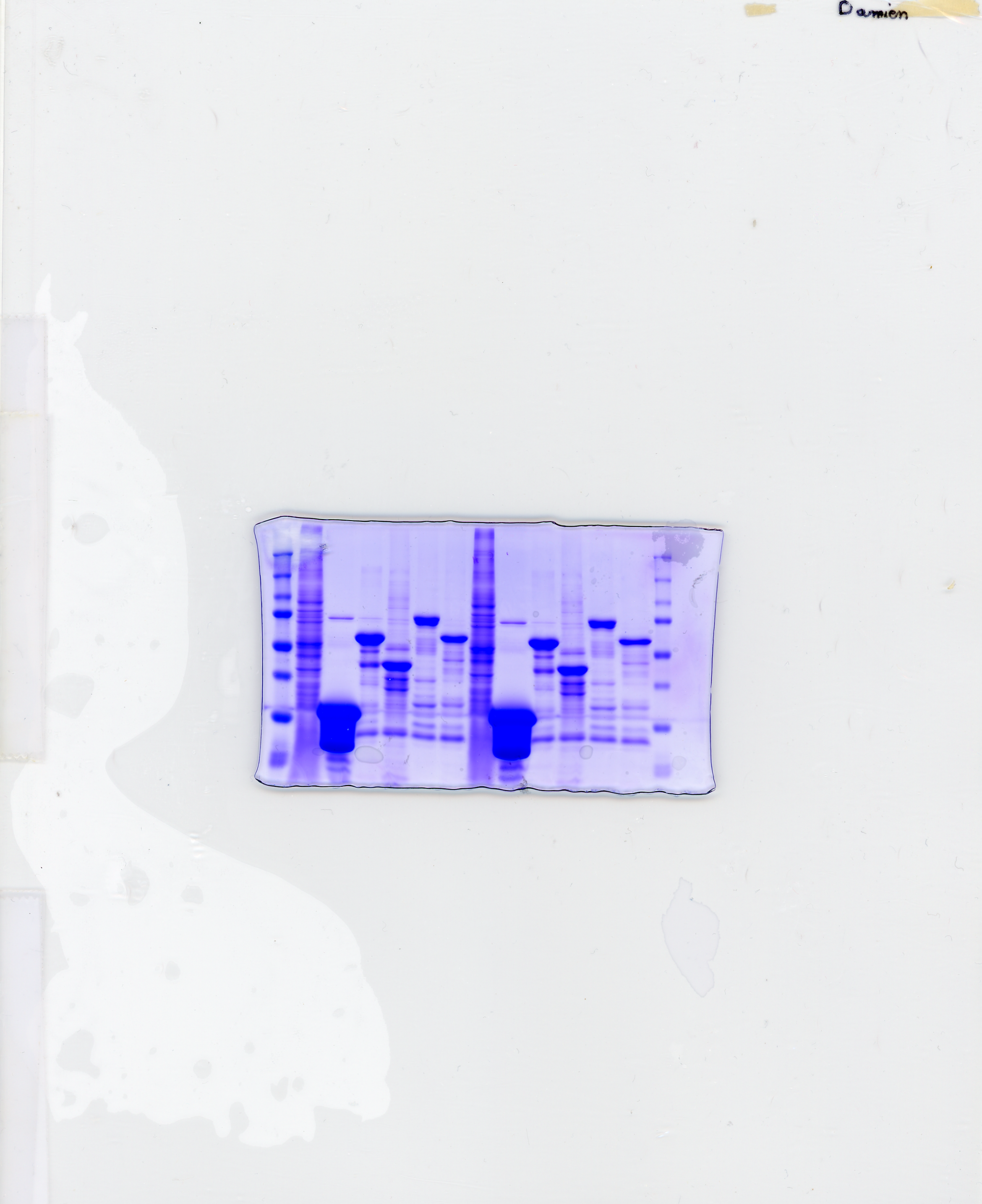

Supplement: Figure 1—source data 4. [file elife-104233-fig1-data4.zip › Figure 1/Fig 1G/CB.tif]

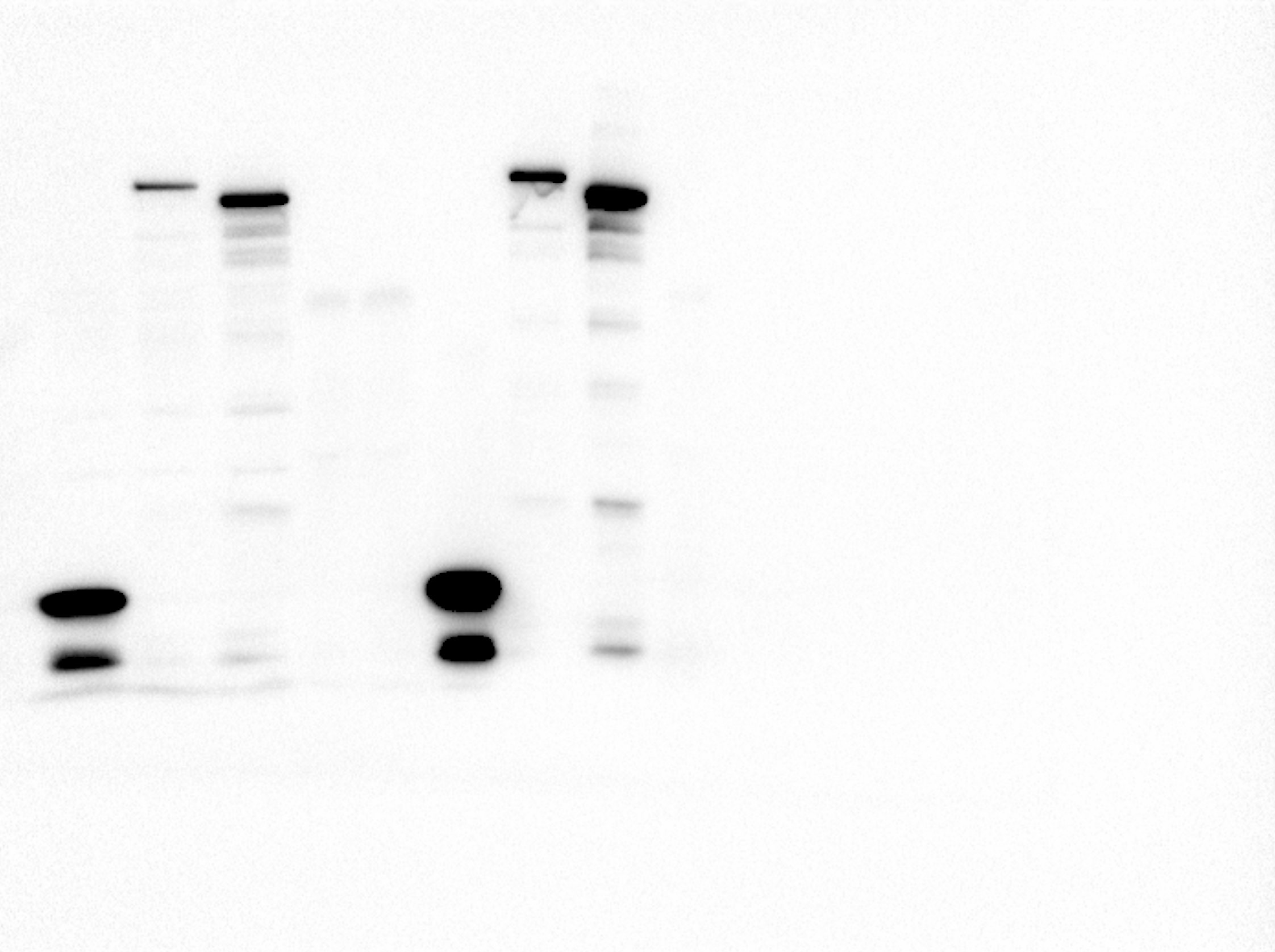

Supplement: Figure 1—source data 4. [file elife-104233-fig1-data4.zip › Figure 1/Fig 1I/Input and purif, anti-GFP.tif]

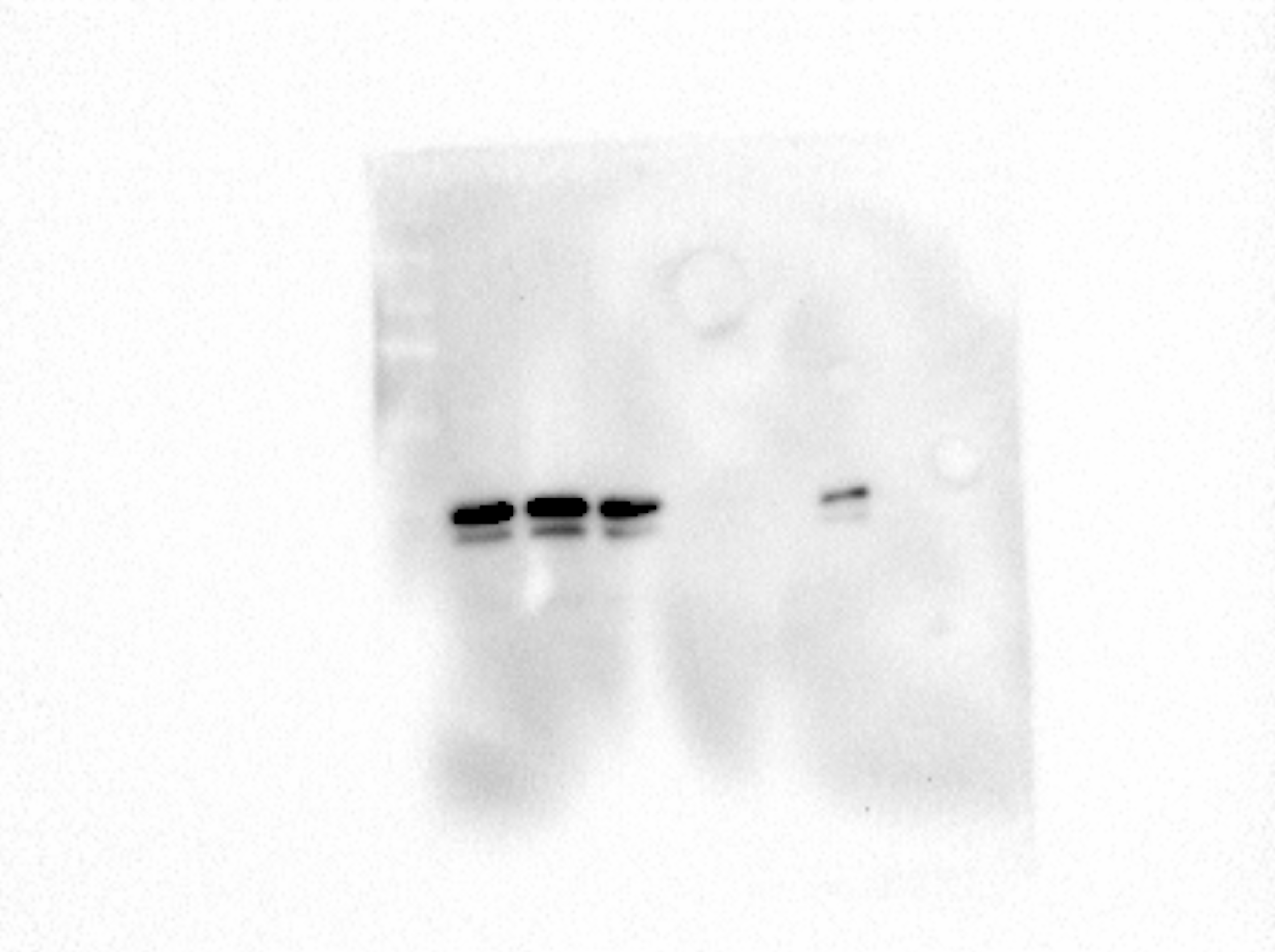

Supplement: Figure 1—source data 4. [file elife-104233-fig1-data4.zip › Figure 1/Fig 1I/Input and purif, anti-Mts.tif]

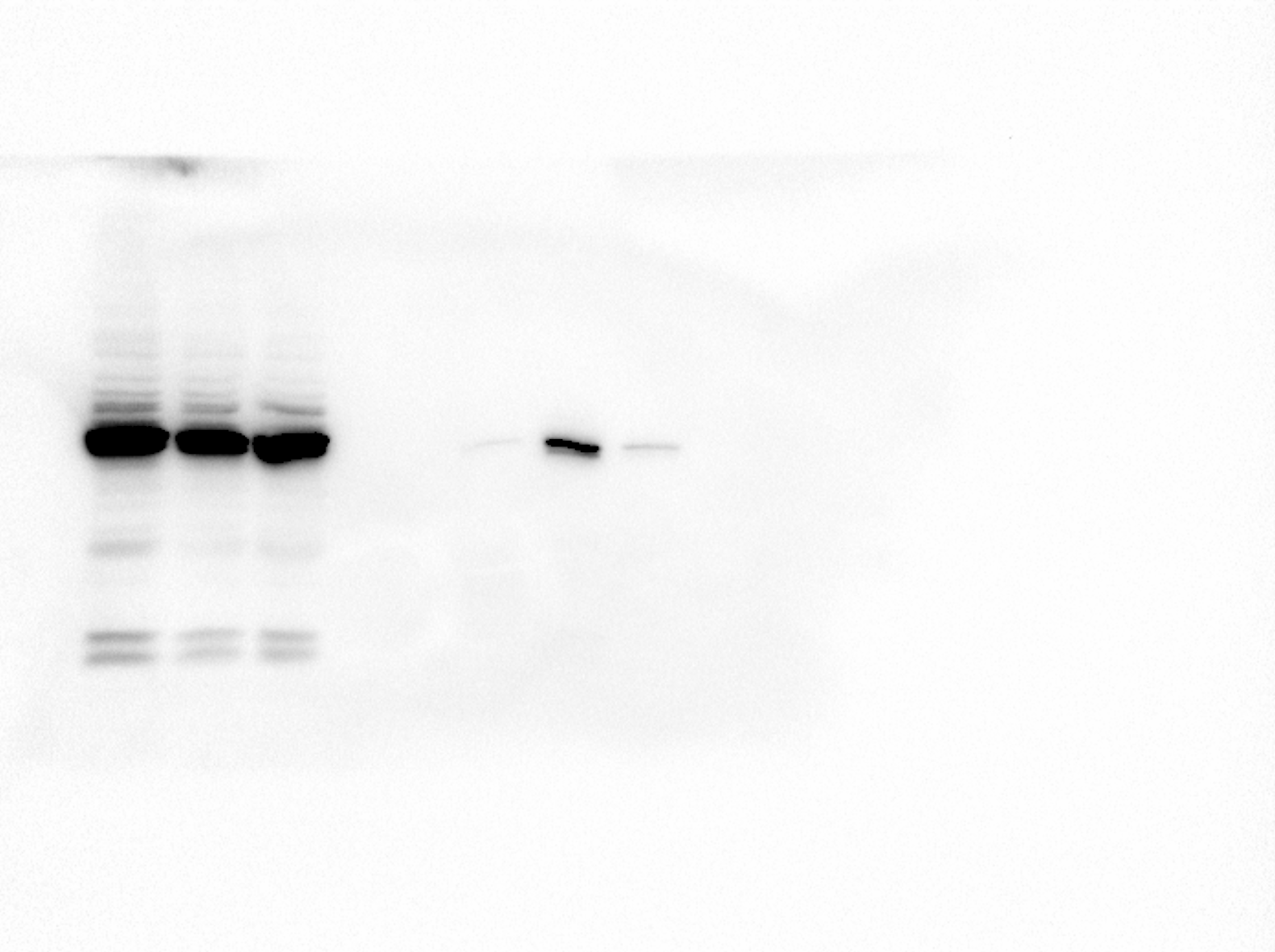

Supplement: Figure 1—source data 4. [file elife-104233-fig1-data4.zip › Figure 1/Fig 1I/Input and purif, anti-Myc.tif]

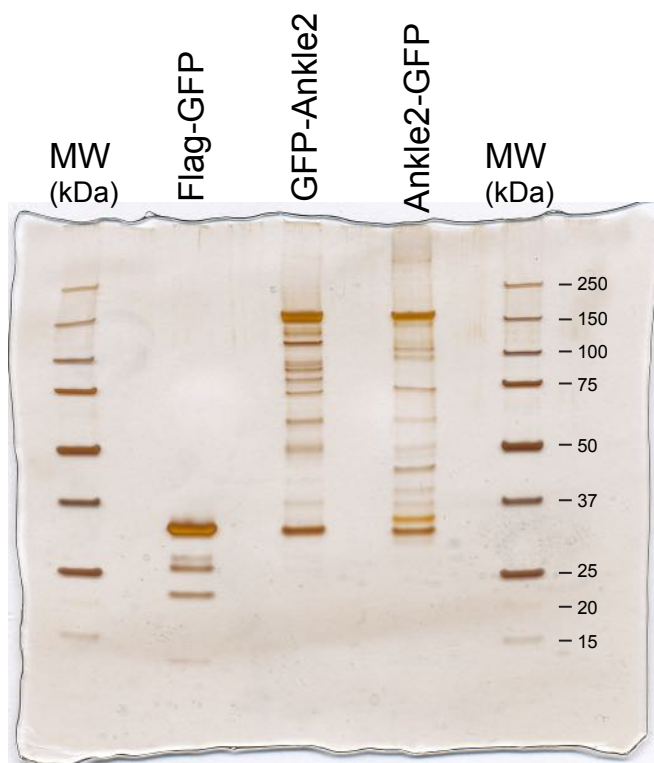

Supplement: Figure 1—figure supplement 1—source data 3. [file elife-104233-fig1-figsupp1-data3.zip › Figure 1 - figure supplement 1/Figure 1 - figure supplement 1A.pdf]

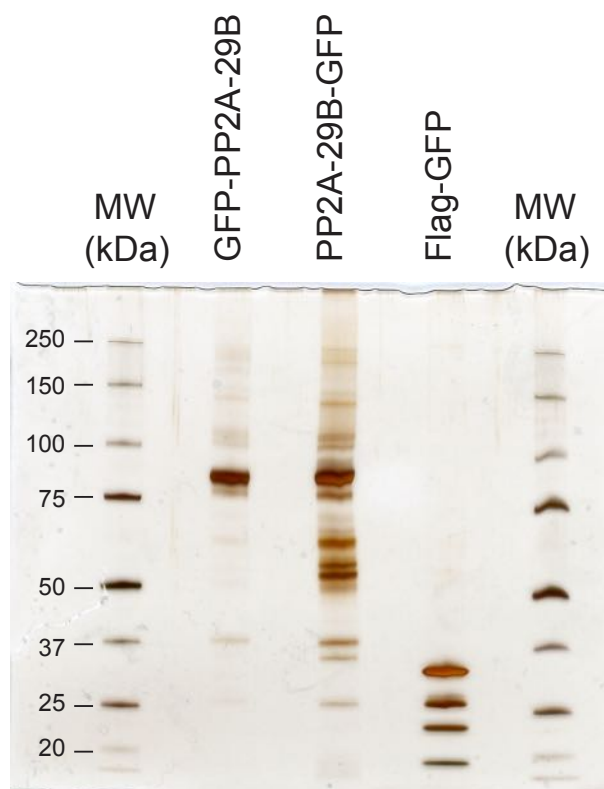

Supplement: Figure 1—figure supplement 1—source data 3. [file elife-104233-fig1-figsupp1-data3.zip › Figure 1 - figure supplement 1/Figure 1 - figure supplement 1C.pdf]

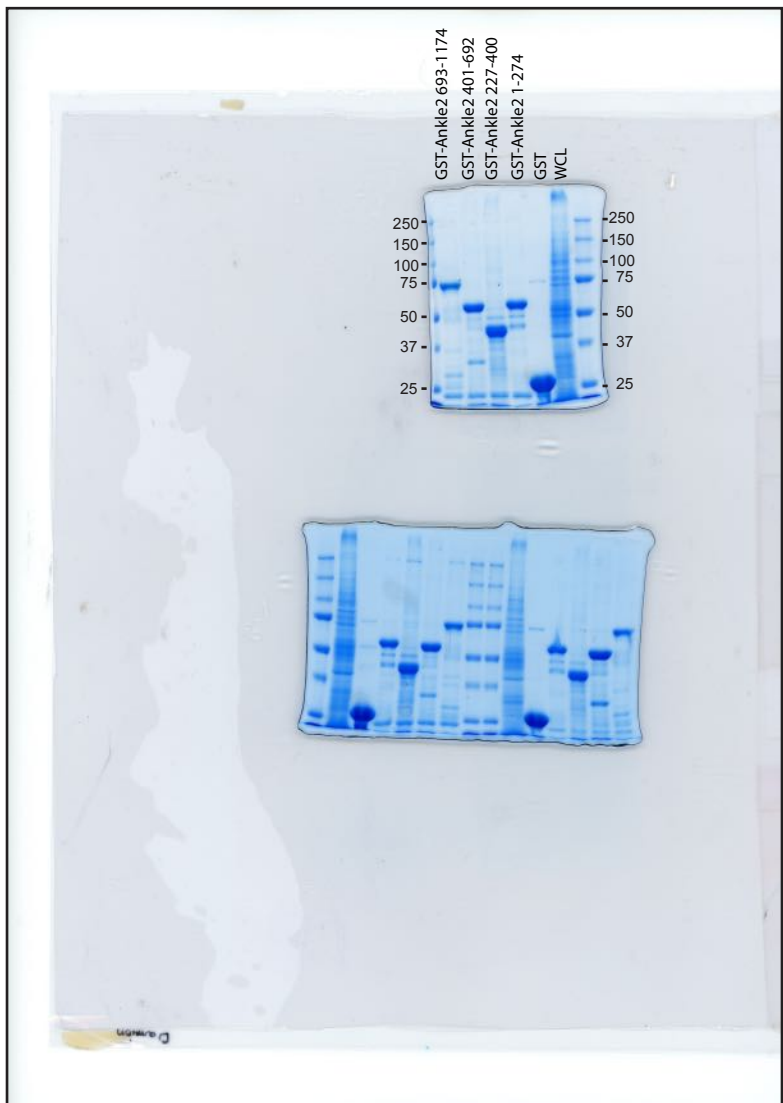

CB

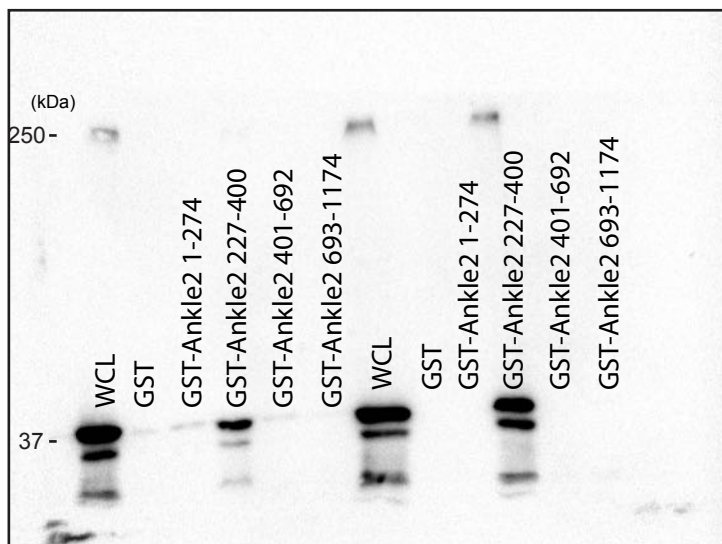

$\alpha$ -Mts

Supplement: Figure 1—figure supplement 1—source data 3. [file elife-104233-fig1-figsupp1-data3.zip › Figure 1 - figure supplement 1/Figure 1 - figure supplement 1E.pdf]

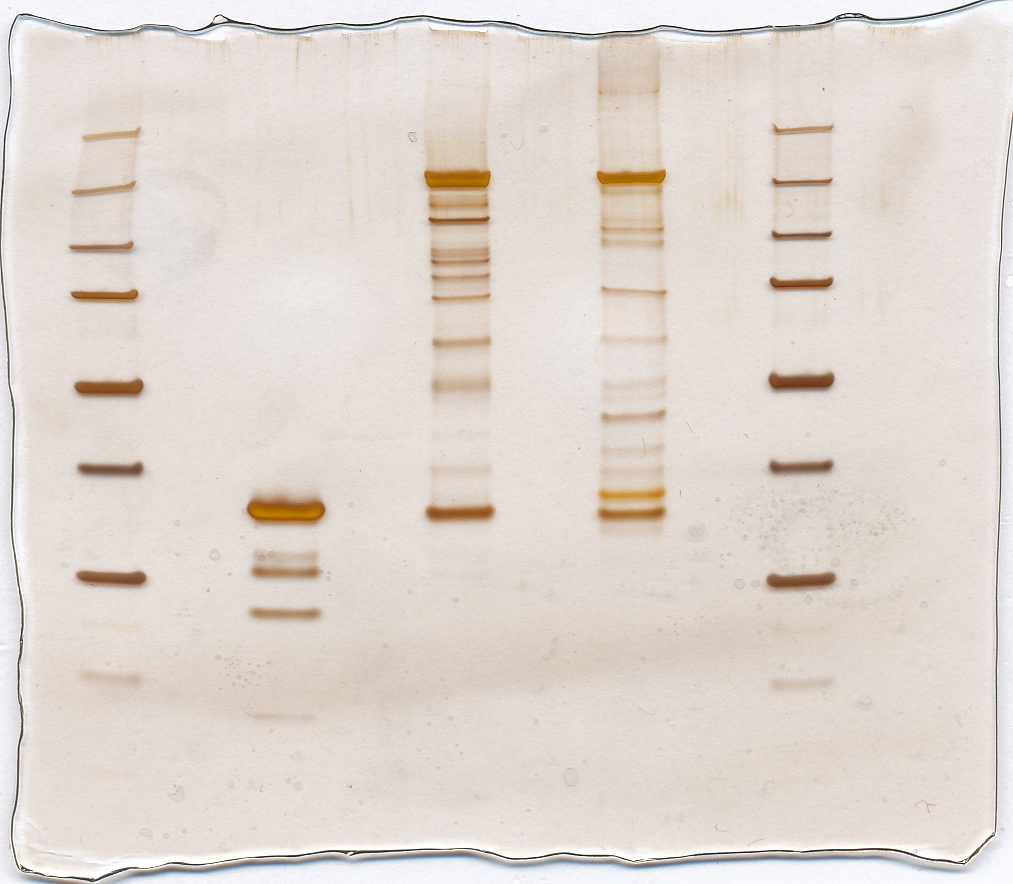

Supplement: Figure 1—figure supplement 1—source data 4. [file elife-104233-fig1-figsupp1-data4.zip › Figure 1 - figure supplement 1/Fig 1 - figure supplement 1A/Ankle2-GFP, silver staining_Dmel.tif]

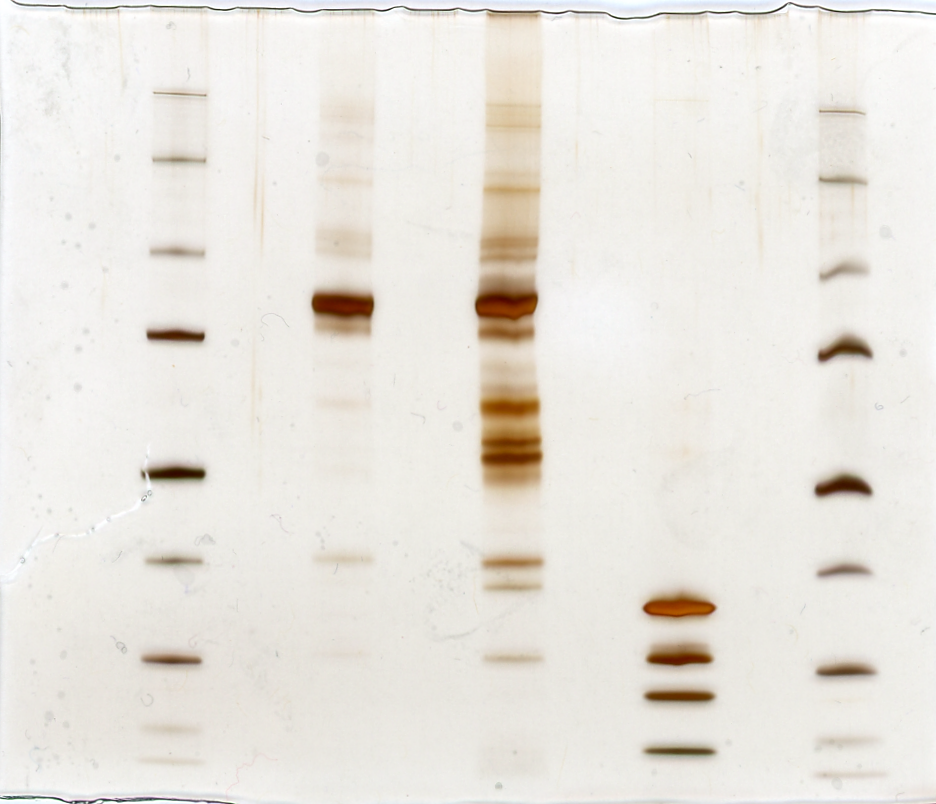

Supplement: Figure 1—figure supplement 1—source data 4. [file elife-104233-fig1-figsupp1-data4.zip › Figure 1 - figure supplement 1/Fig 1 - figure supplement 1C/PP2A-29B silver staining.tif]

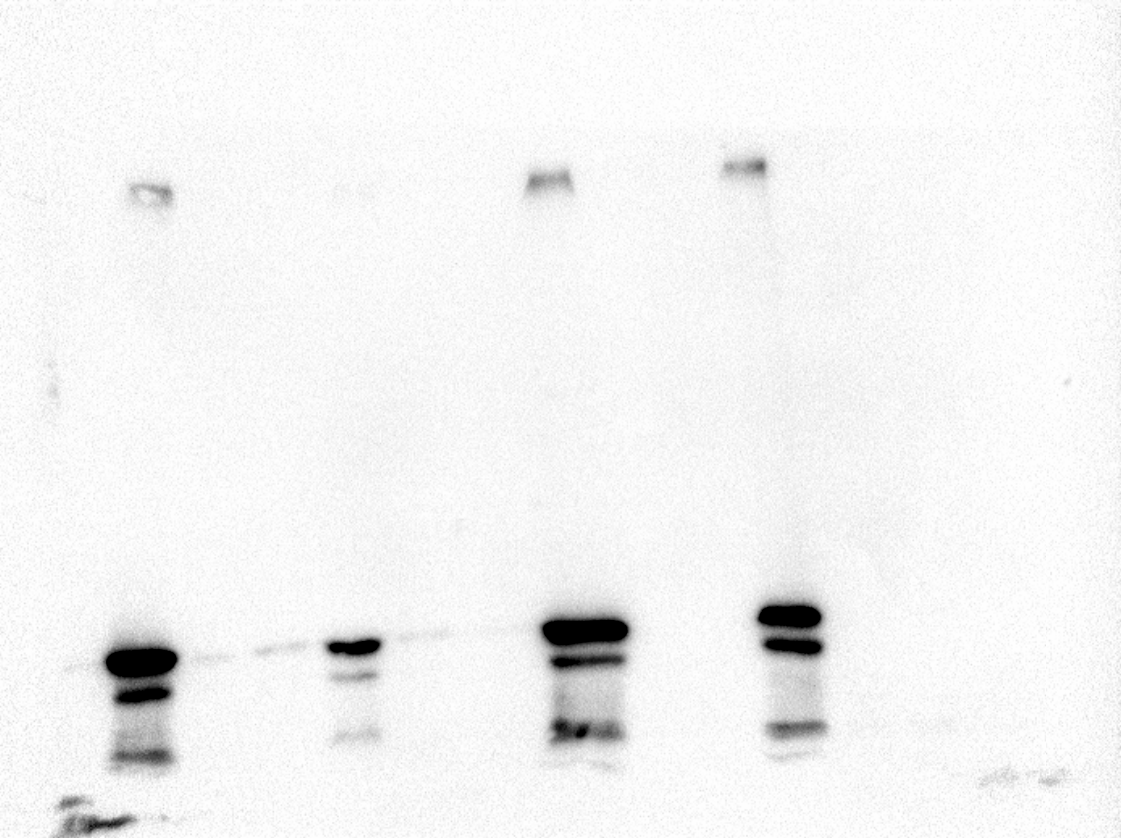

Supplement: Figure 1—figure supplement 1—source data 4. [file elife-104233-fig1-figsupp1-data4.zip › Figure 1 - figure supplement 1/Fig 1 - figure supplement 1E/anti-Mts.tif]

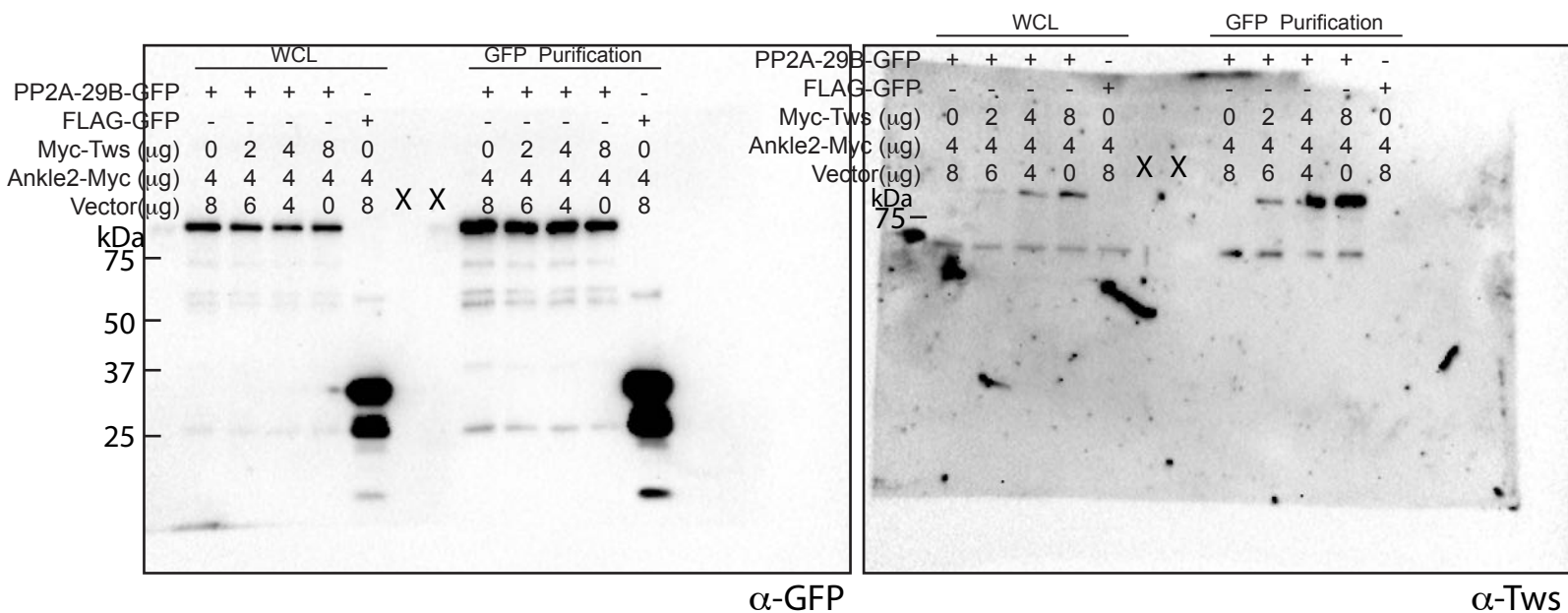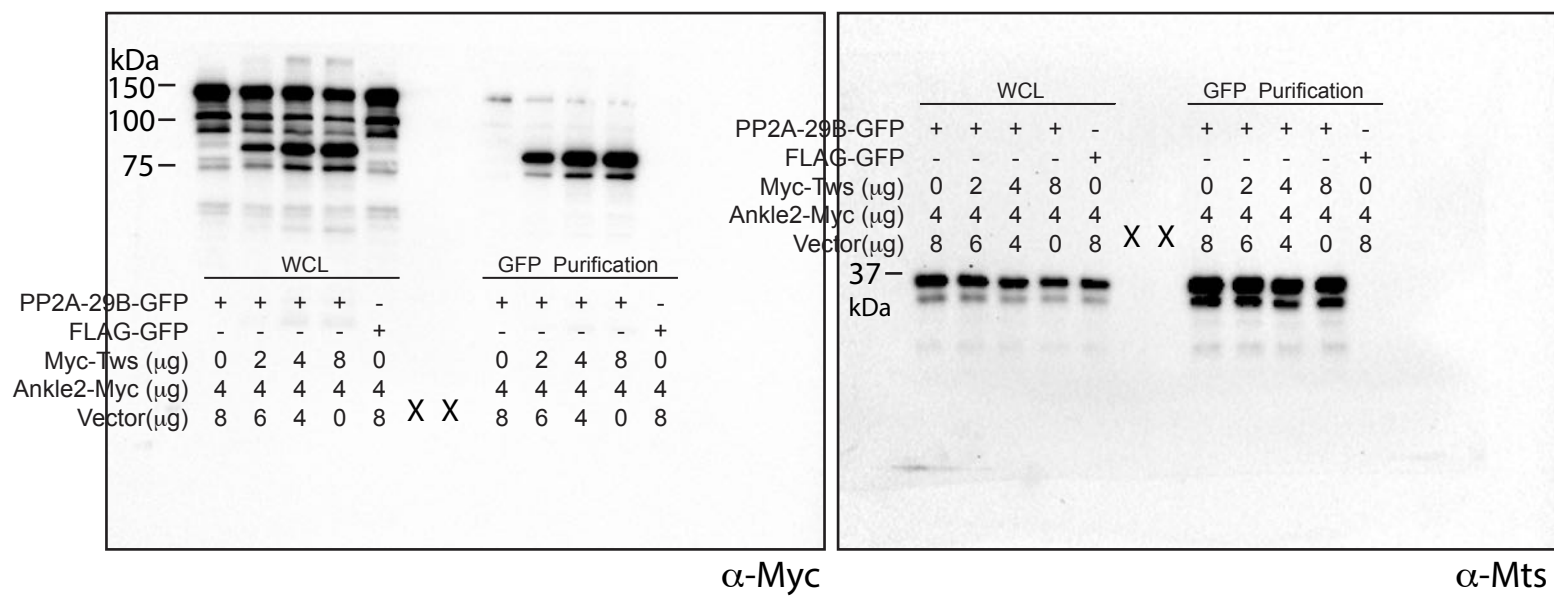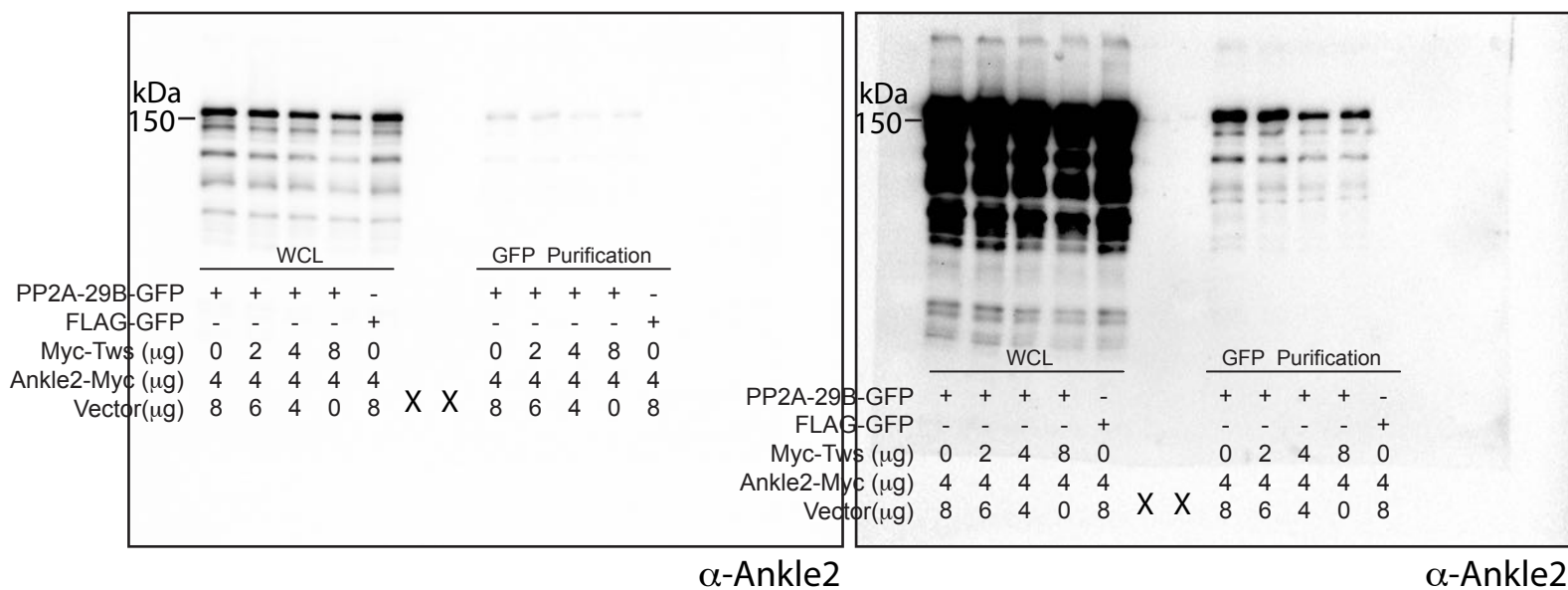

Supplement: Figure 2—source data 3. [file elife-104233-fig2-data3.zip › Figure 2/Figure 2B.pdf]

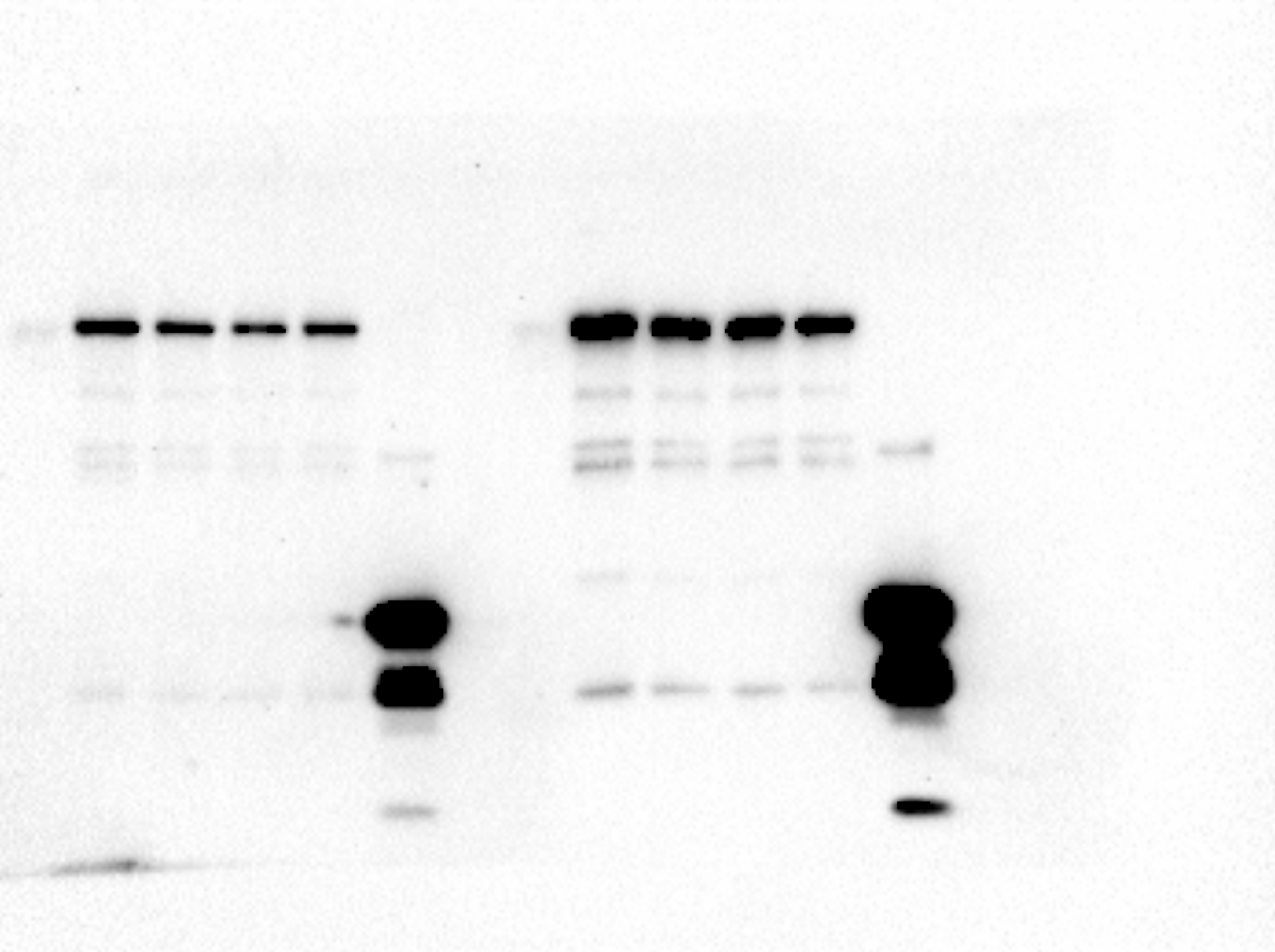

Supplement: Figure 2—source data 4. [file elife-104233-fig2-data4.zip › Figure 2/Input and purif, anti-GFP.tif]

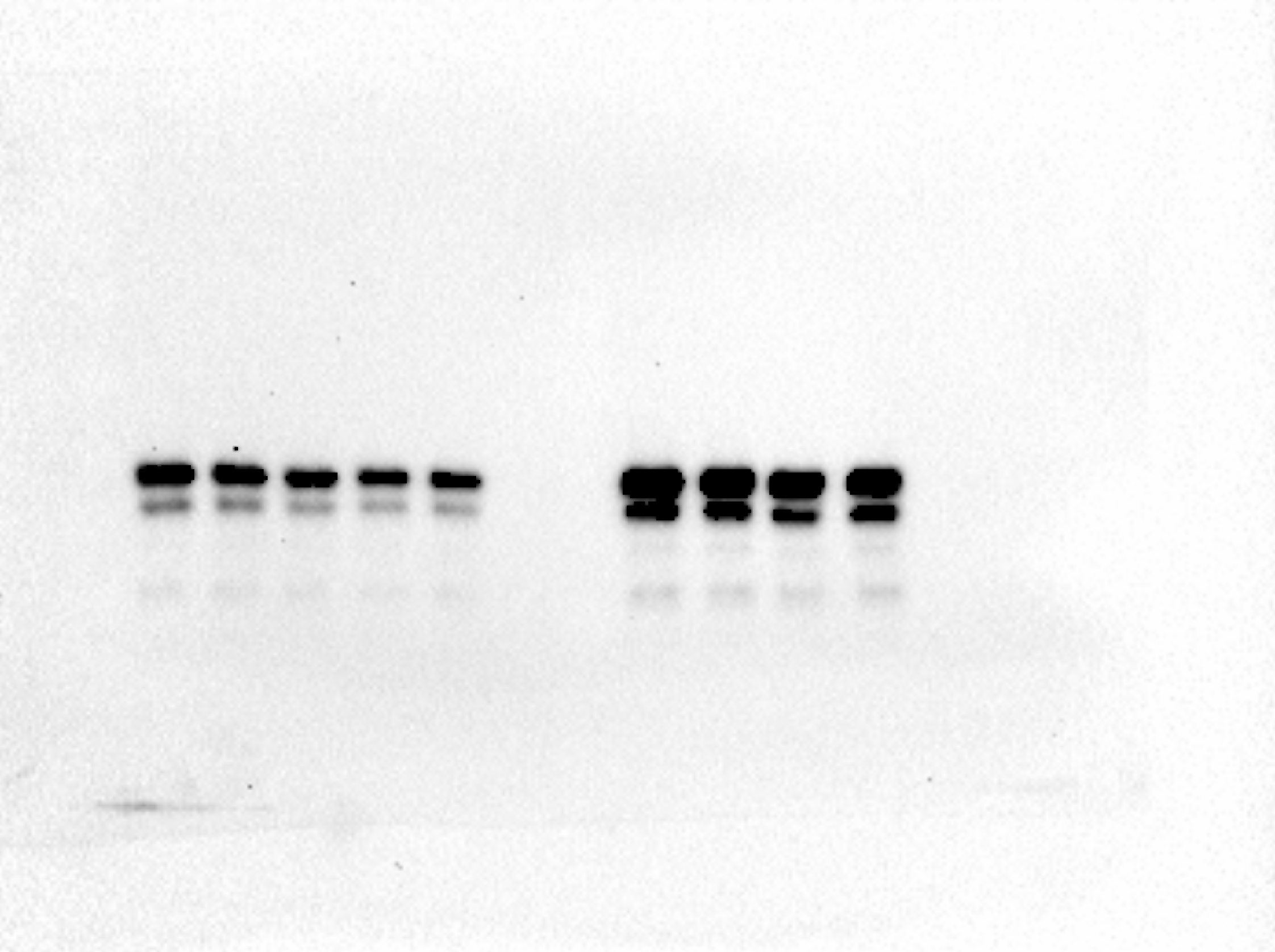

Supplement: Figure 2—source data 4. [file elife-104233-fig2-data4.zip › Figure 2/Input and purif, anti-Mts.tif]

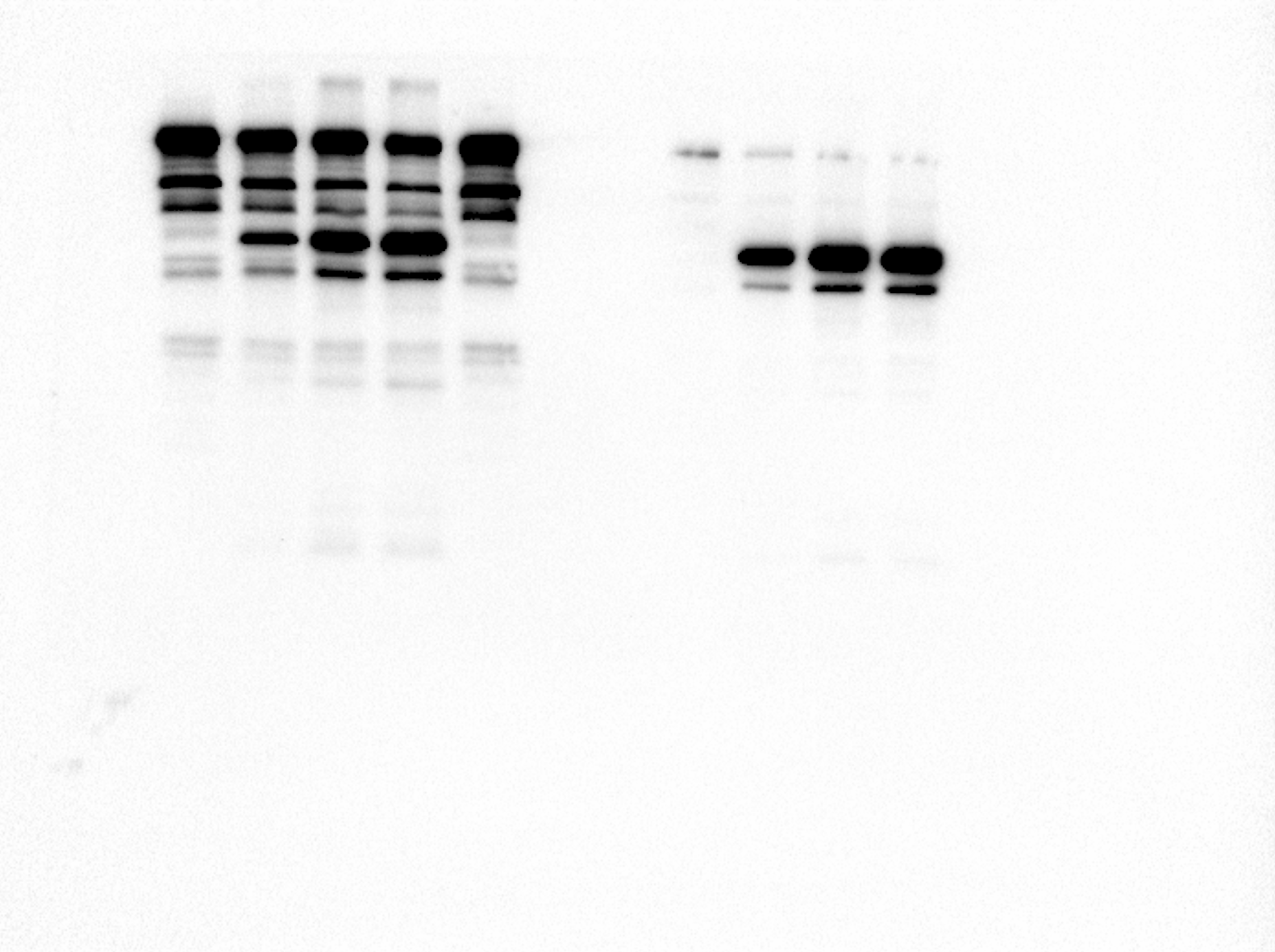

Supplement: Figure 2—source data 4. [file elife-104233-fig2-data4.zip › Figure 2/Input and purif, anti-Myc.tif]

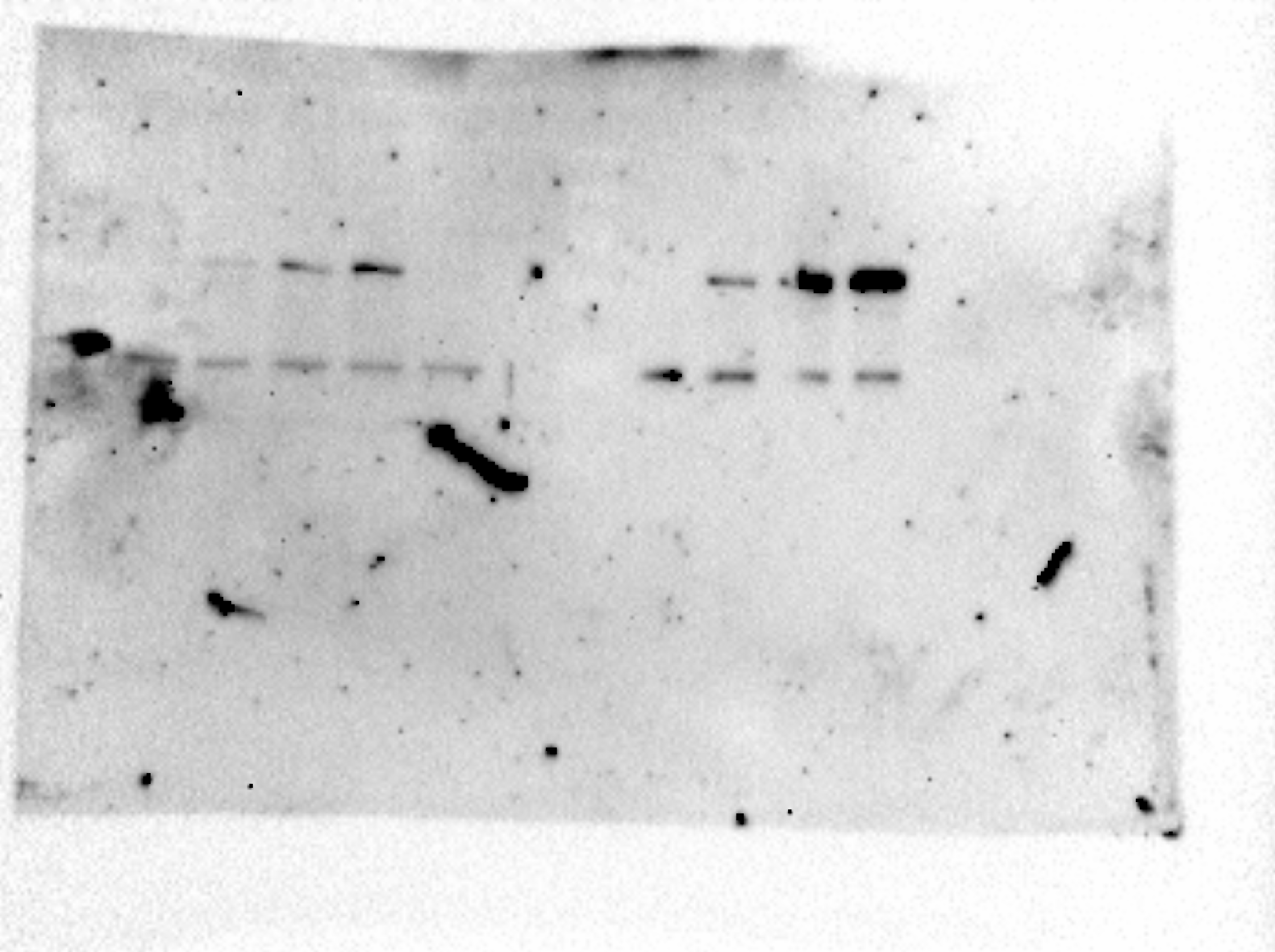

Supplement: Figure 2—source data 4. [file elife-104233-fig2-data4.zip › Figure 2/Input and purif, anti-Tws.tif]

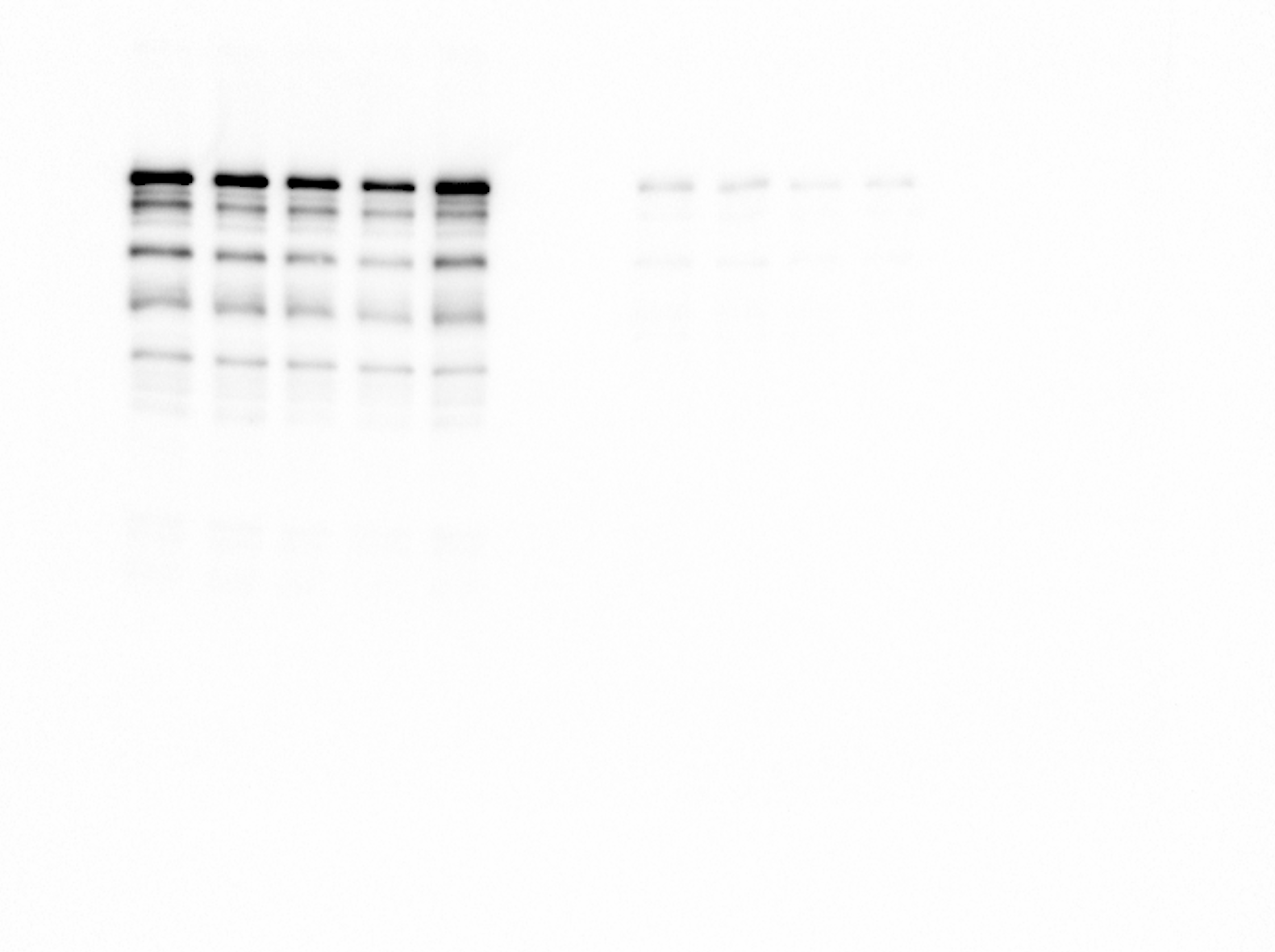

Supplement: Figure 2—source data 4. [file elife-104233-fig2-data4.zip › Figure 2/Input-anti-Ankle2.tif]

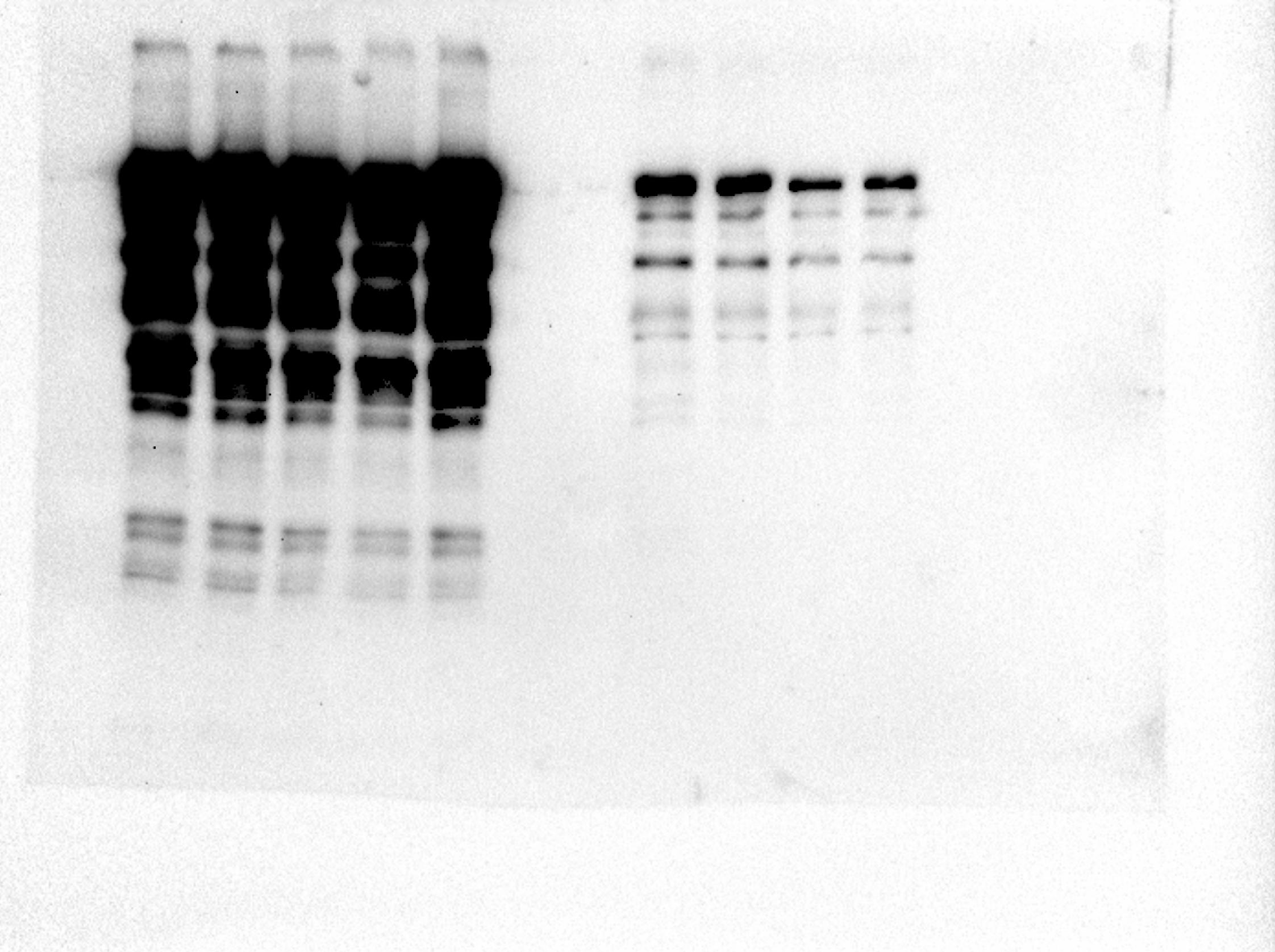

Supplement: Figure 2—source data 4. [file elife-104233-fig2-data4.zip › Figure 2/Purif-anti-Ankle2.tif]

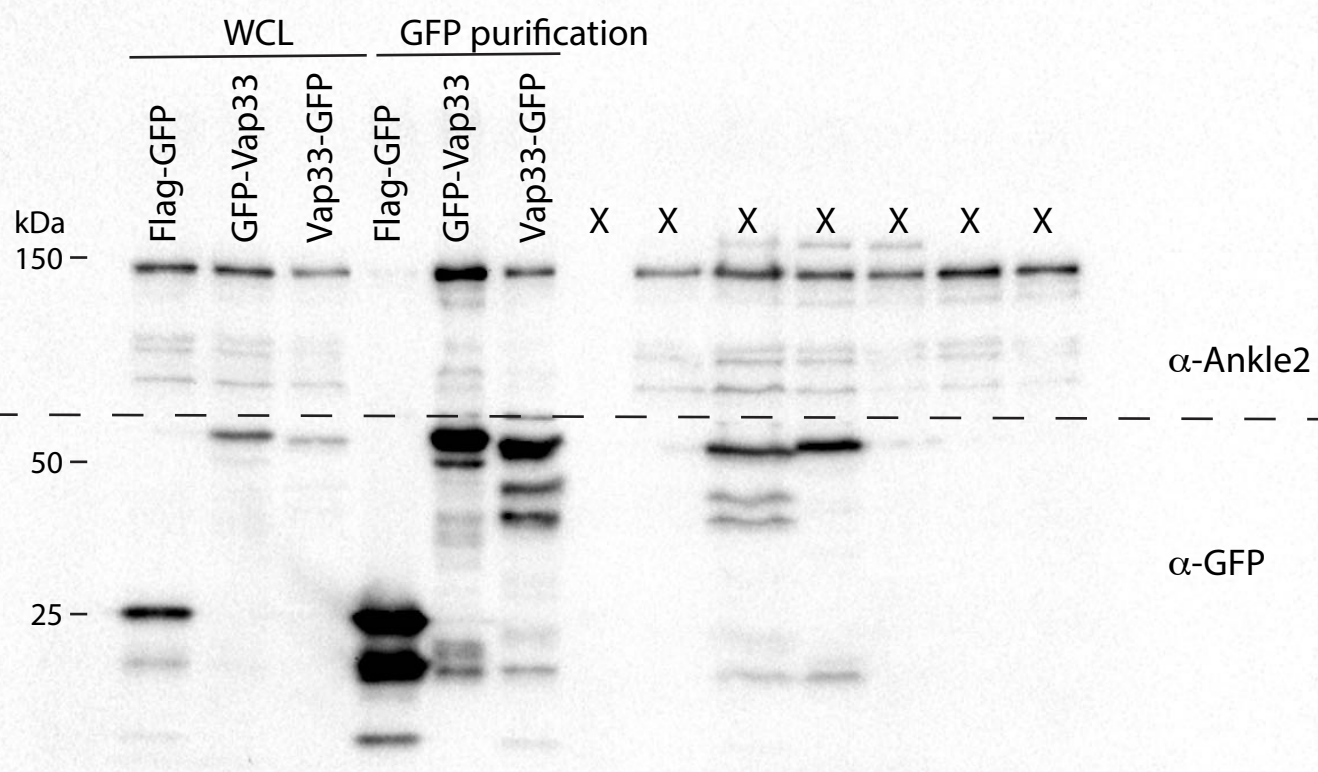

Supplement: Figure 4—source data 1. [file elife-104233-fig4-data1.zip › Figure 4/Figure 4A.pdf]

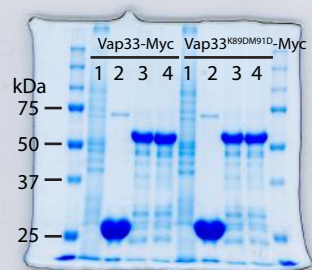

1: WCL  
2: GST  
3: GST-Ankle2 910-1174  
4: GST-Ankle2 910-1174+Fm

CB

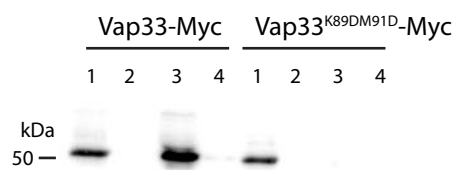

1: WCL  
2: GST  
3: GST-Ankle2 910-1174  
4: GST-Ankle2 910-1174+Fm

$\alpha$ -Myc

Supplement: Figure 4—source data 1. [file elife-104233-fig4-data1.zip › Figure 4/Figure 4C.pdf]

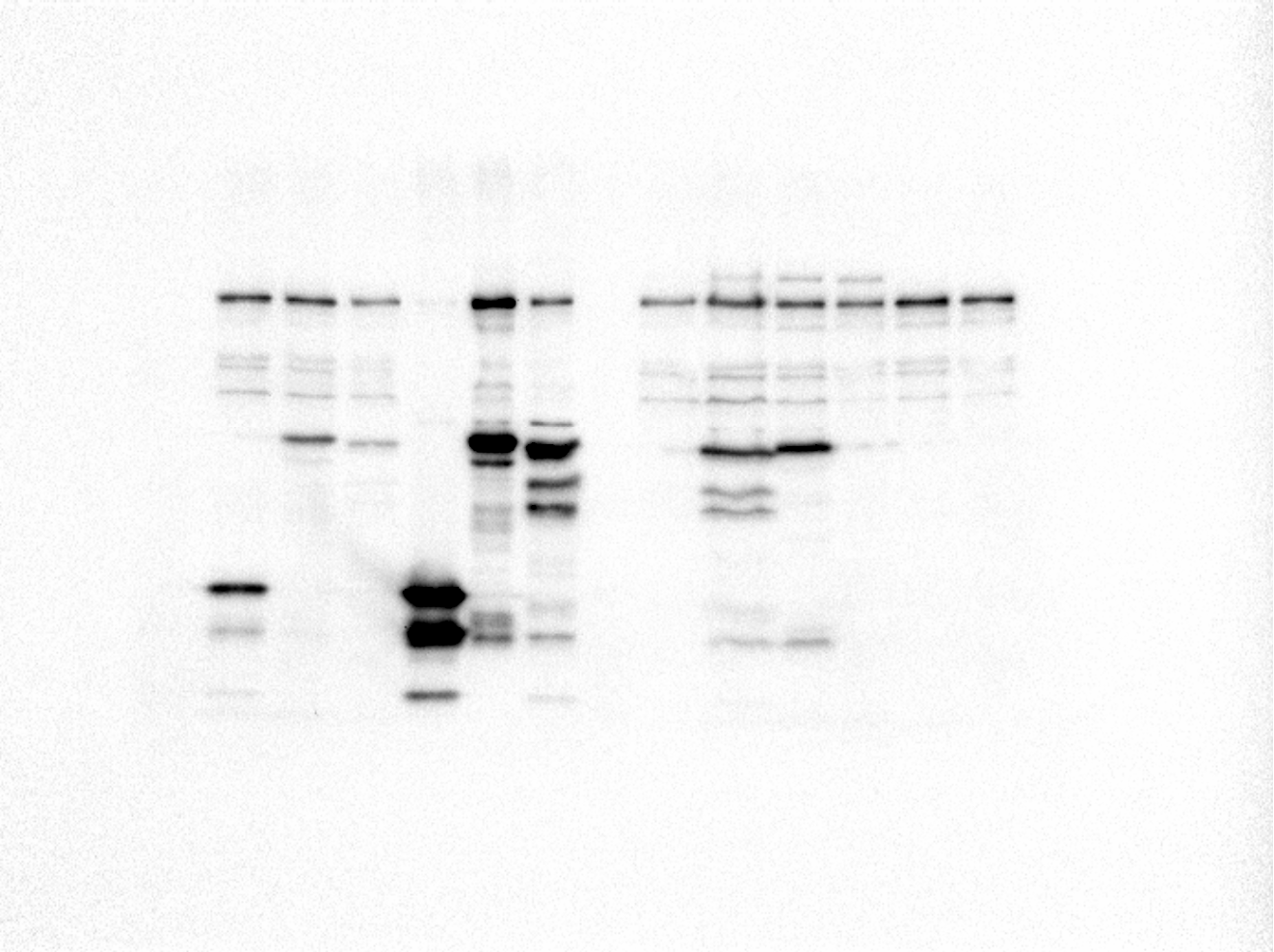

Supplement: Figure 4—source data 2. [file elife-104233-fig4-data2.zip › Figure 4/Fig 4A/Input and purif, anti-Ankle2(top), anti-GFP(bottom).tif]

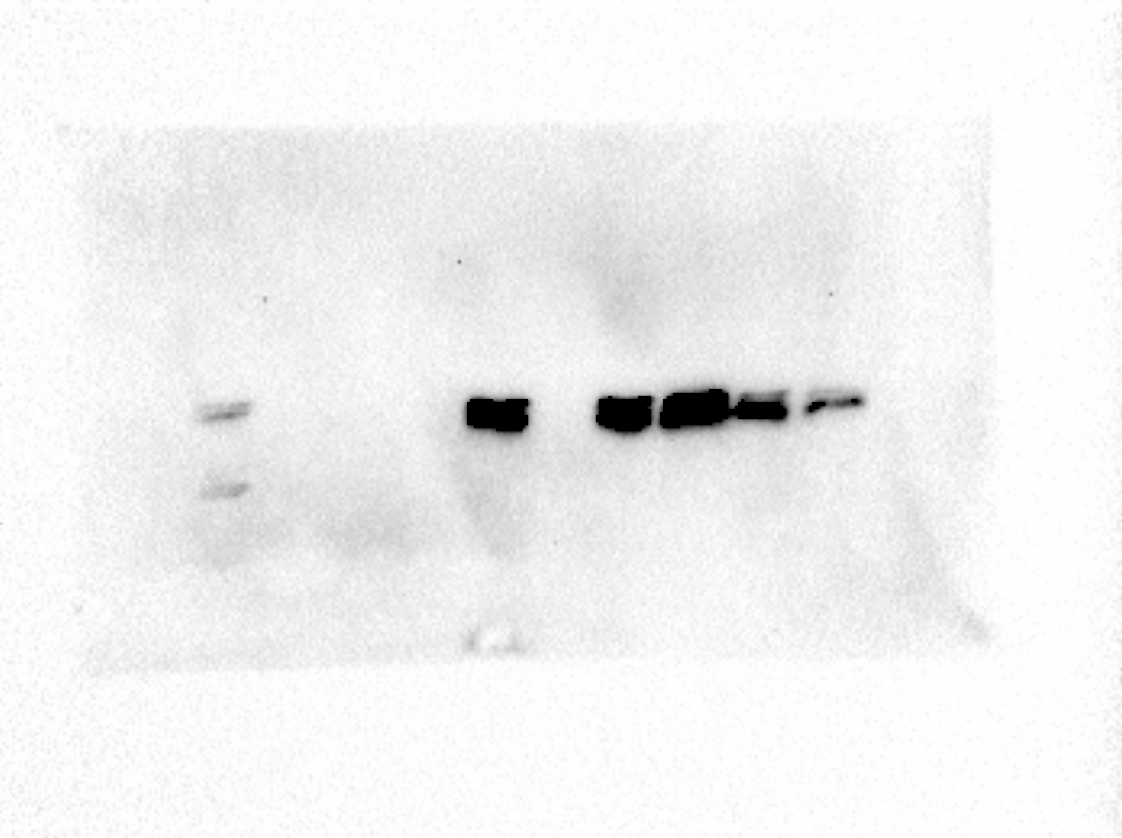

Supplement: Figure 4—source data 2. [file elife-104233-fig4-data2.zip › Figure 4/Fig 4B/anti-Myc.tif]

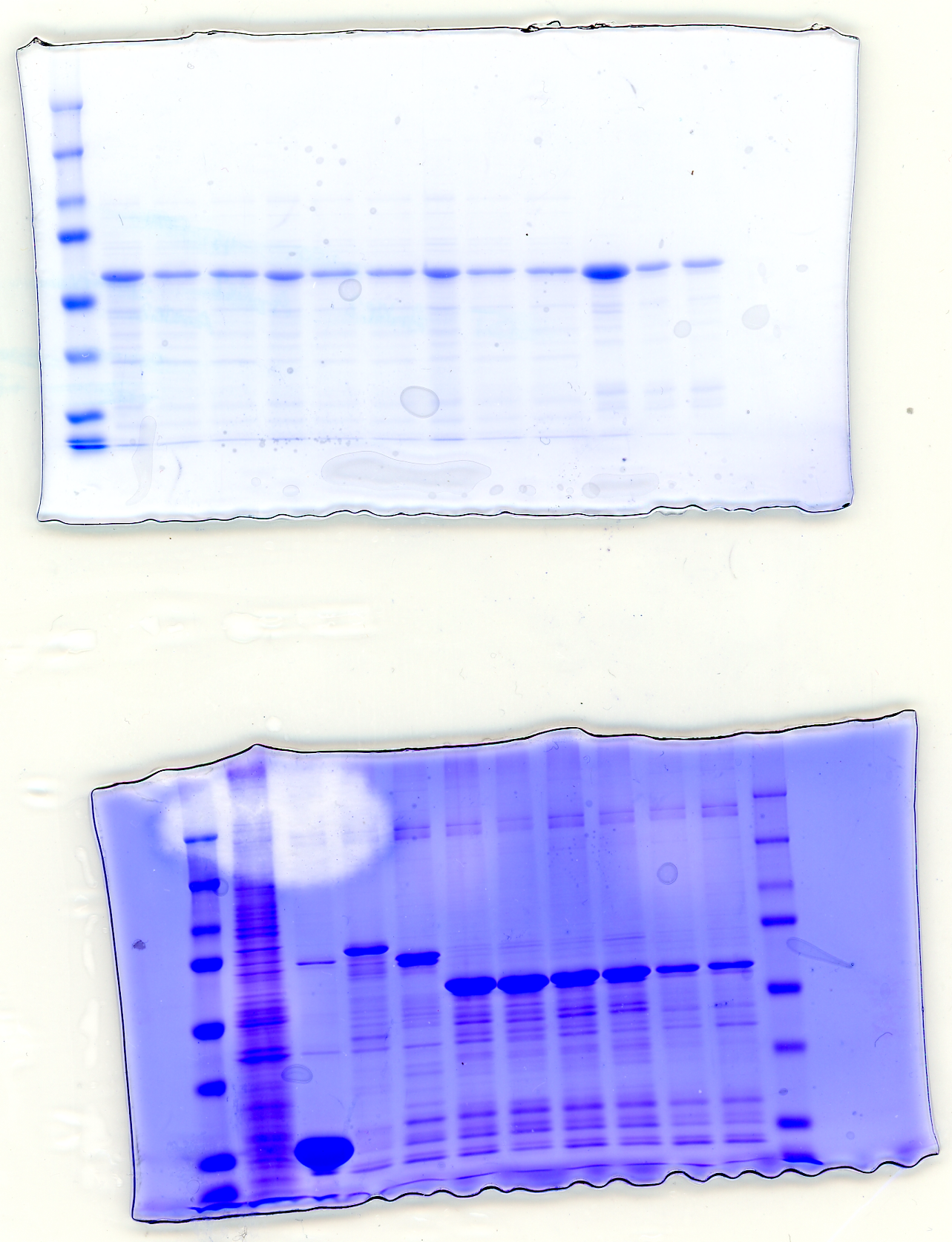

Supplement: Figure 4—source data 2. [file elife-104233-fig4-data2.zip › Figure 4/Fig 4B/CB.tif]

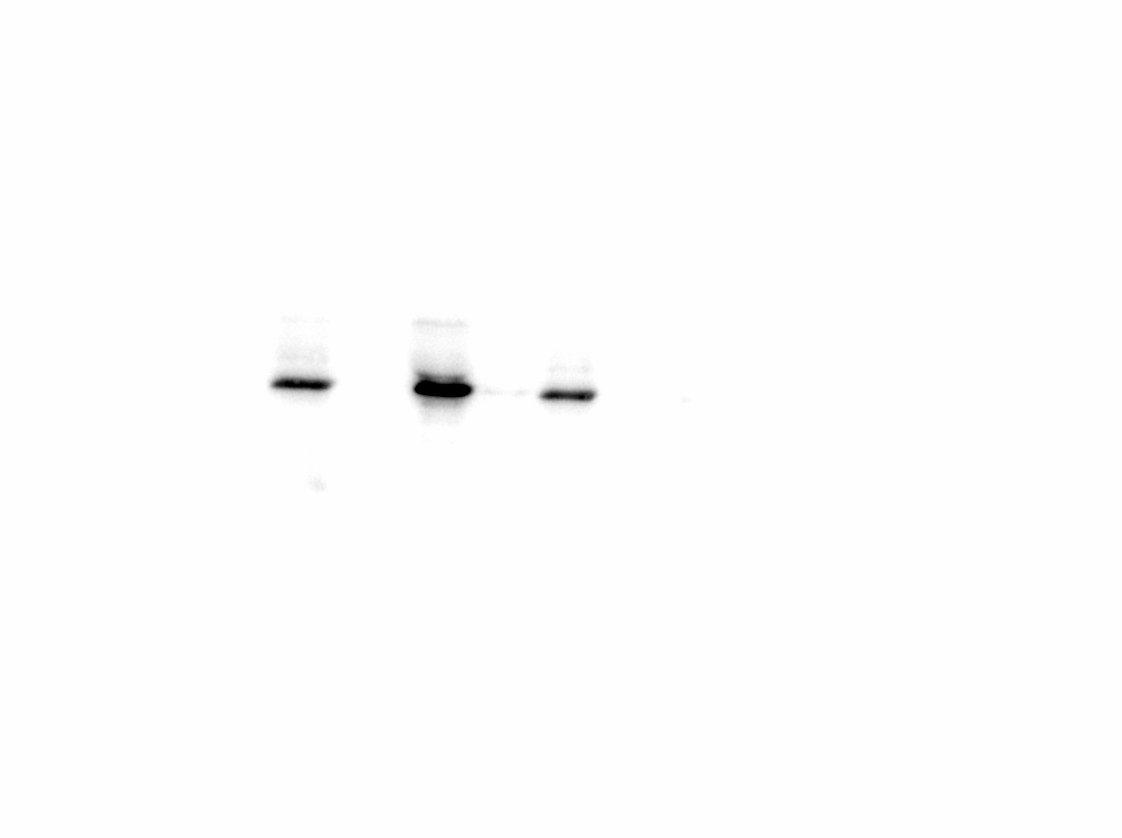

Supplement: Figure 4—source data 2. [file elife-104233-fig4-data2.zip › Figure 4/Fig 4C/anti-Myc.tif]

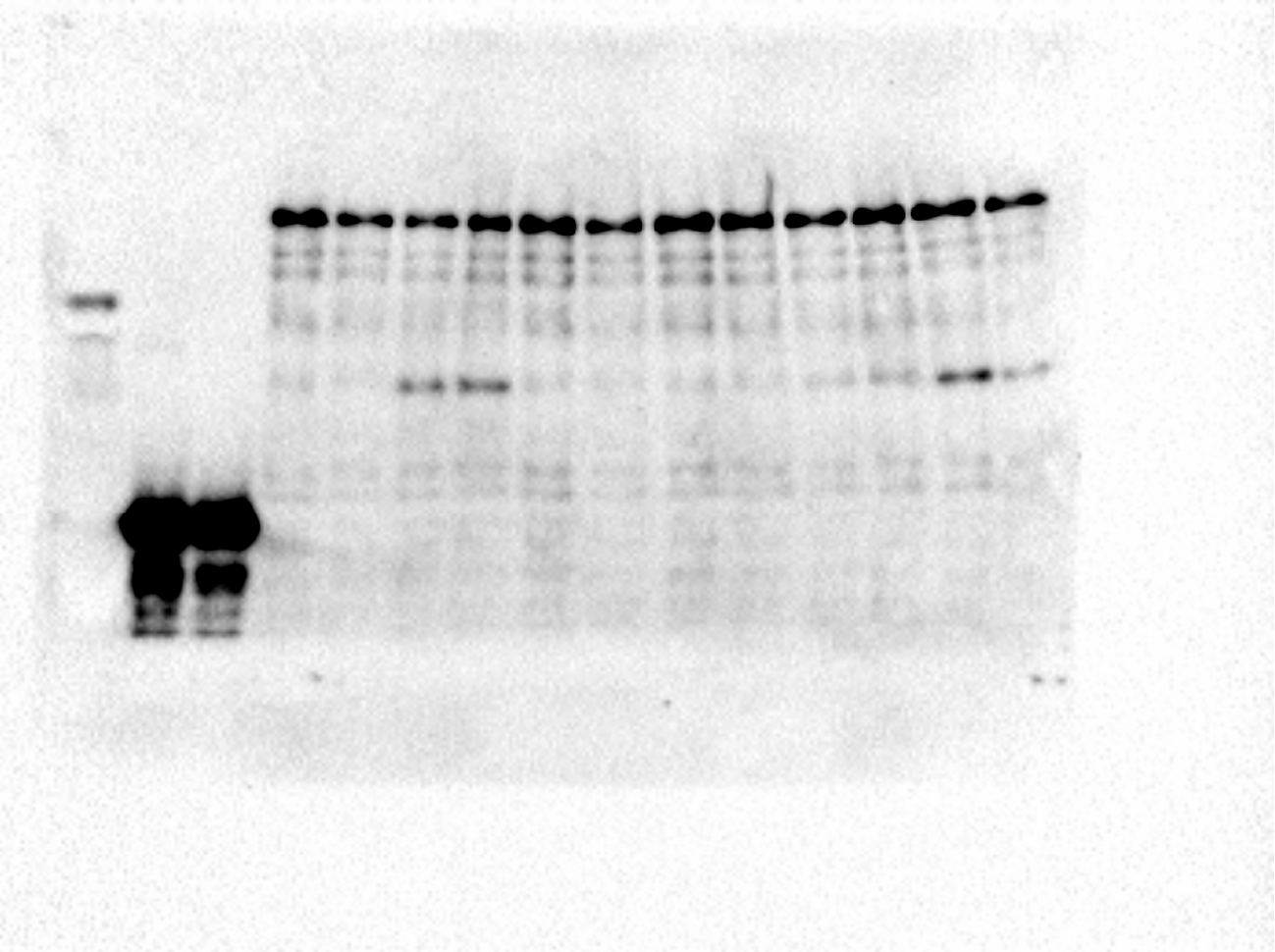

Supplement: Figure 4—source data 2. [file elife-104233-fig4-data2.zip › Figure 4/Fig 4E/Input-anti-GFP.tif]

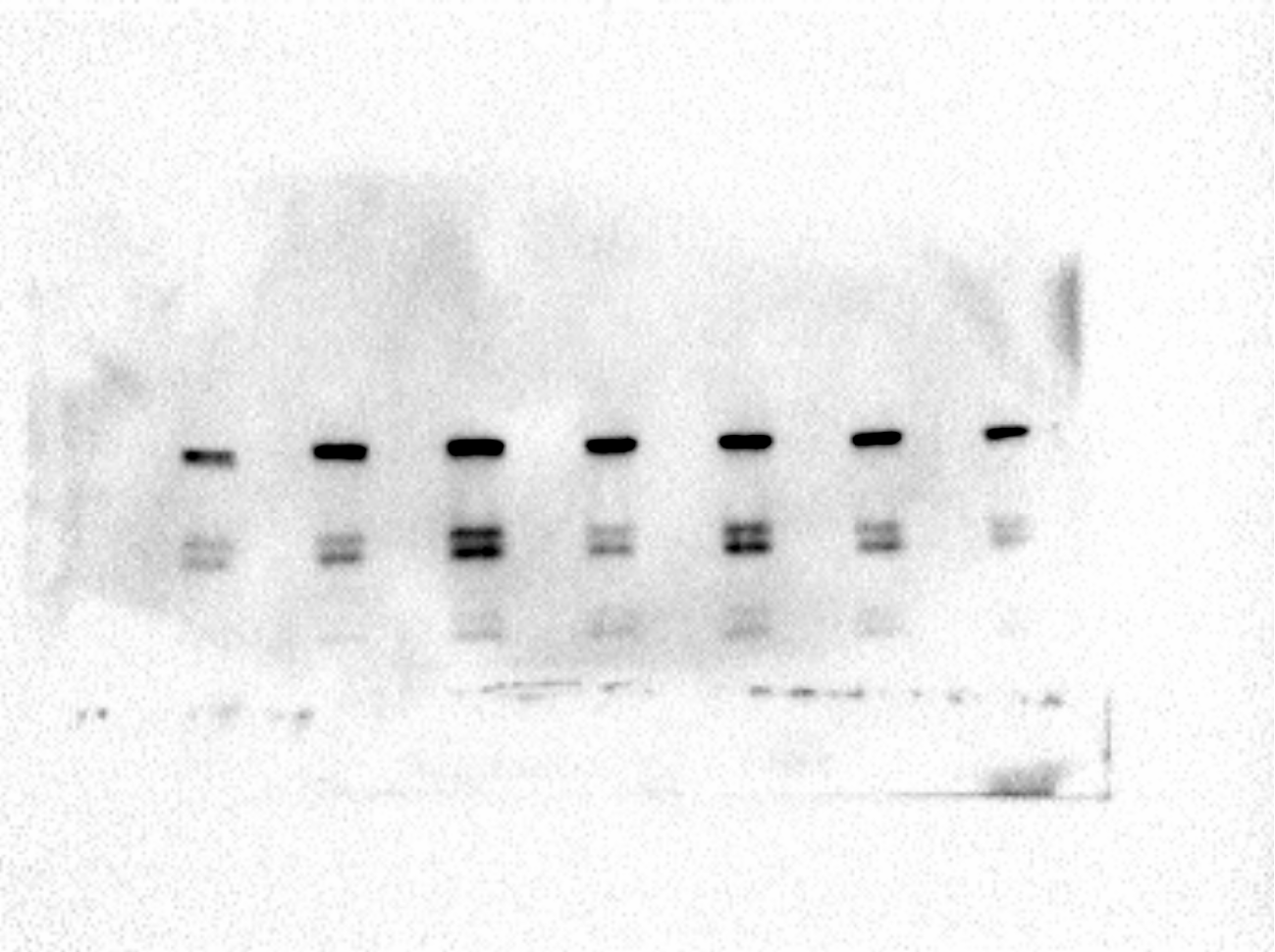

Supplement: Figure 4—source data 2. [file elife-104233-fig4-data2.zip › Figure 4/Fig 4E/Input-anti-Myc.tif]

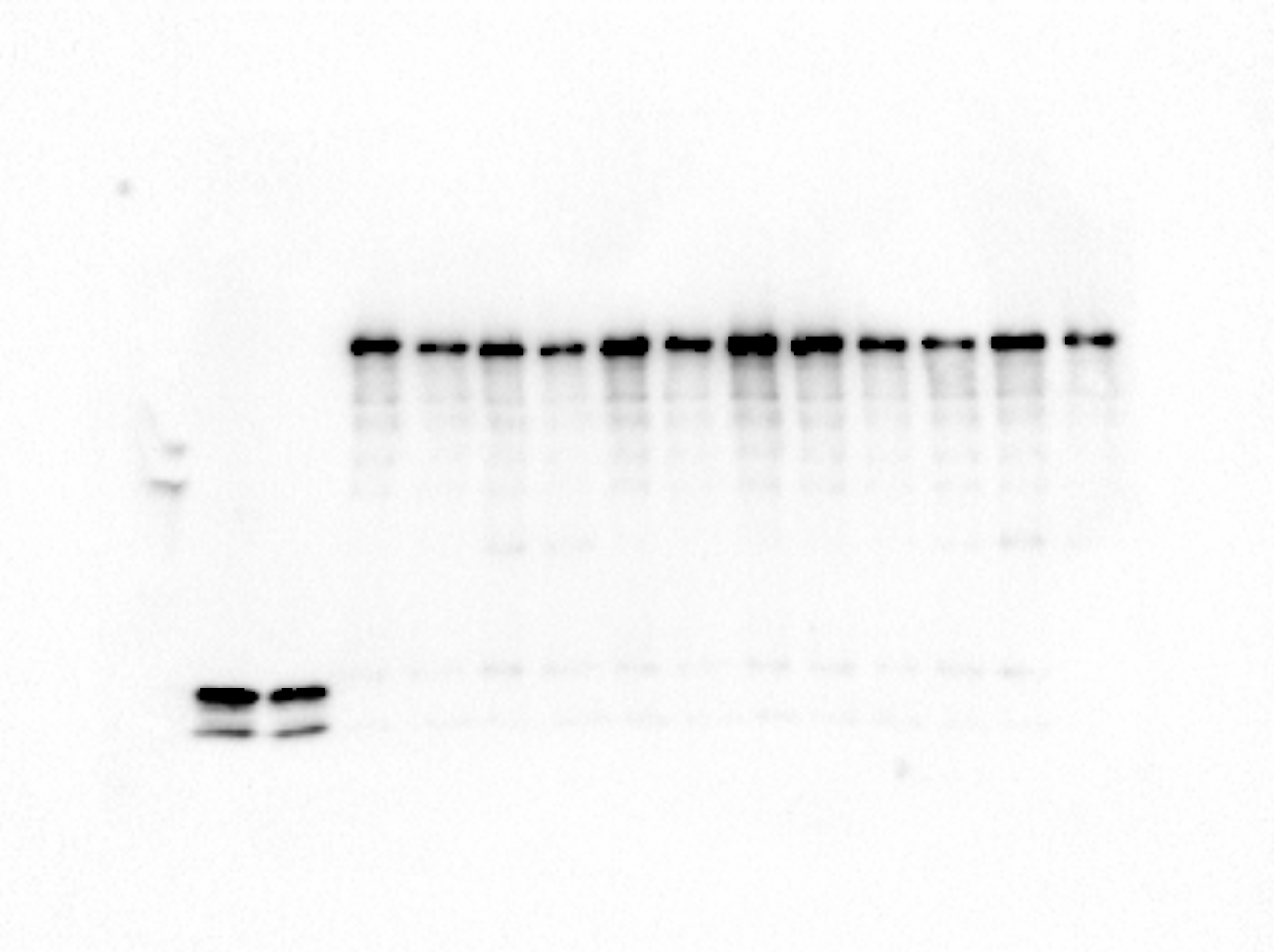

Supplement: Figure 4—source data 2. [file elife-104233-fig4-data2.zip › Figure 4/Fig 4E/Purif-anti-GFP.tif]

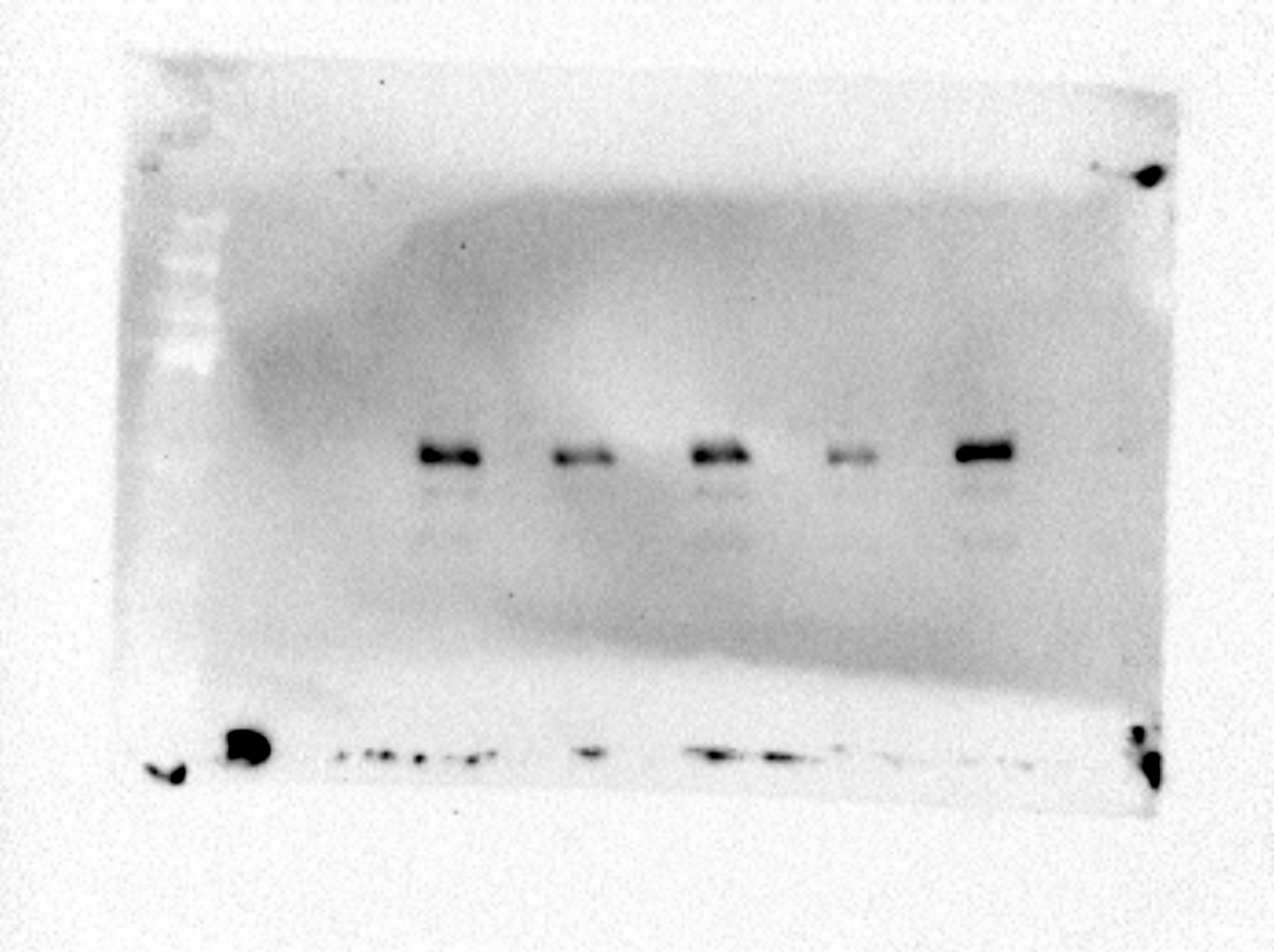

Supplement: Figure 4—source data 2. [file elife-104233-fig4-data2.zip › Figure 4/Fig 4E/Purif-anti-Myc.tif]

WCL

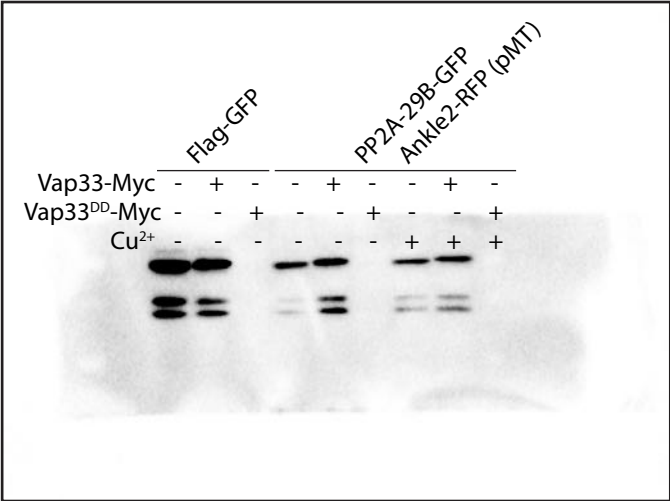

GFP purification

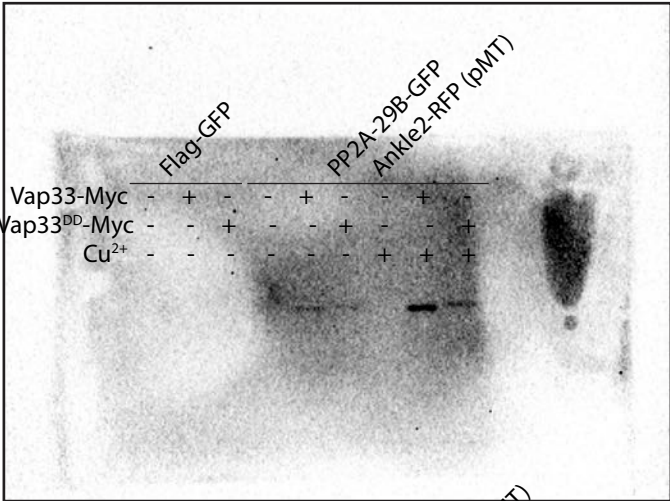

α-Myc

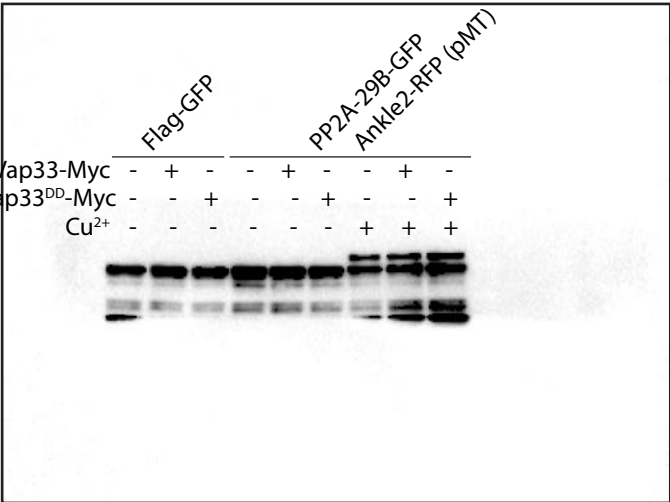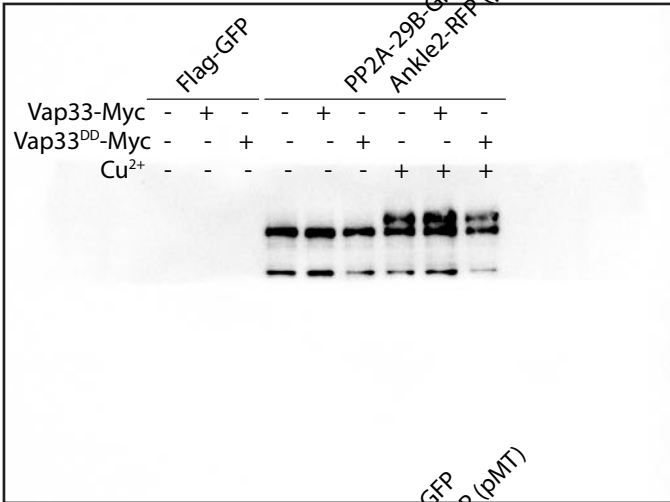

α-Ankle2

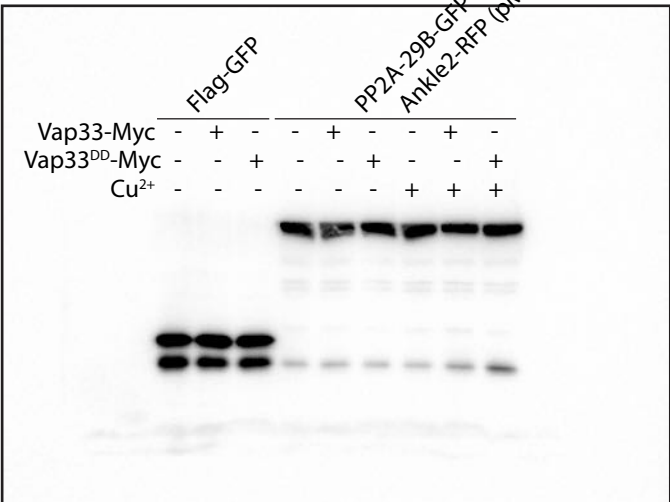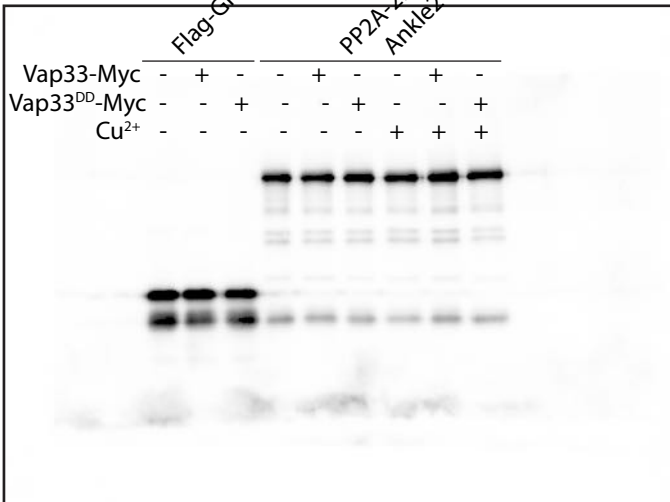

α-GFP

Supplement: Figure 4—figure supplement 1—source data 1. [file elife-104233-fig4-figsupp1-data1.zip › Figure 4 - figure supplement 1/Figure 4 - figure supplement 1D.pdf]

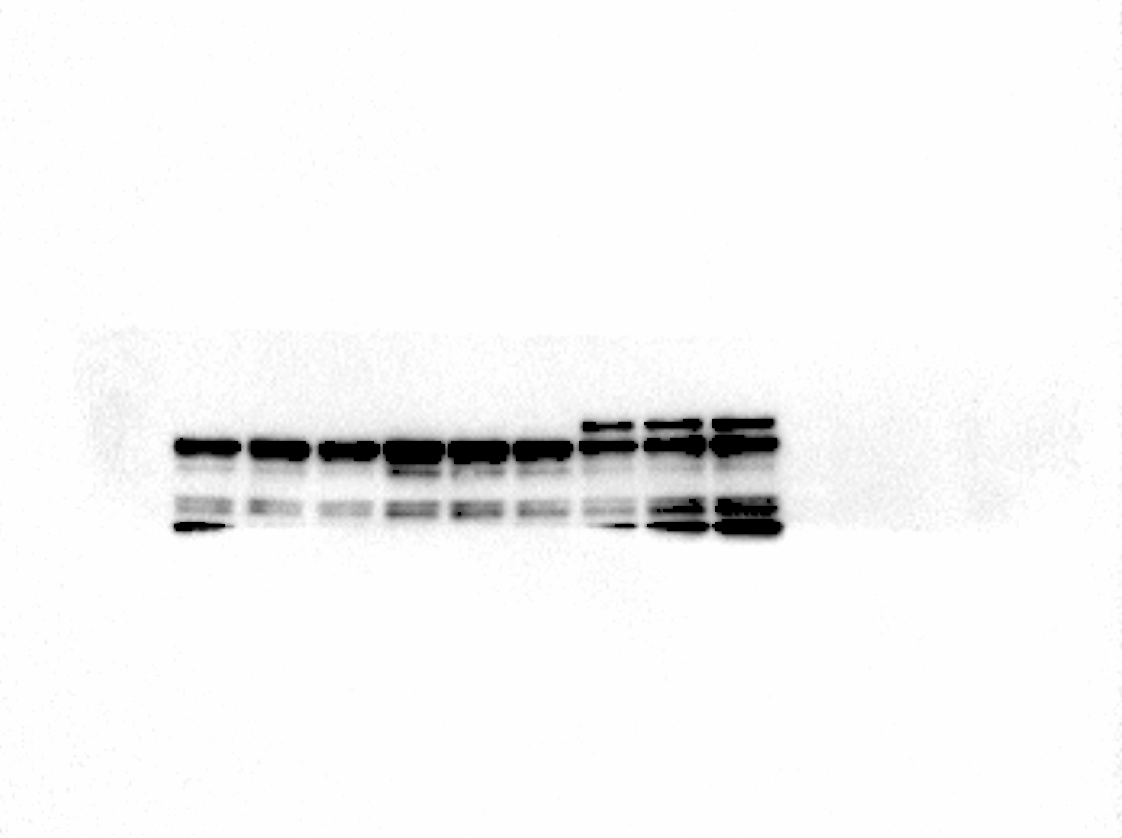

Supplement: Figure 4—figure supplement 1—source data 2. [file elife-104233-fig4-figsupp1-data2.zip › Figure 4 - figure supplement 1/Input, anti-Ankle2.tif]

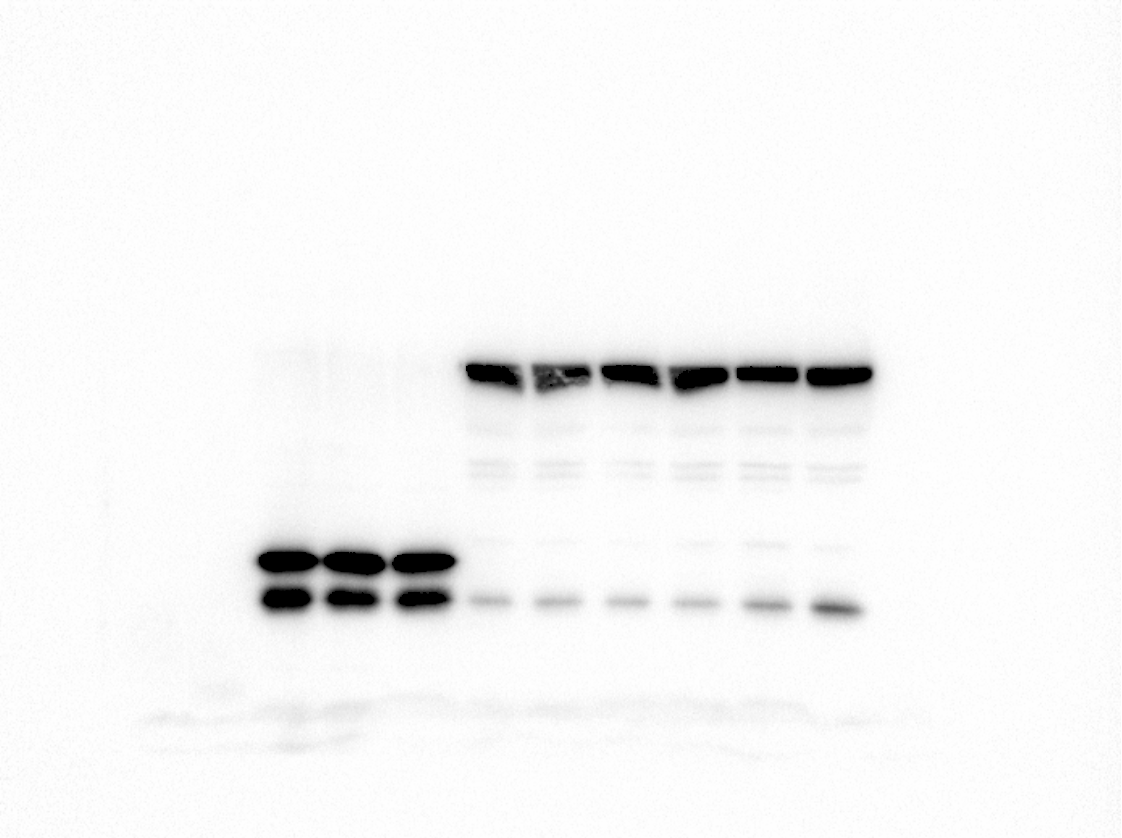

Supplement: Figure 4—figure supplement 1—source data 2. [file elife-104233-fig4-figsupp1-data2.zip › Figure 4 - figure supplement 1/Input, anti-GFP.tif]

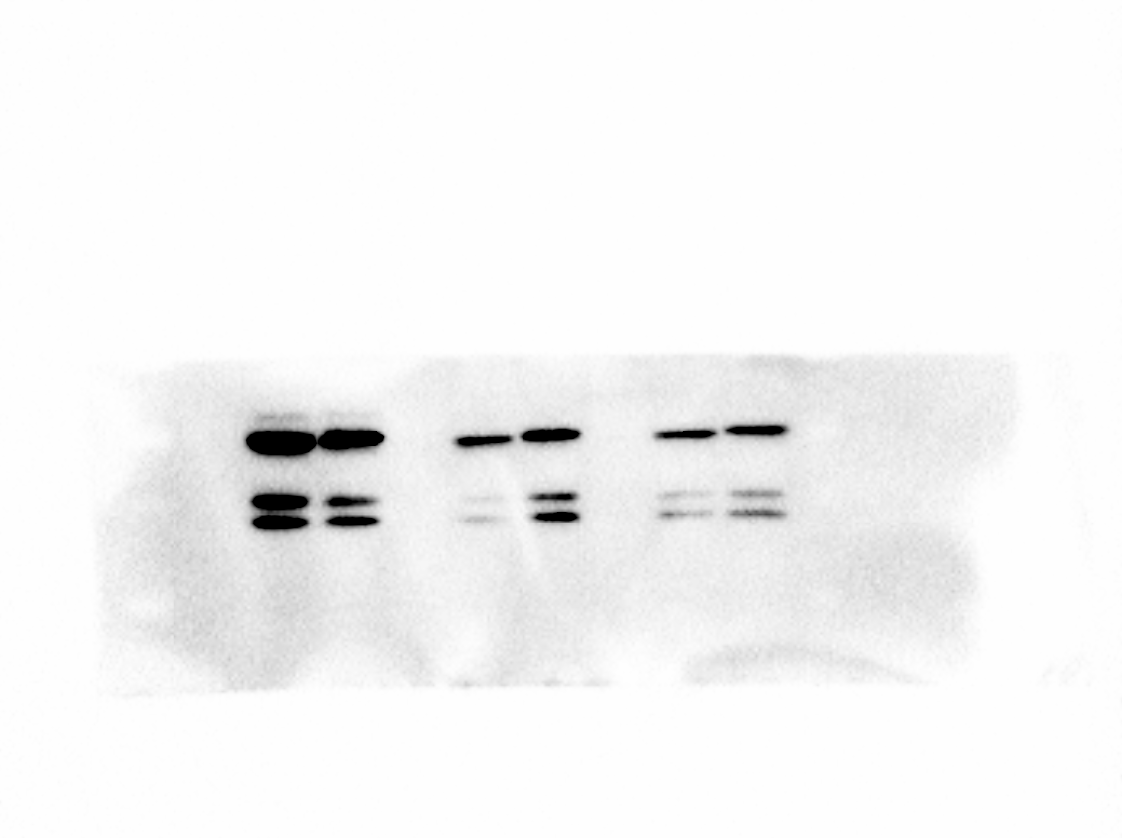

Supplement: Figure 4—figure supplement 1—source data 2. [file elife-104233-fig4-figsupp1-data2.zip › Figure 4 - figure supplement 1/Input, anti-Myc.tif]

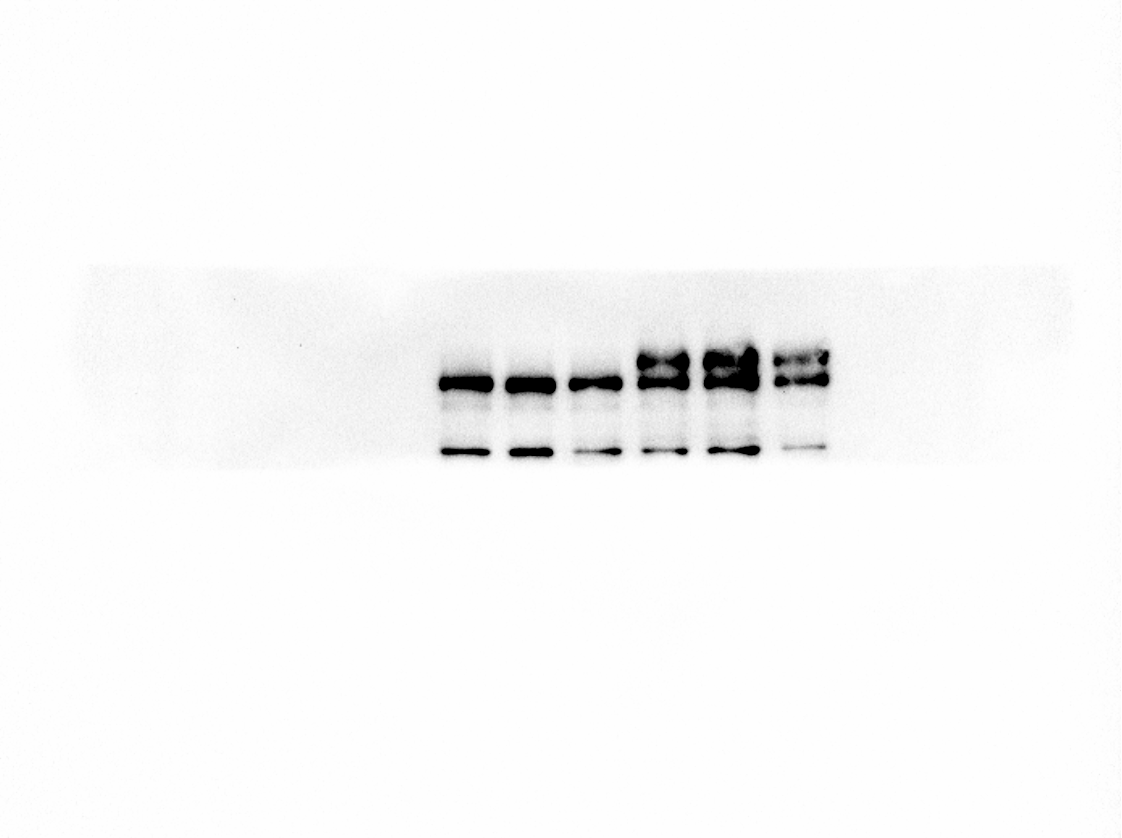

Supplement: Figure 4—figure supplement 1—source data 2. [file elife-104233-fig4-figsupp1-data2.zip › Figure 4 - figure supplement 1/Purif, anti-Ankle2.tif]

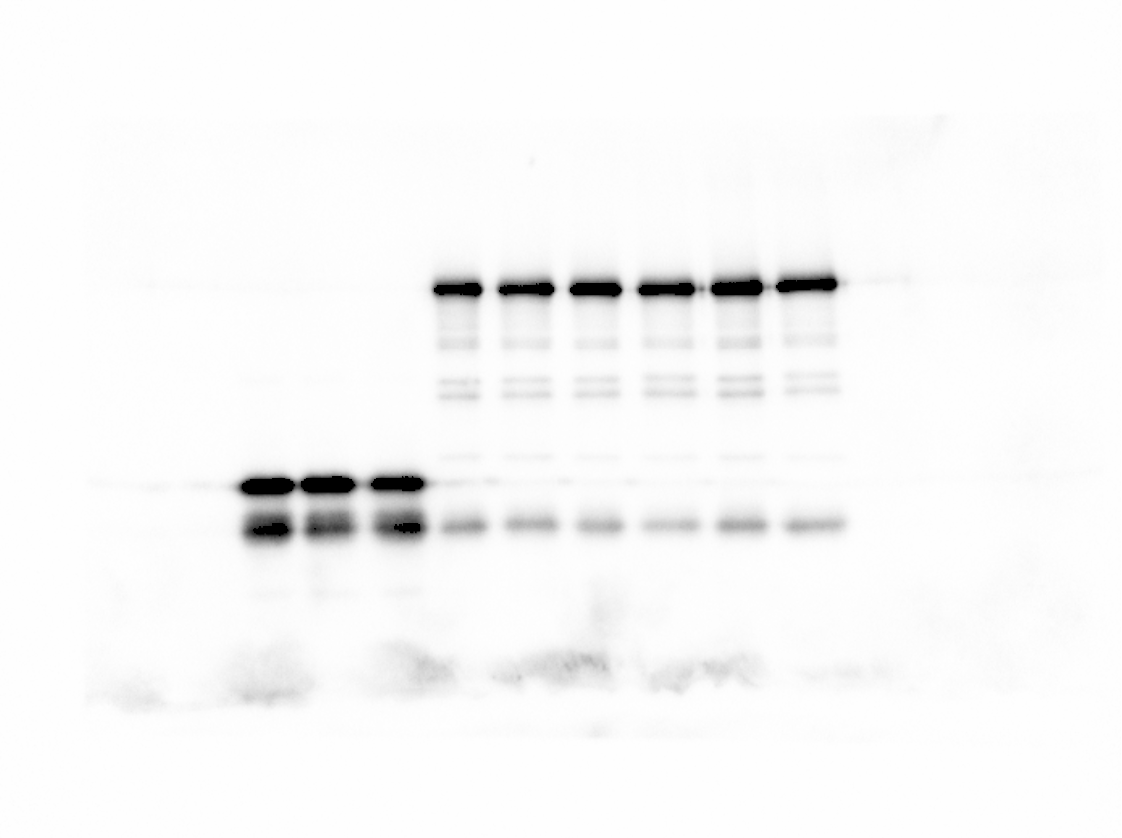

Supplement: Figure 4—figure supplement 1—source data 2. [file elife-104233-fig4-figsupp1-data2.zip › Figure 4 - figure supplement 1/Purif, anti-GFP.tif]

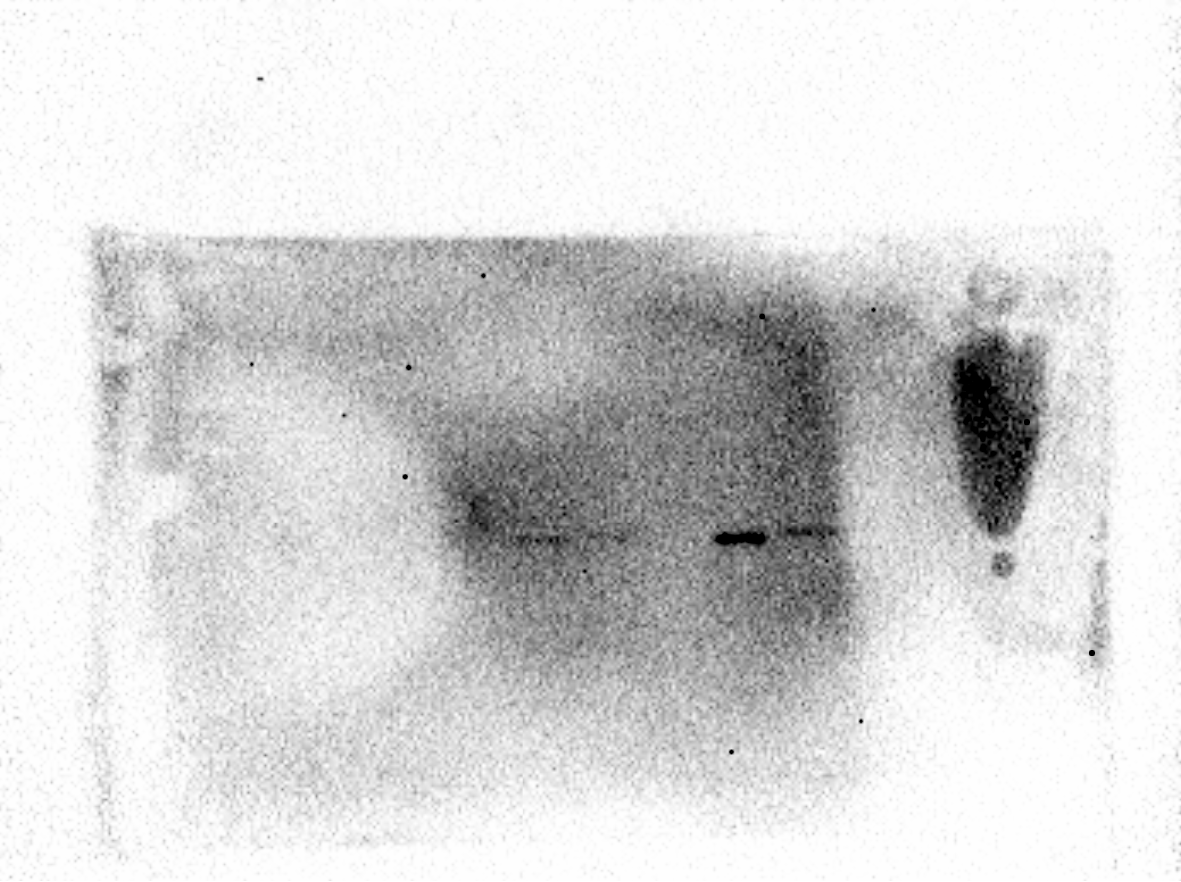

Supplement: Figure 4—figure supplement 1—source data 2. [file elife-104233-fig4-figsupp1-data2.zip › Figure 4 - figure supplement 1/Purif,anti-myc.tif]

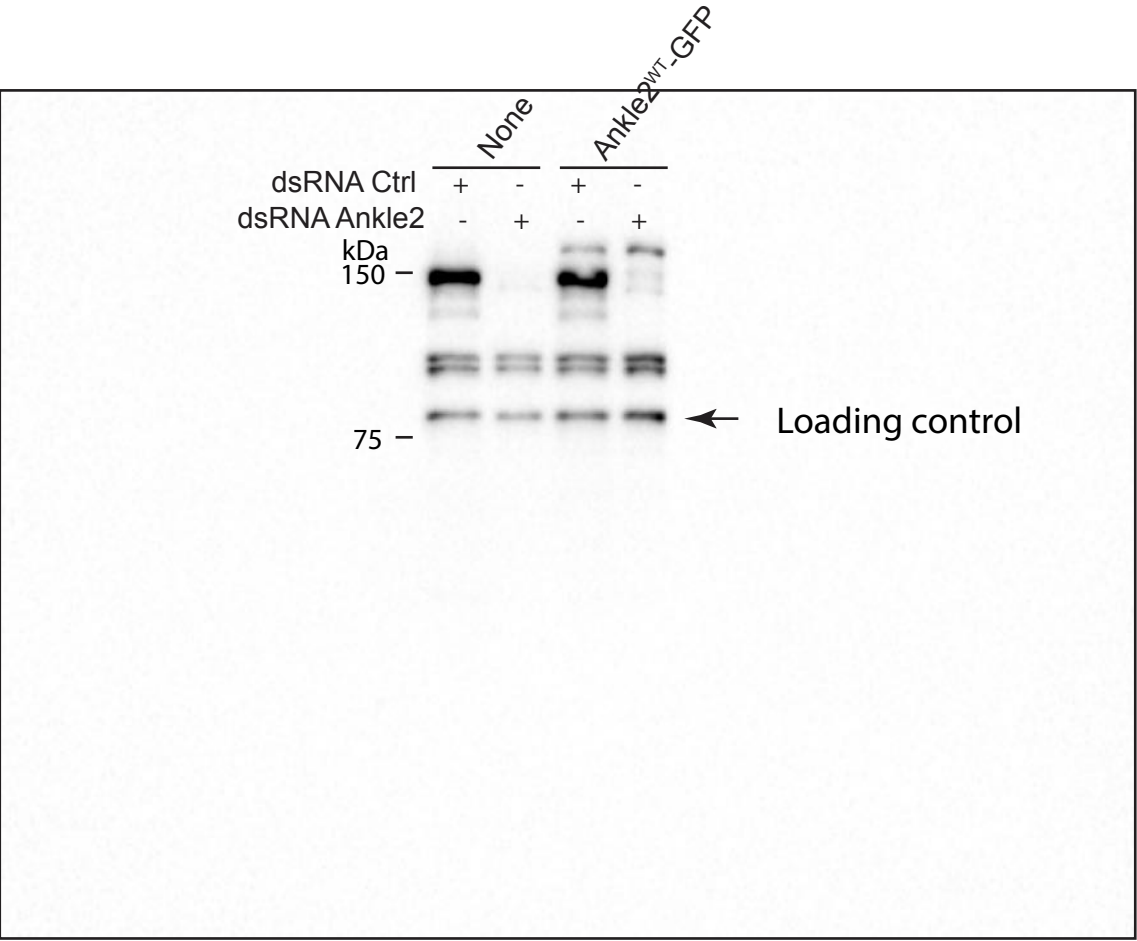

$\alpha$ -Ankle2

Supplement: Figure 5—source data 2. [file elife-104233-fig5-data2.zip › Figure 5/Figure 5A.pdf]

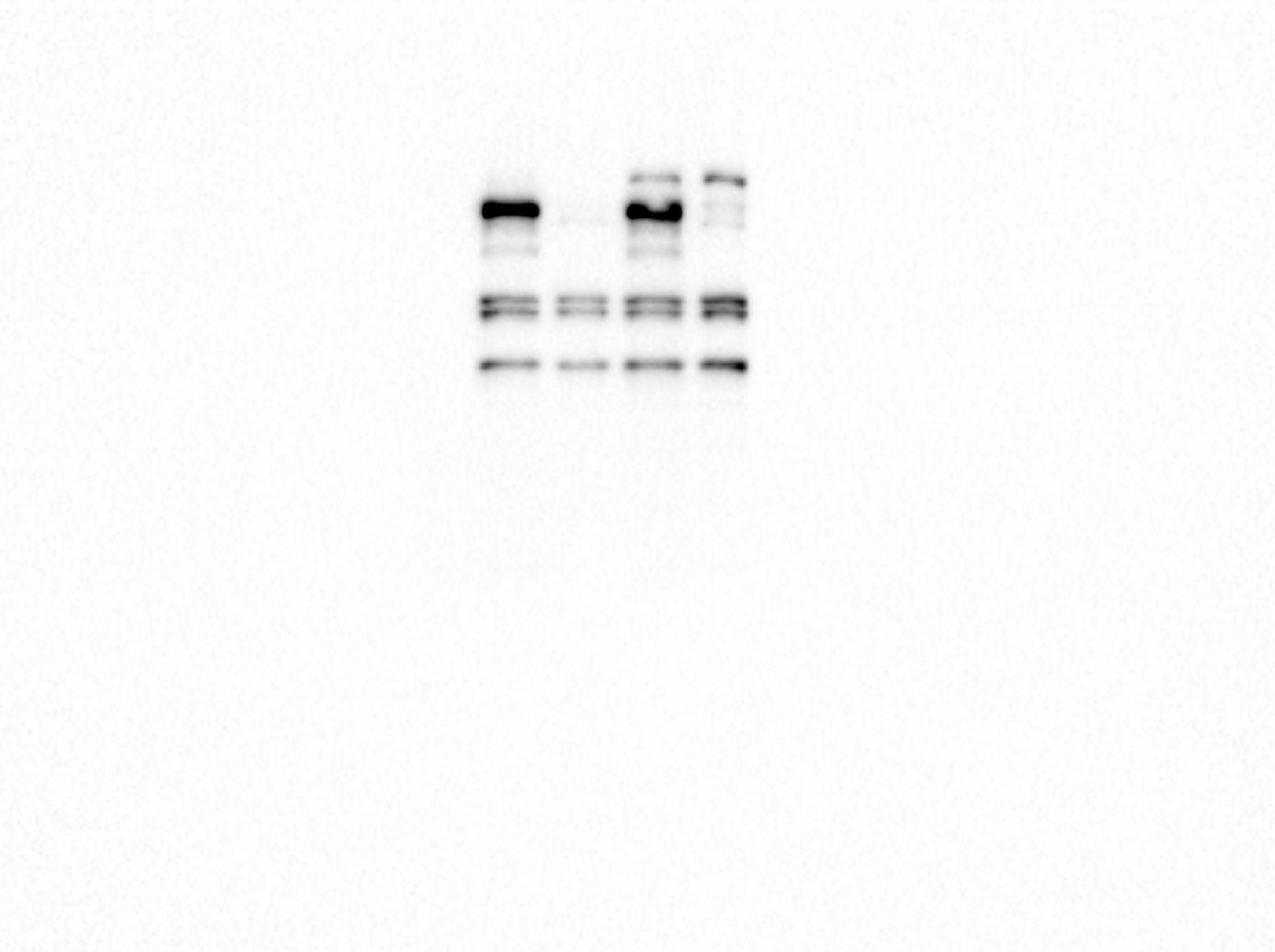

Supplement: Figure 5—source data 3. [file elife-104233-fig5-data3.zip › Figure 5/Fig 5A/anti-Ankle2(top-Ankle2,bottom-loading control).tif]

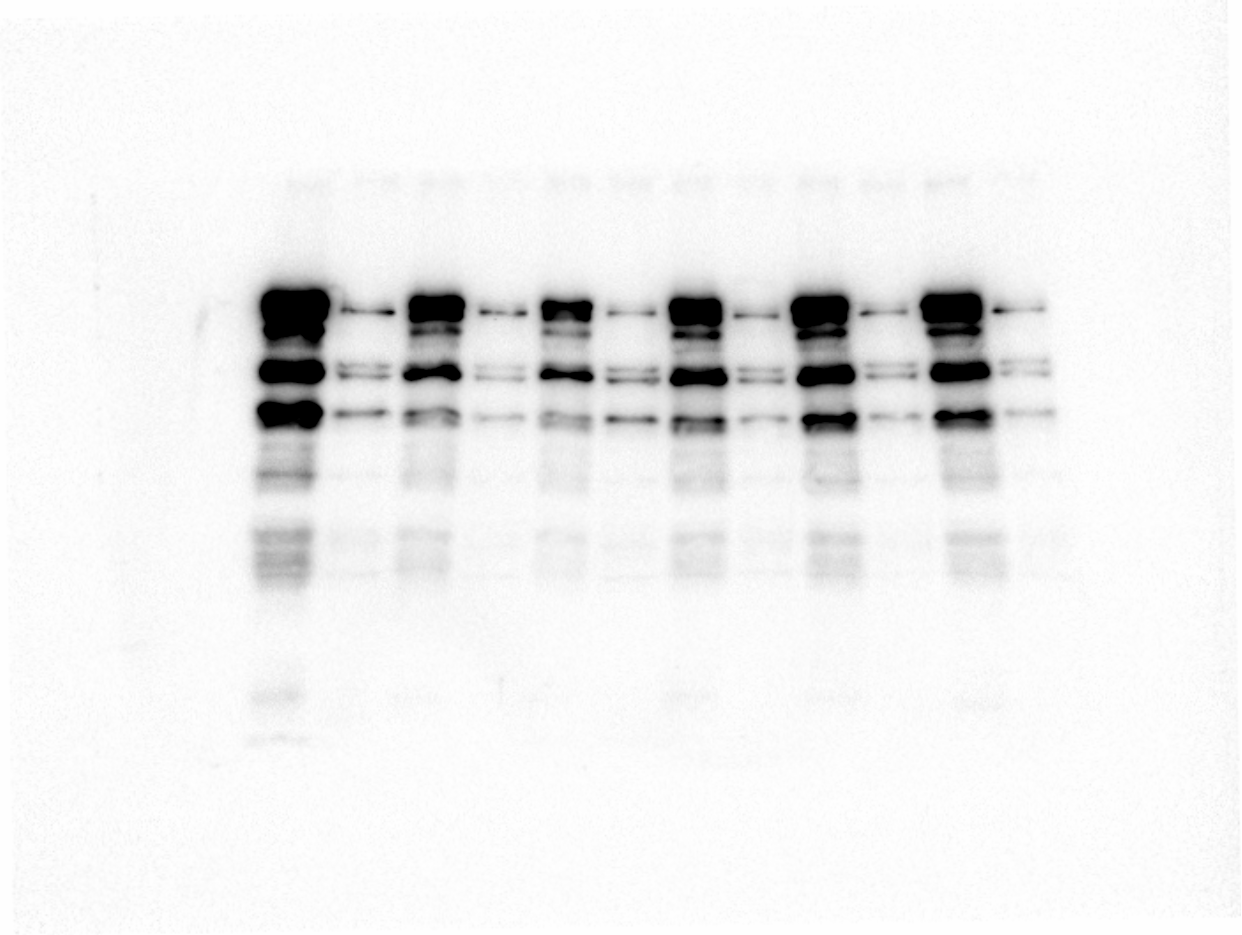

Supplement: Figure 5—source data 3. [file elife-104233-fig5-data3.zip › Figure 5/Fig 5D/anti-Ankle2.tif]

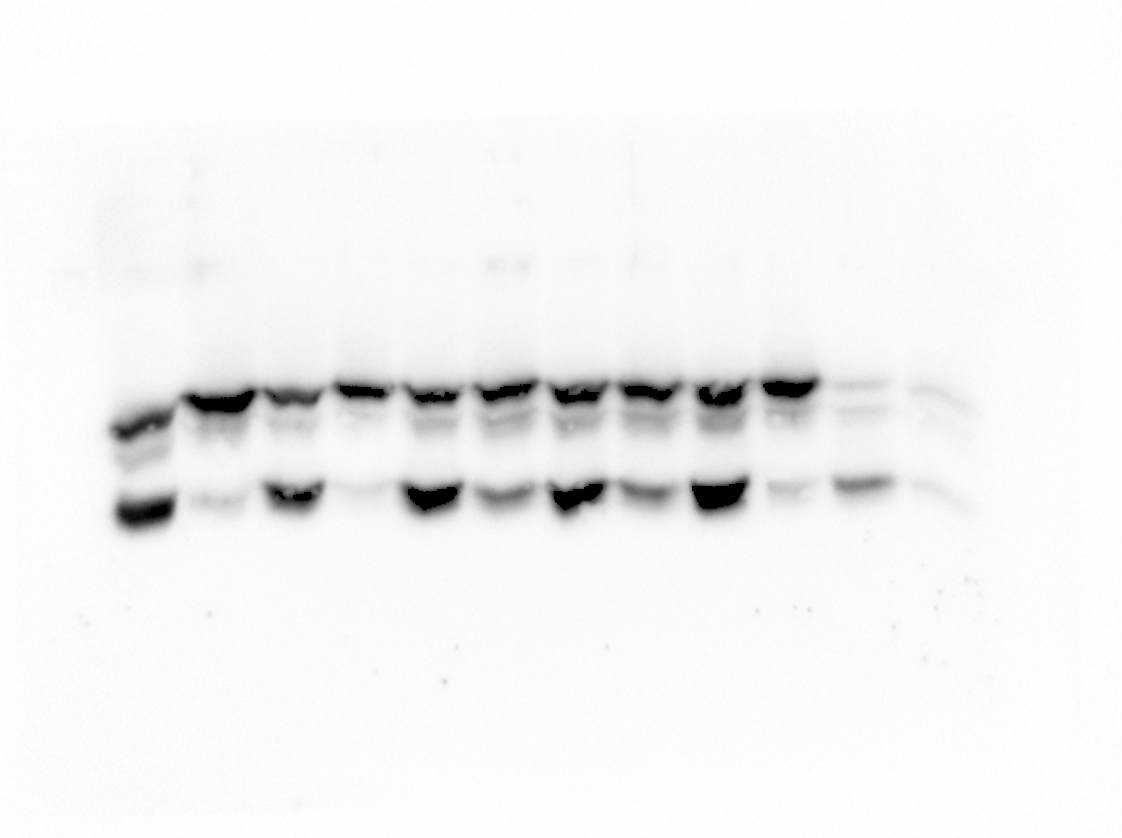

Supplement: Figure 5—source data 3. [file elife-104233-fig5-data3.zip › Figure 5/Fig 5D/anti-BAF(phostag).tif]

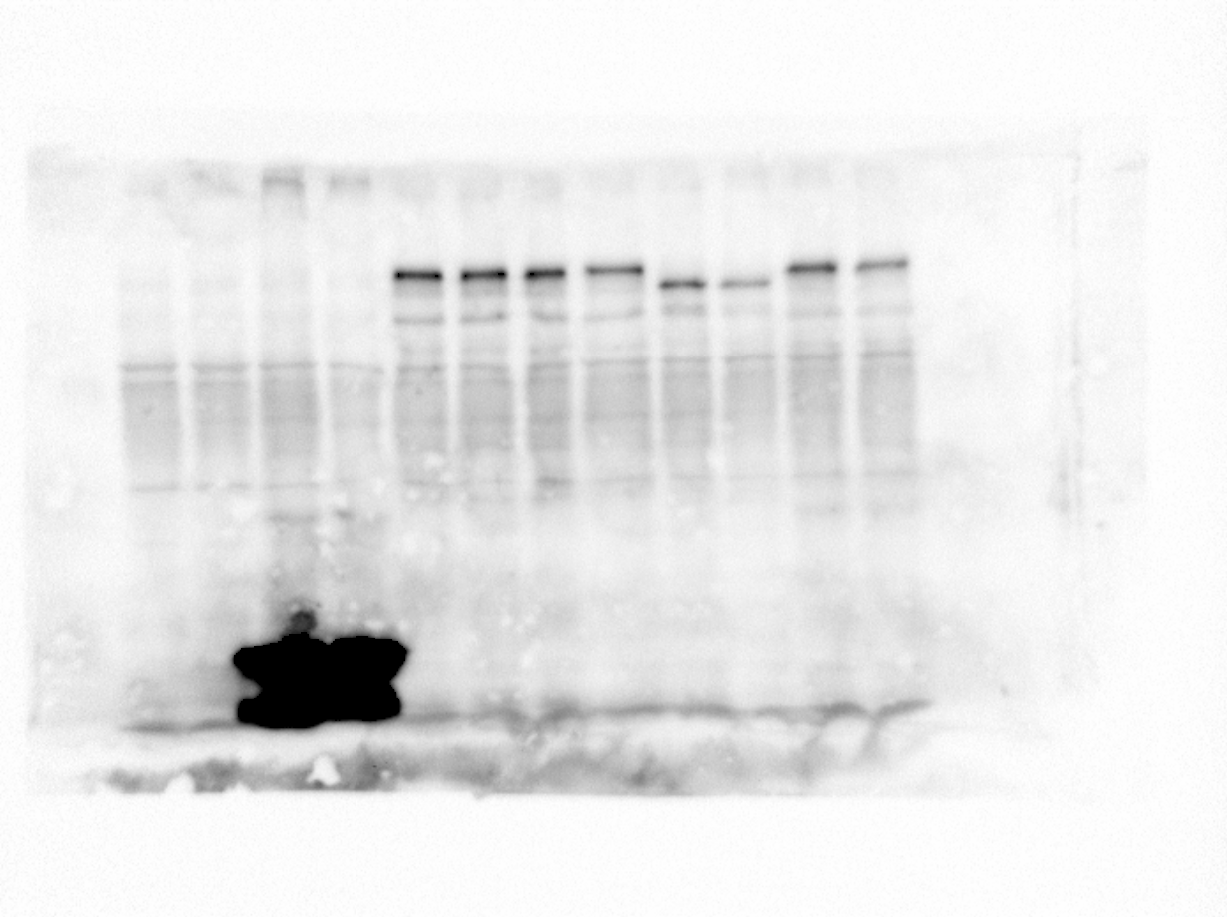

Supplement: Figure 5—source data 3. [file elife-104233-fig5-data3.zip › Figure 5/Fig 5D/anti-GFP.tif]

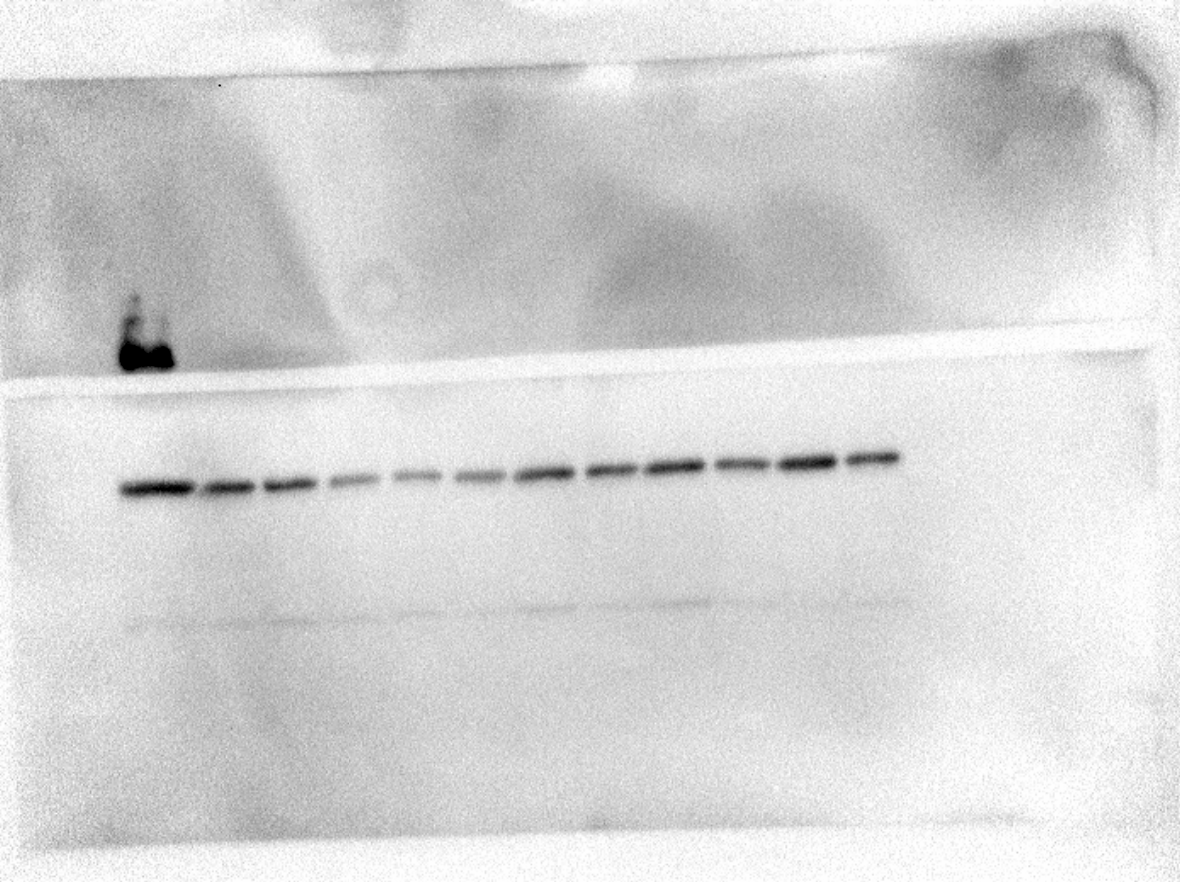

Supplement: Figure 5—source data 3. [file elife-104233-fig5-data3.zip › Figure 5/Fig 5D/anti-Tubulin.tif]

# $\alpha$ -GFP

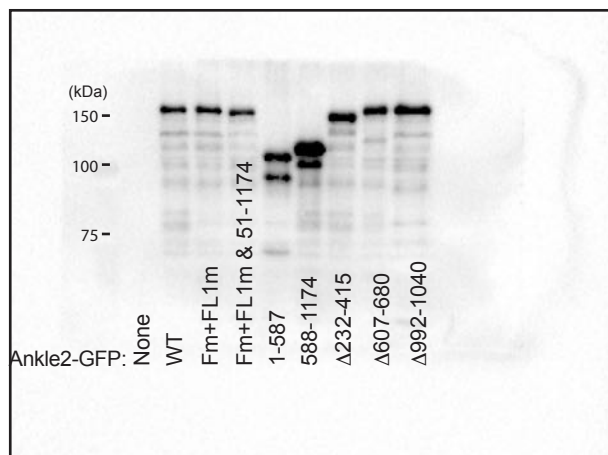

# $\alpha$ -Tubulin

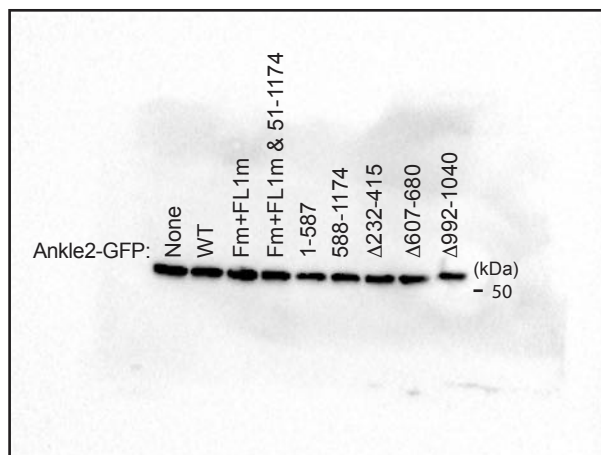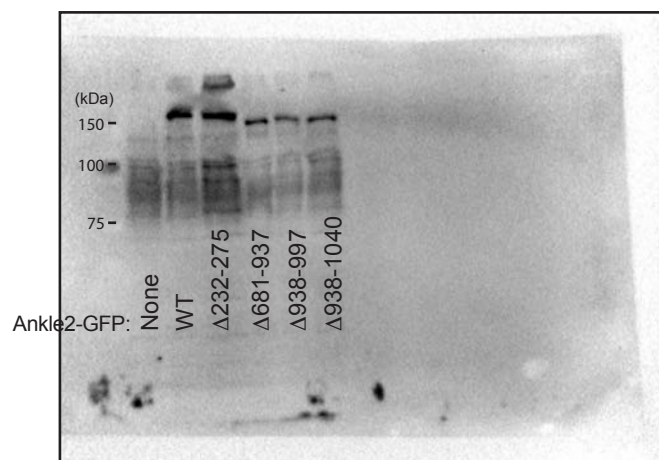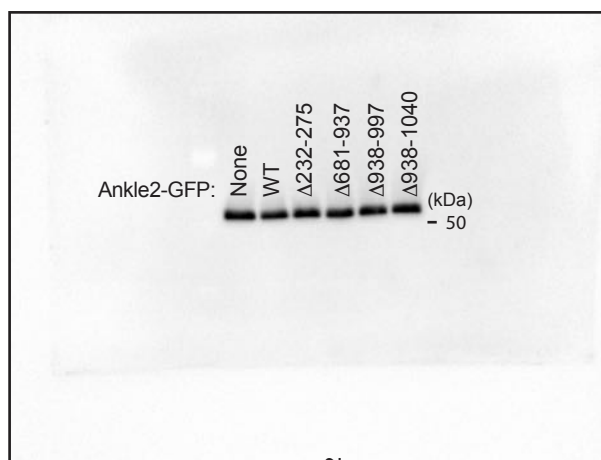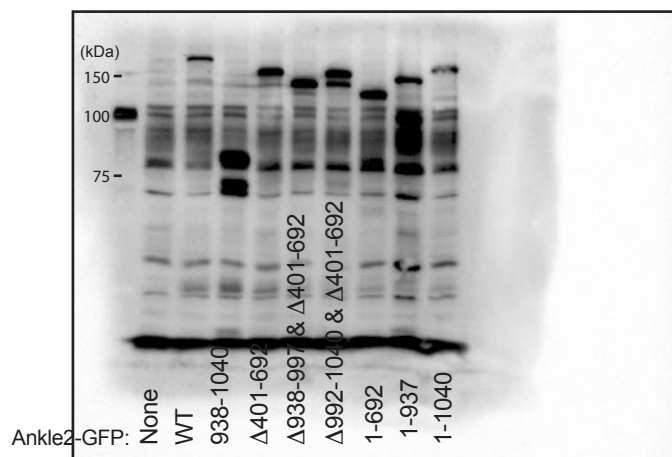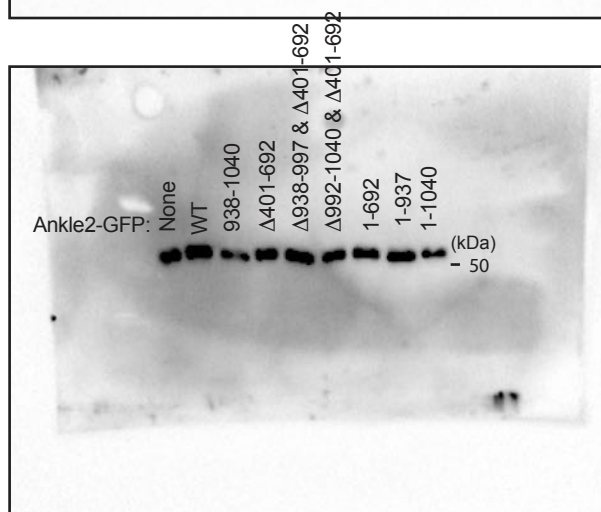

Supplement: Figure 5—figure supplement 1—source data 2. [file elife-104233-fig5-figsupp1-data2.zip › Figure 5 - figure supplement 1/Figure 5 - figure supplement 1A.pdf]

phostag+ $\alpha$ -BAF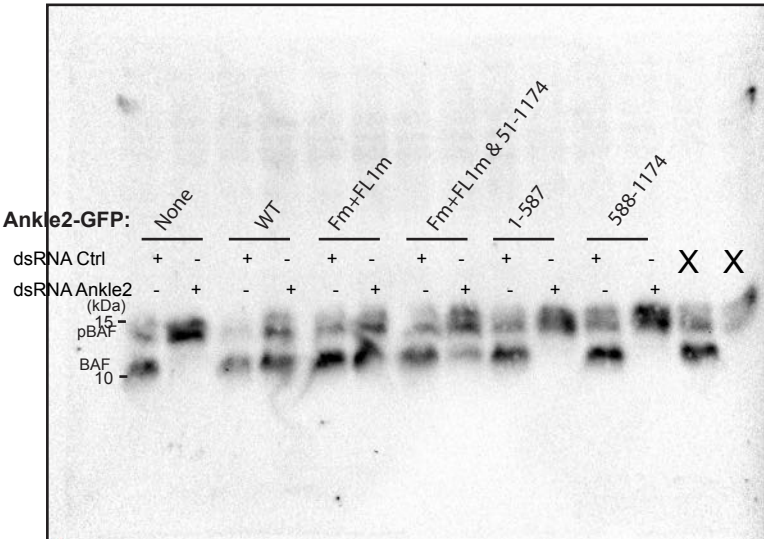phostag+ $\alpha$ -BAF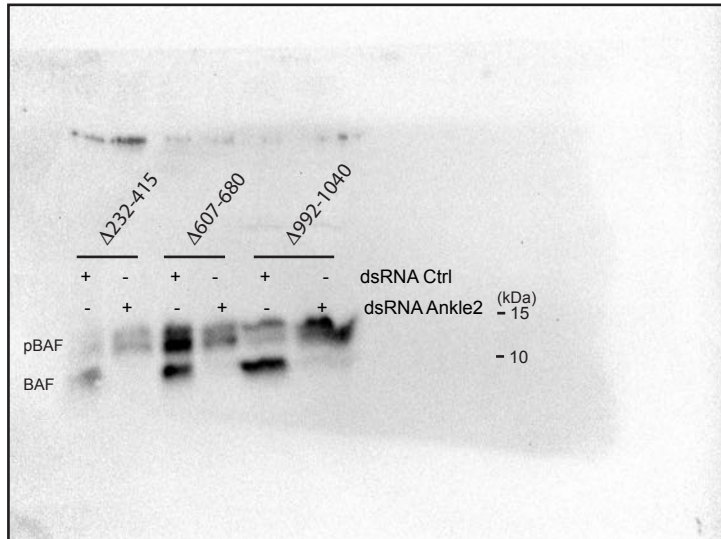phostag+ $\alpha$ -BAF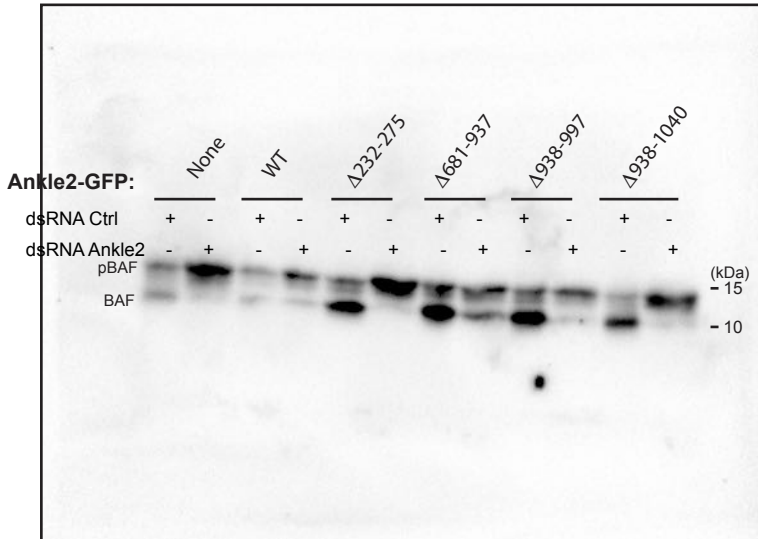phostag+ $\alpha$ -BAF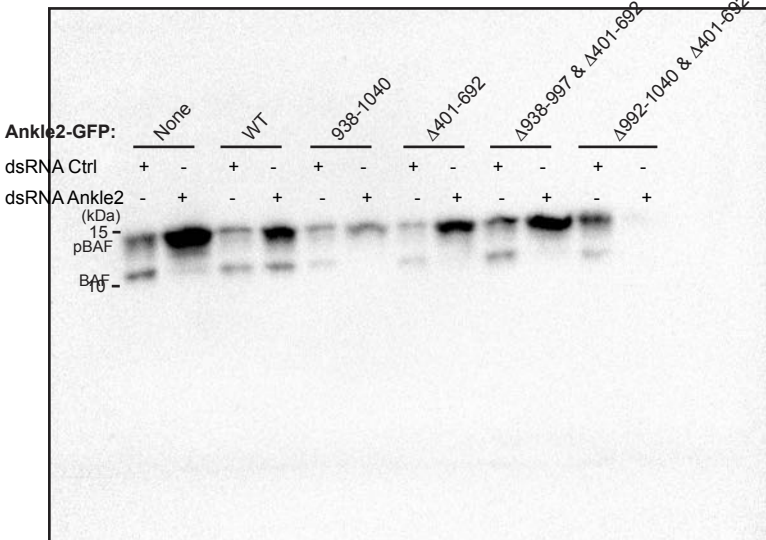phostag+ $\alpha$ -BAF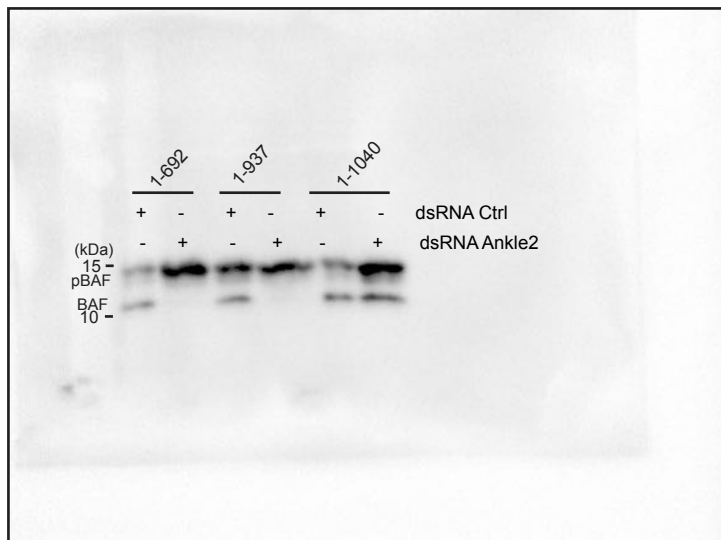

Supplement: Figure 5—figure supplement 1—source data 2. [file elife-104233-fig5-figsupp1-data2.zip › Figure 5 - figure supplement 1/Figure 5 - figure supplement 1B.pdf]

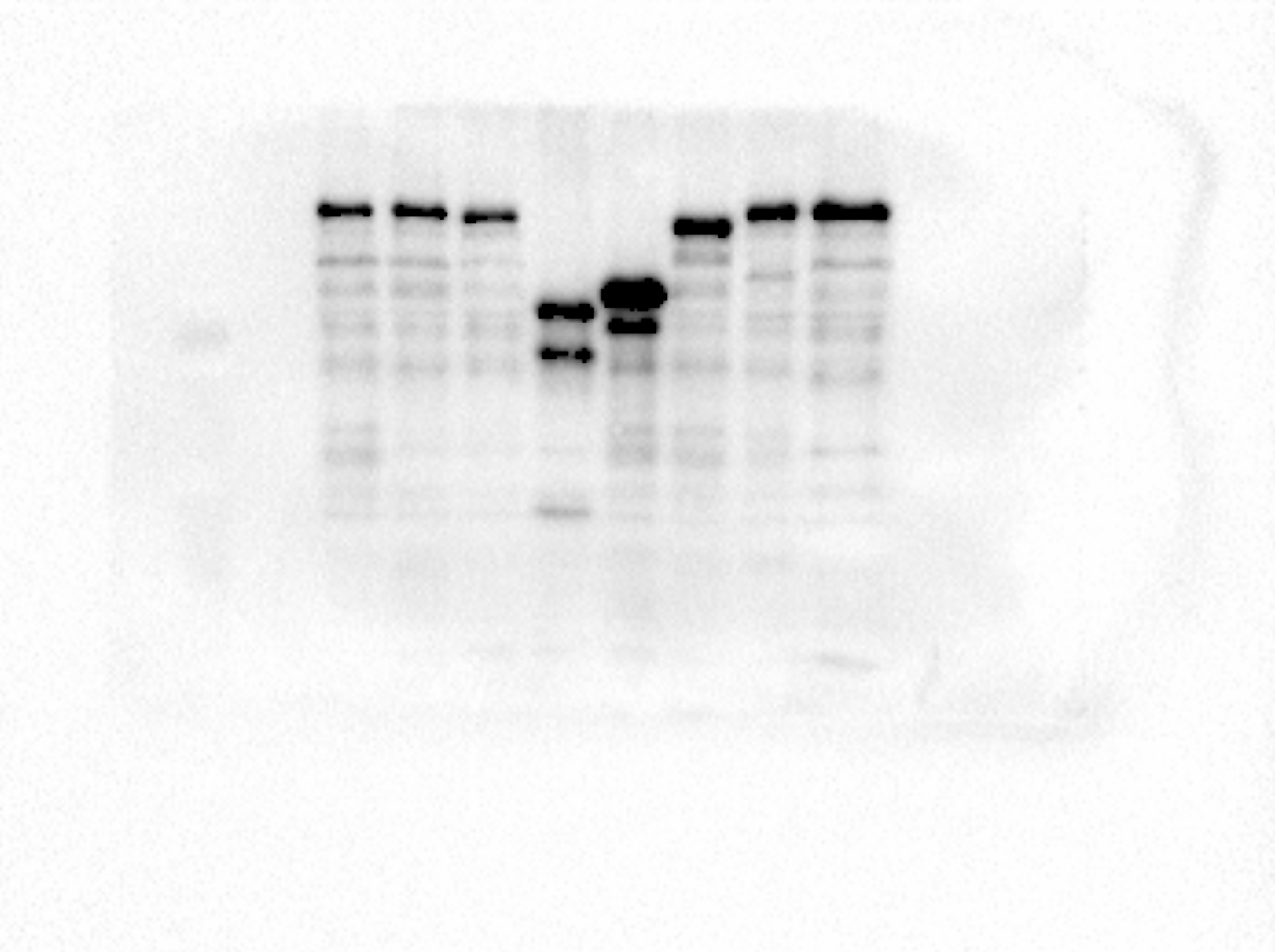

Supplement: Figure 5—figure supplement 1—source data 3. [file elife-104233-fig5-figsupp1-data3.zip › Figure 5 - figure supplement 1/anti-GFP (Left panel).tif]

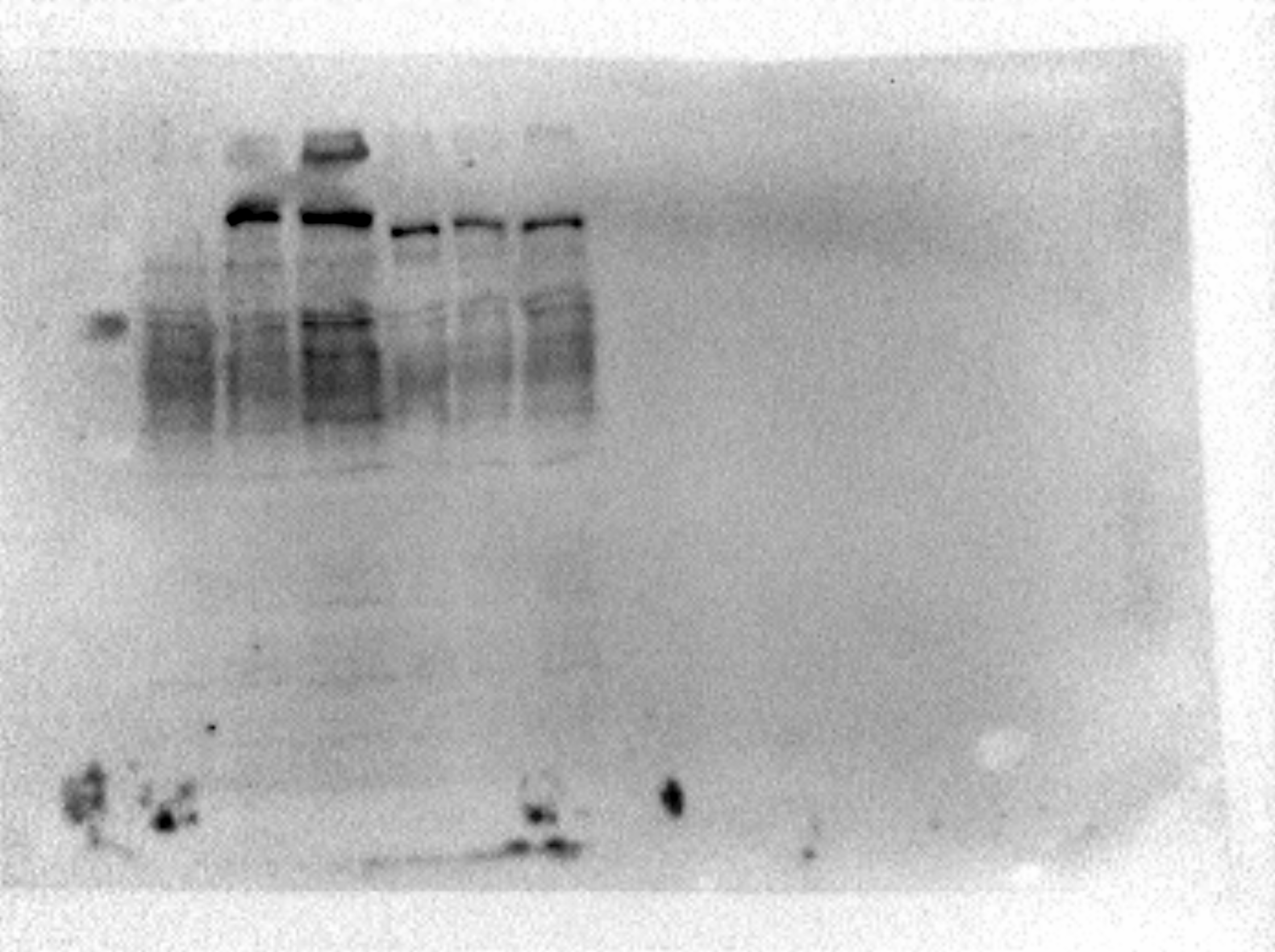

Supplement: Figure 5—figure supplement 1—source data 3. [file elife-104233-fig5-figsupp1-data3.zip › Figure 5 - figure supplement 1/anti-GFP (Middle panel).tif]

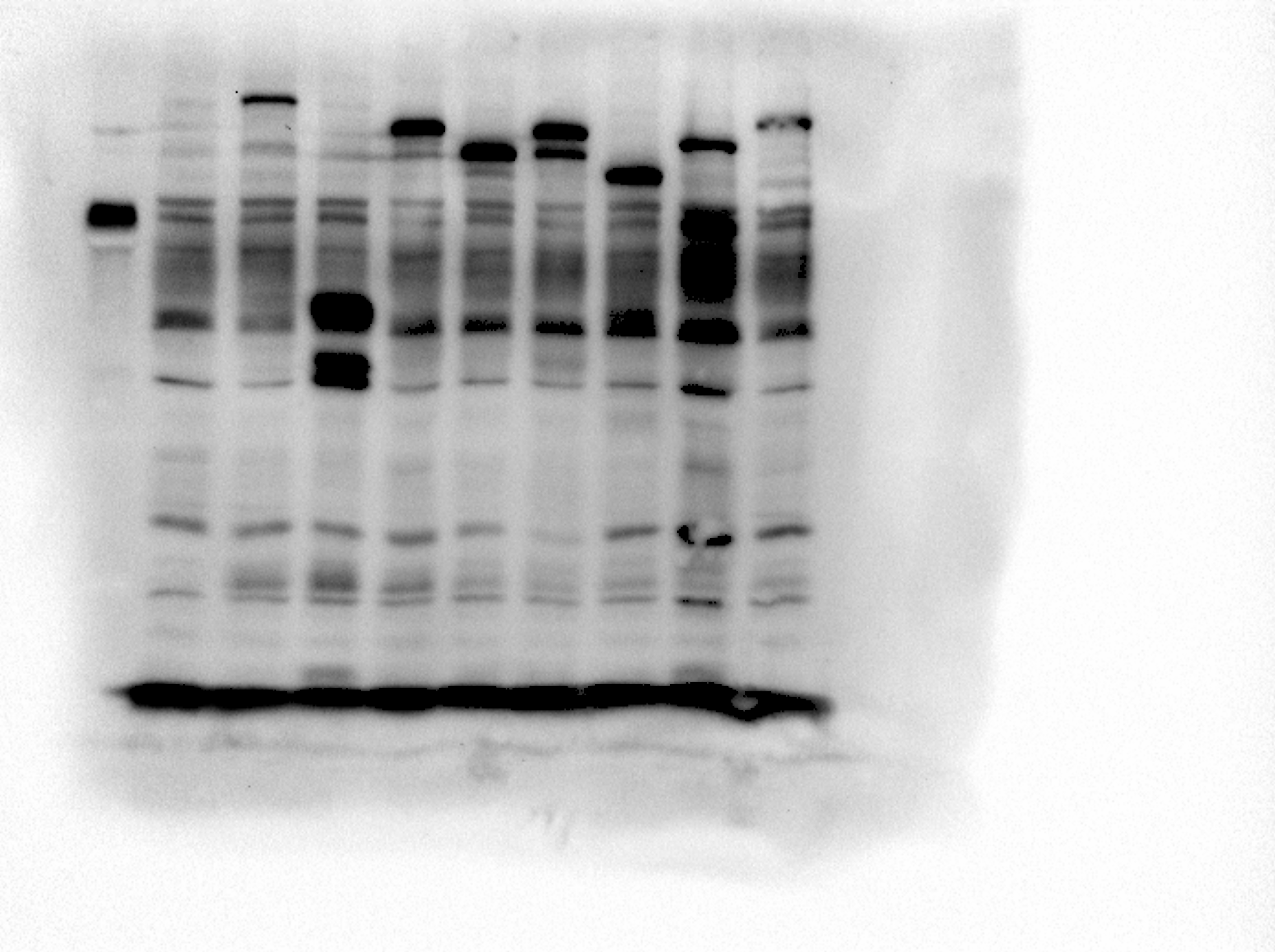

Supplement: Figure 5—figure supplement 1—source data 3. [file elife-104233-fig5-figsupp1-data3.zip › Figure 5 - figure supplement 1/anti-GFP (Right panel).tif]

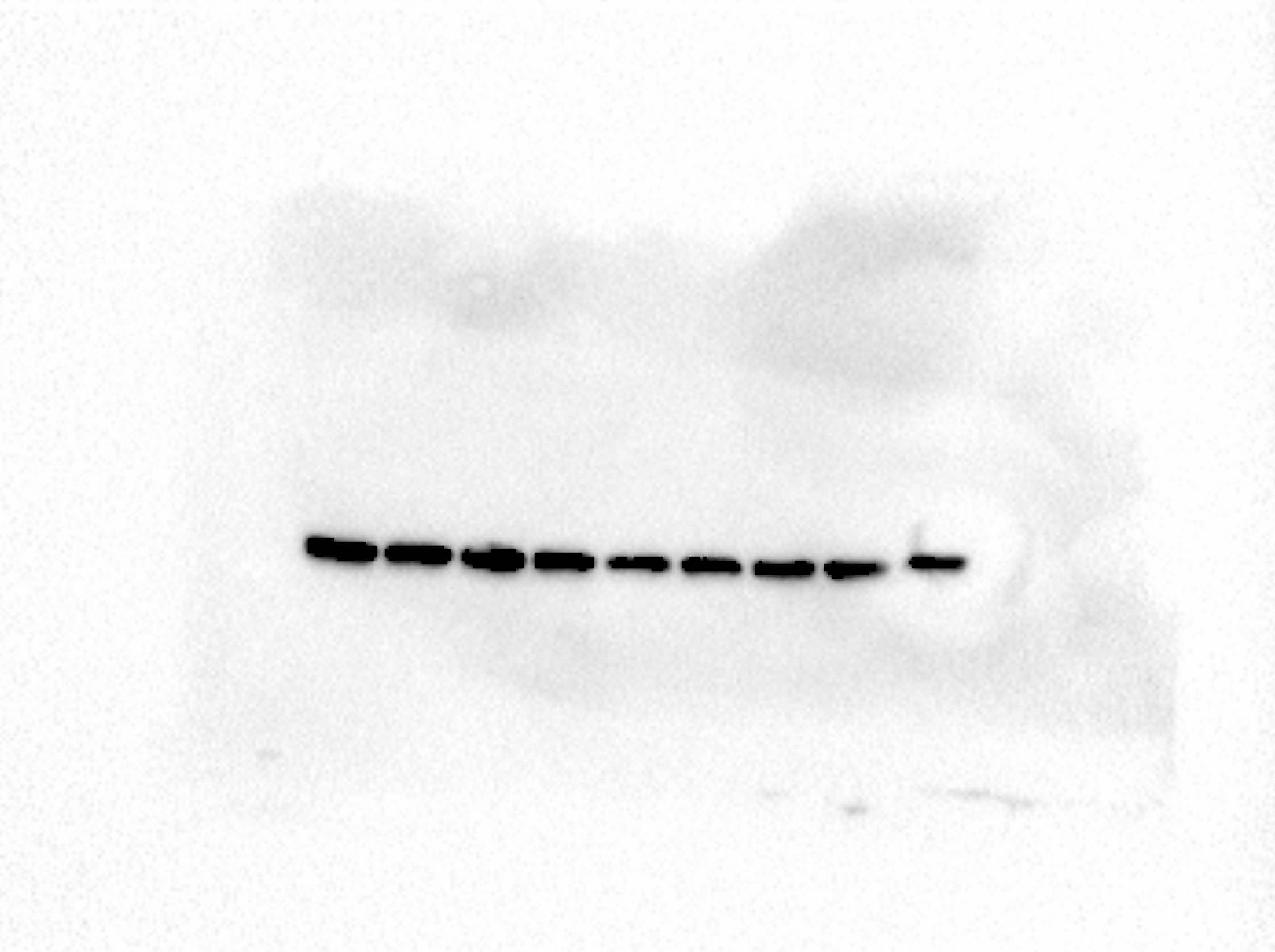

Supplement: Figure 5—figure supplement 1—source data 3. [file elife-104233-fig5-figsupp1-data3.zip › Figure 5 - figure supplement 1/anti-Tubulin (Left panel).tif]

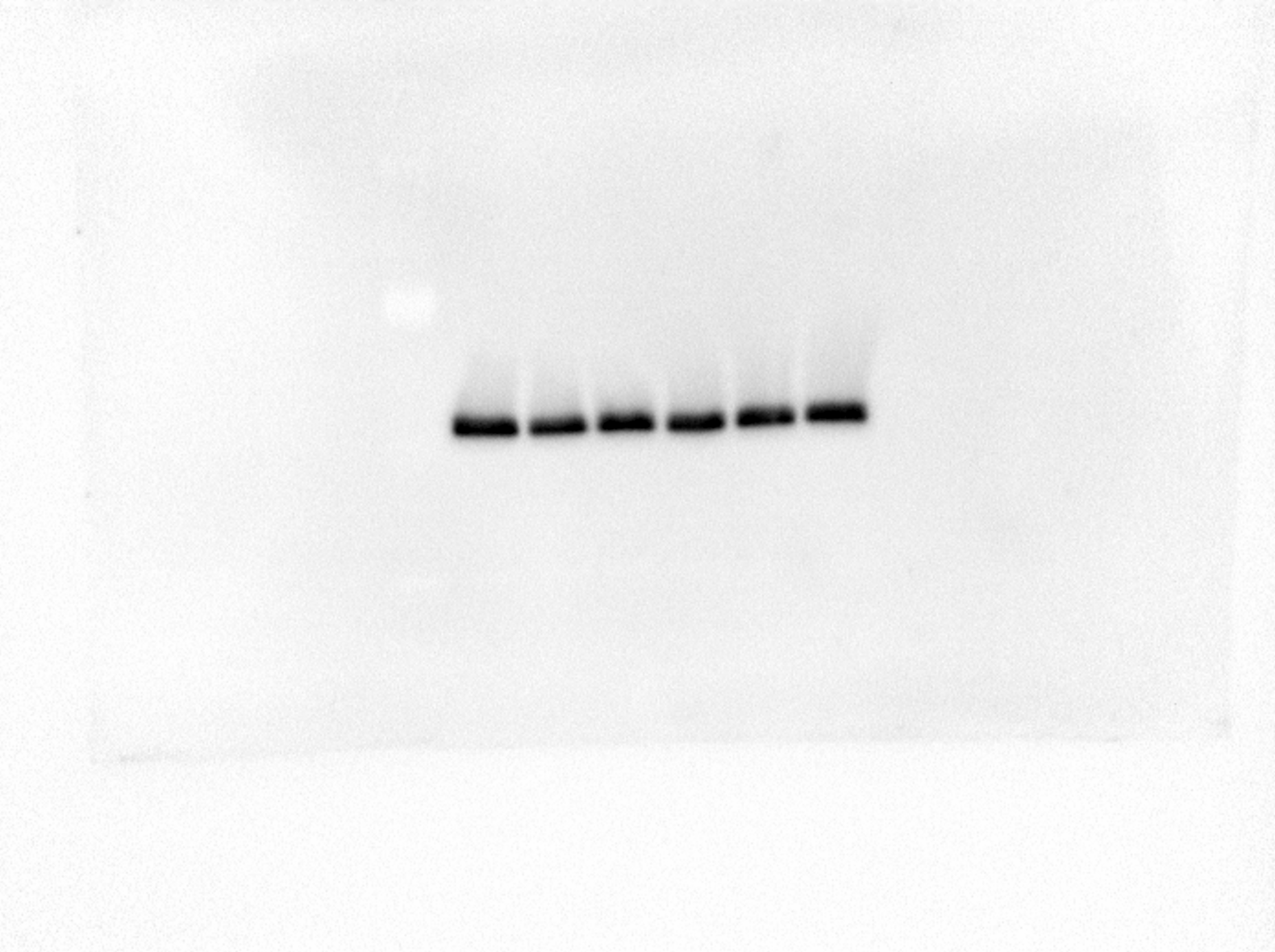

Supplement: Figure 5—figure supplement 1—source data 3. [file elife-104233-fig5-figsupp1-data3.zip › Figure 5 - figure supplement 1/anti-Tubulin (Middle panel).tif]

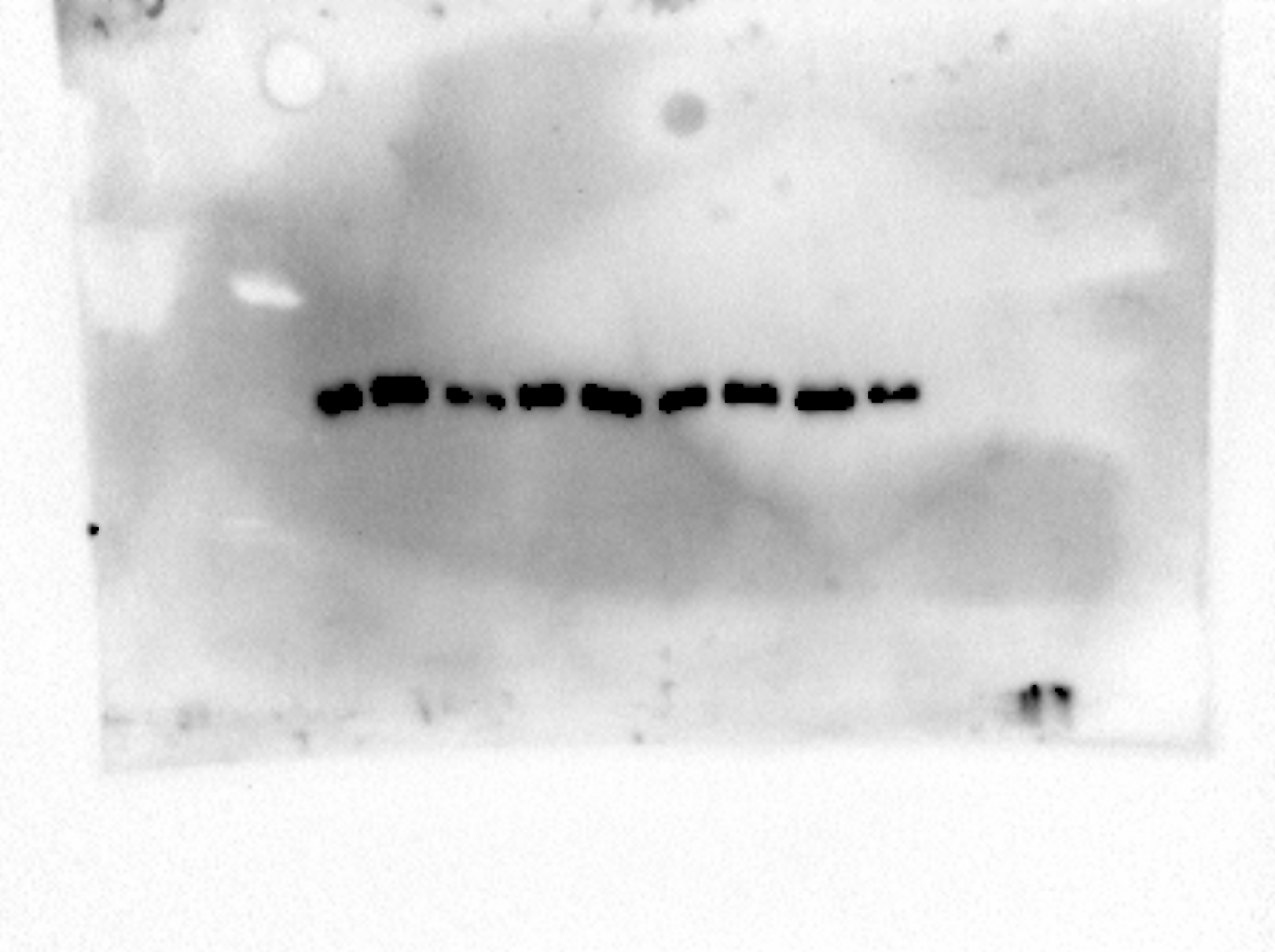

Supplement: Figure 5—figure supplement 1—source data 3. [file elife-104233-fig5-figsupp1-data3.zip › Figure 5 - figure supplement 1/anti-Tubulin (Right panel).tif]

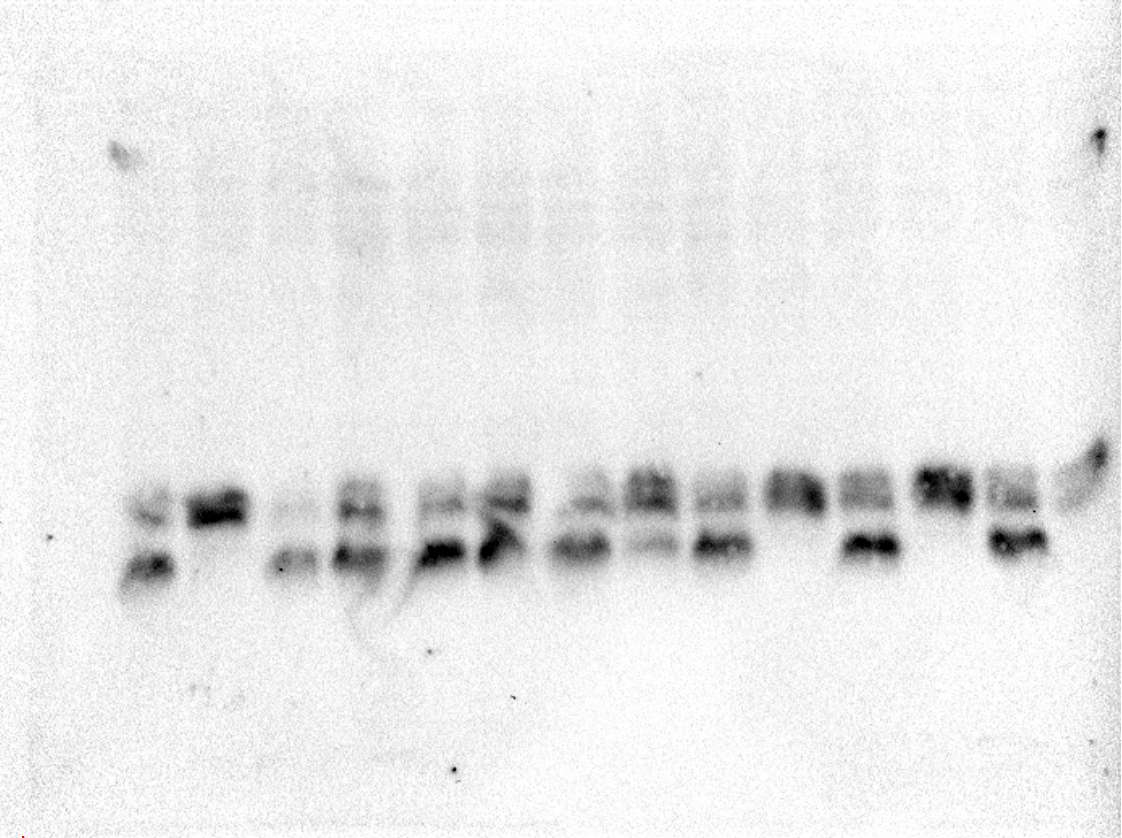

Supplement: Figure 5—figure supplement 1—source data 3. [file elife-104233-fig5-figsupp1-data3.zip › Figure 5 - figure supplement 1/phostag_left1.tif]

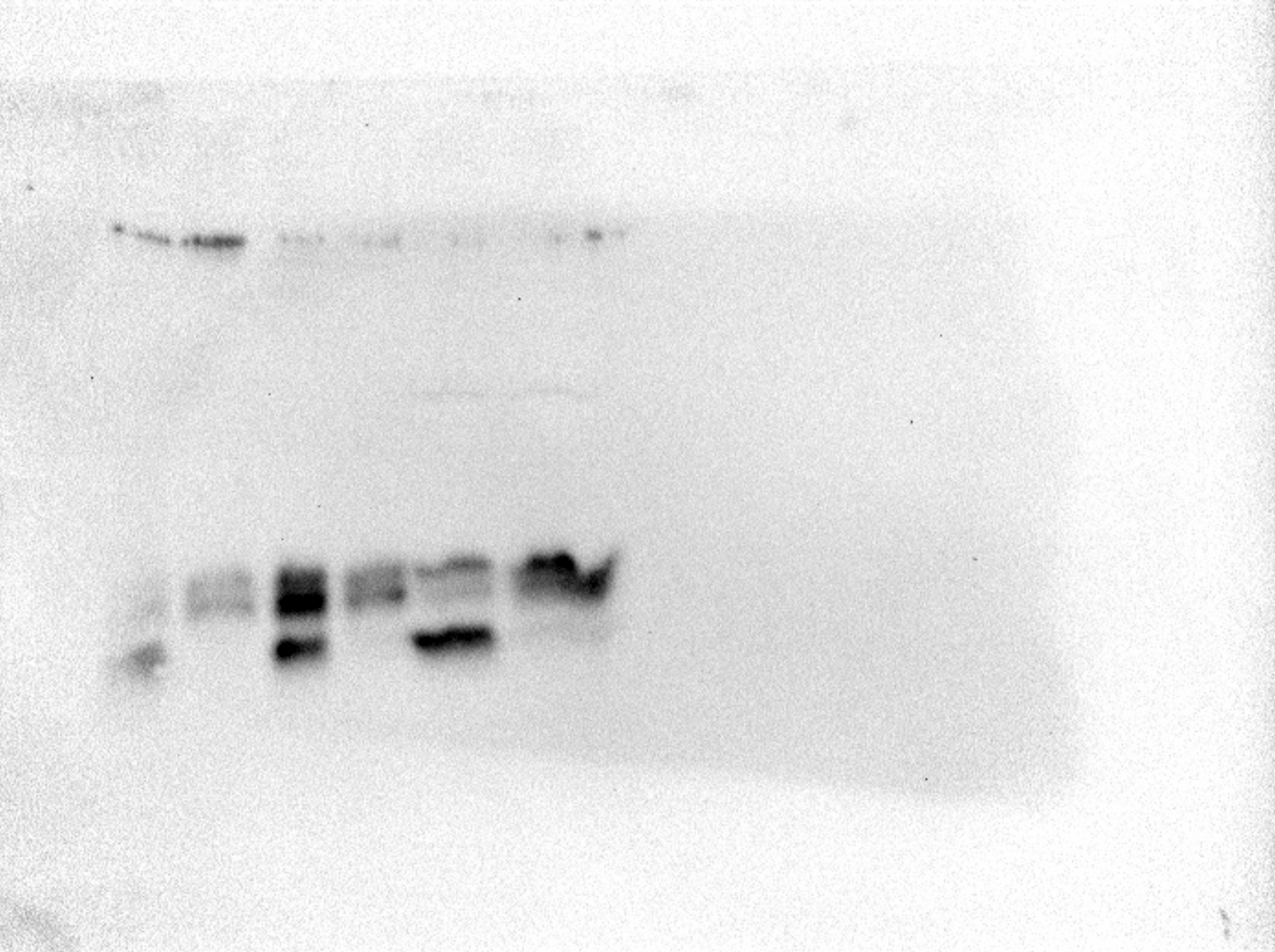

Supplement: Figure 5—figure supplement 1—source data 3. [file elife-104233-fig5-figsupp1-data3.zip › Figure 5 - figure supplement 1/Phostag_left2.tif]

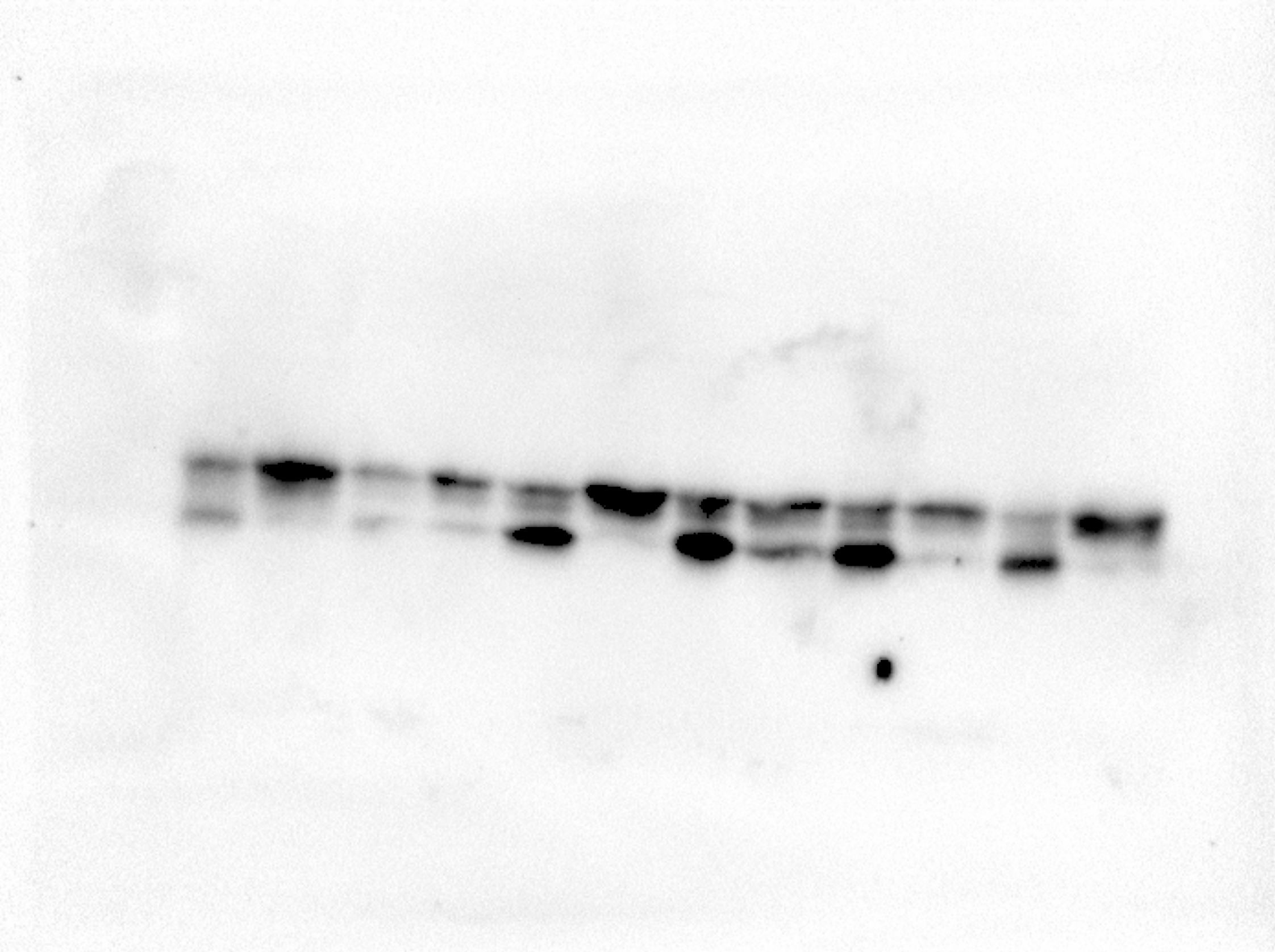

Supplement: Figure 5—figure supplement 1—source data 3. [file elife-104233-fig5-figsupp1-data3.zip › Figure 5 - figure supplement 1/phostag_middle.tif]

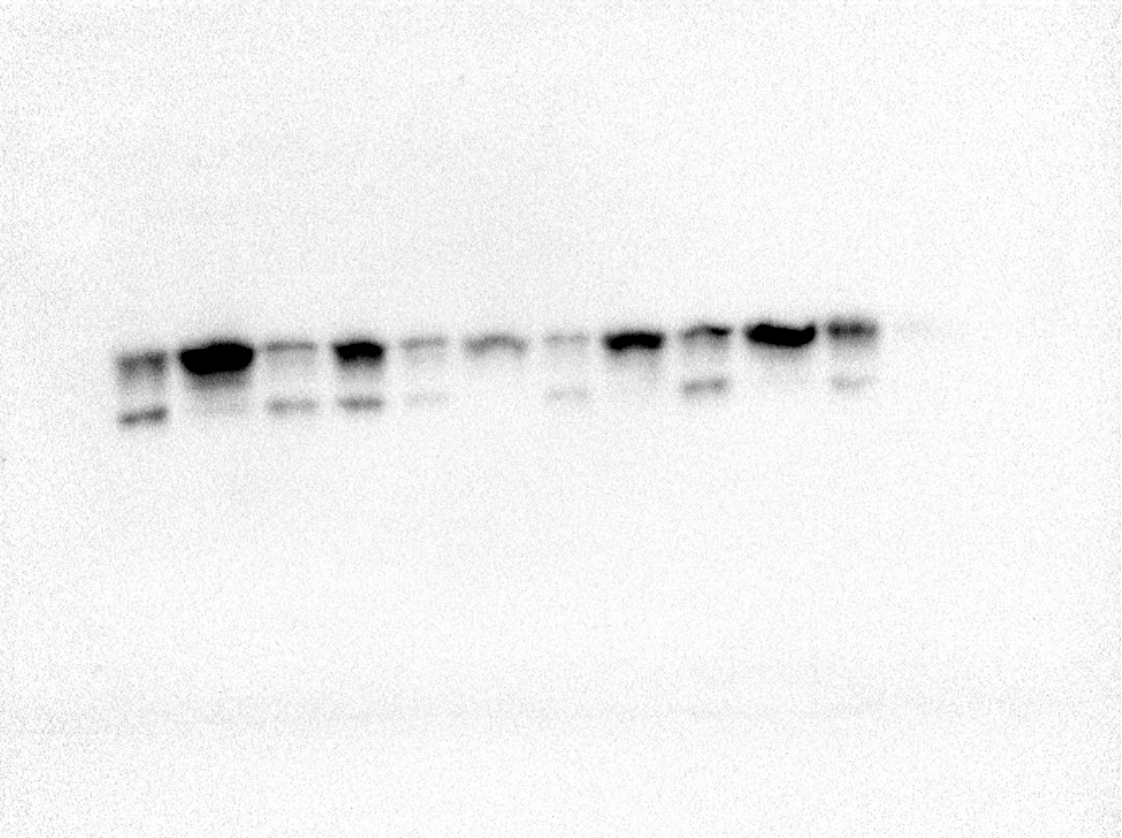

Supplement: Figure 5—figure supplement 1—source data 3. [file elife-104233-fig5-figsupp1-data3.zip › Figure 5 - figure supplement 1/phostag_right1.tif]

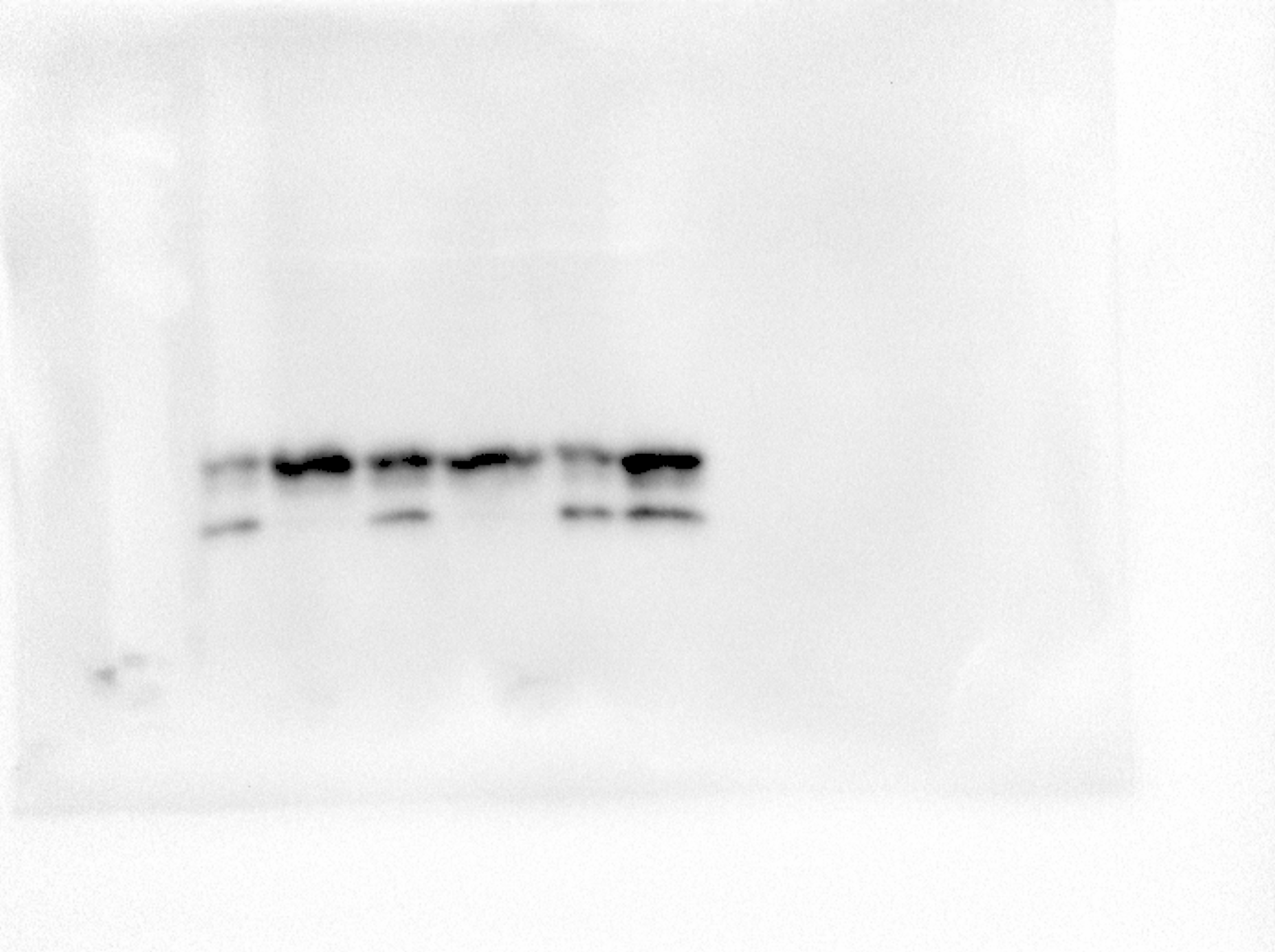

Supplement: Figure 5—figure supplement 1—source data 3. [file elife-104233-fig5-figsupp1-data3.zip › Figure 5 - figure supplement 1/phostag_right2.tif]

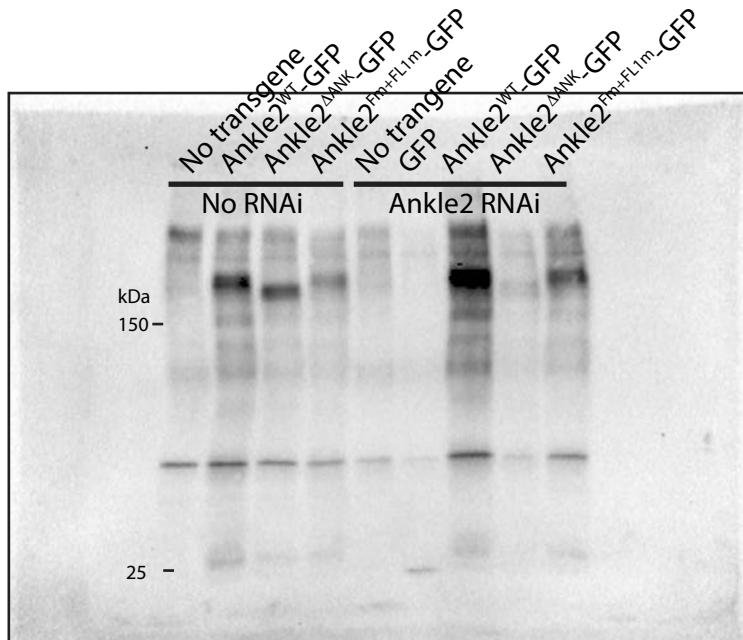

α-GFP

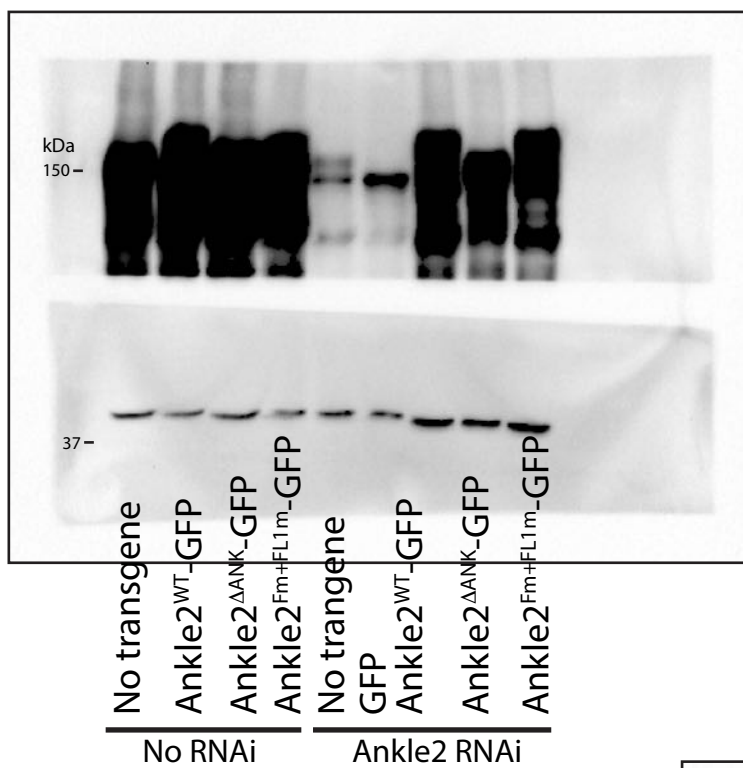

α-Actin

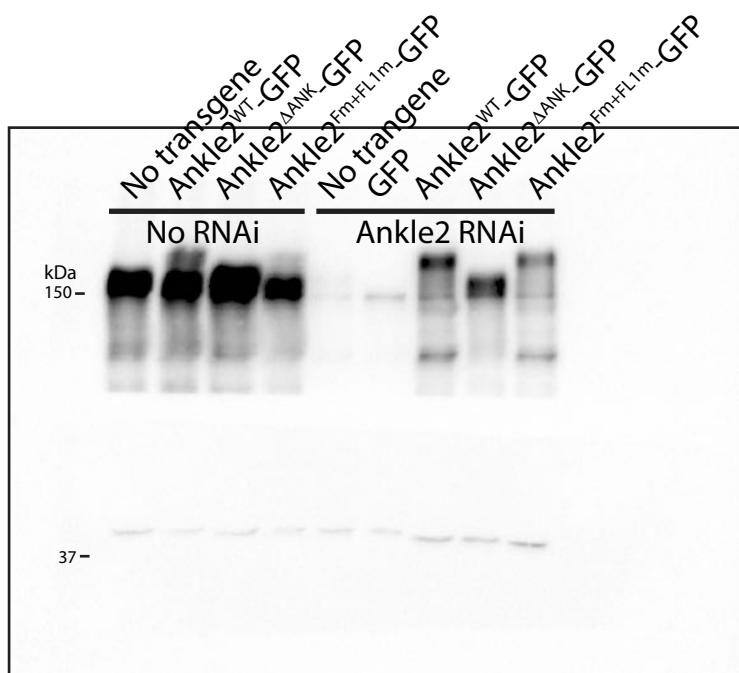

α-Ankle2

Supplement: Figure 6—figure supplement 1—source data 1. [file elife-104233-fig6-figsupp1-data1.zip › Figure 6 - figure supplement 1/Figure 6 - figure supplement 1.pdf]

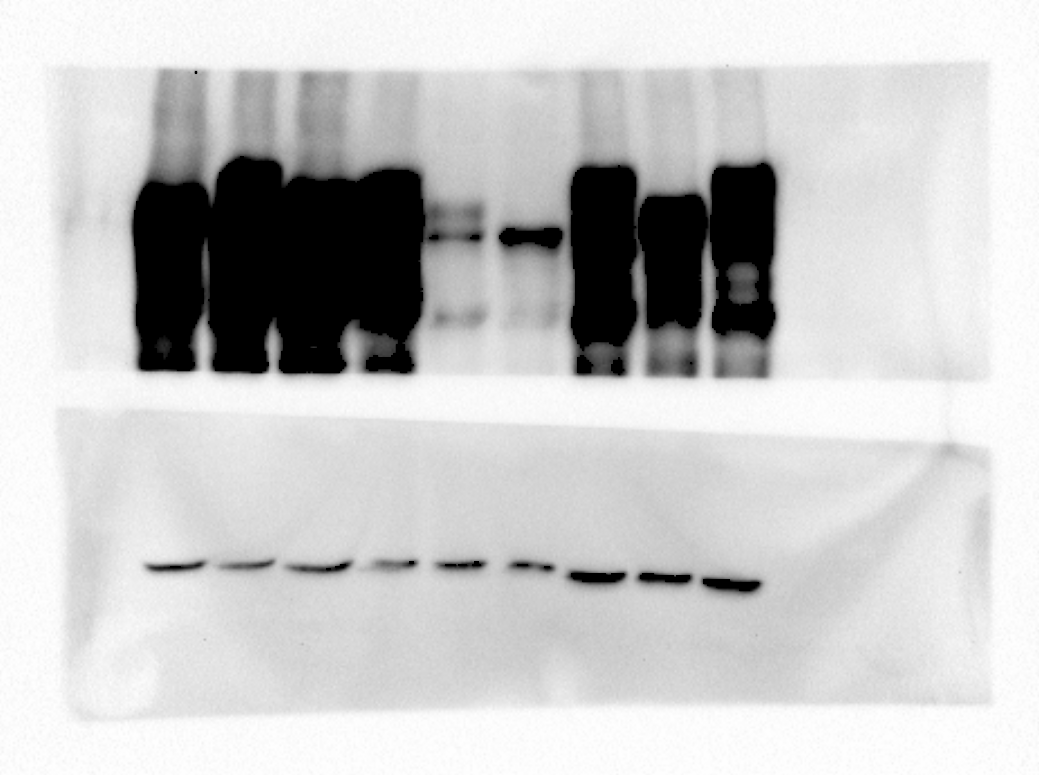

Supplement: Figure 6—figure supplement 1—source data 2. [file elife-104233-fig6-figsupp1-data2.zip › Figure 6 - figure supplement 1/anti-Actin.tif]

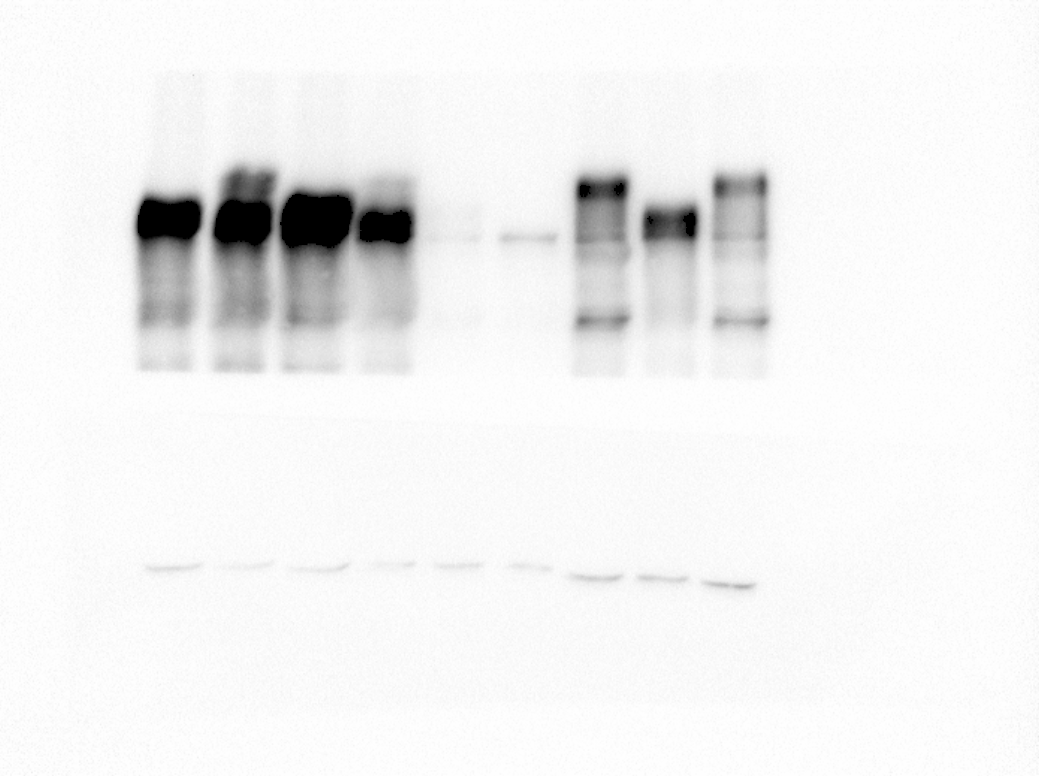

Supplement: Figure 6—figure supplement 1—source data 2. [file elife-104233-fig6-figsupp1-data2.zip › Figure 6 - figure supplement 1/anti-Ankle2.tif]

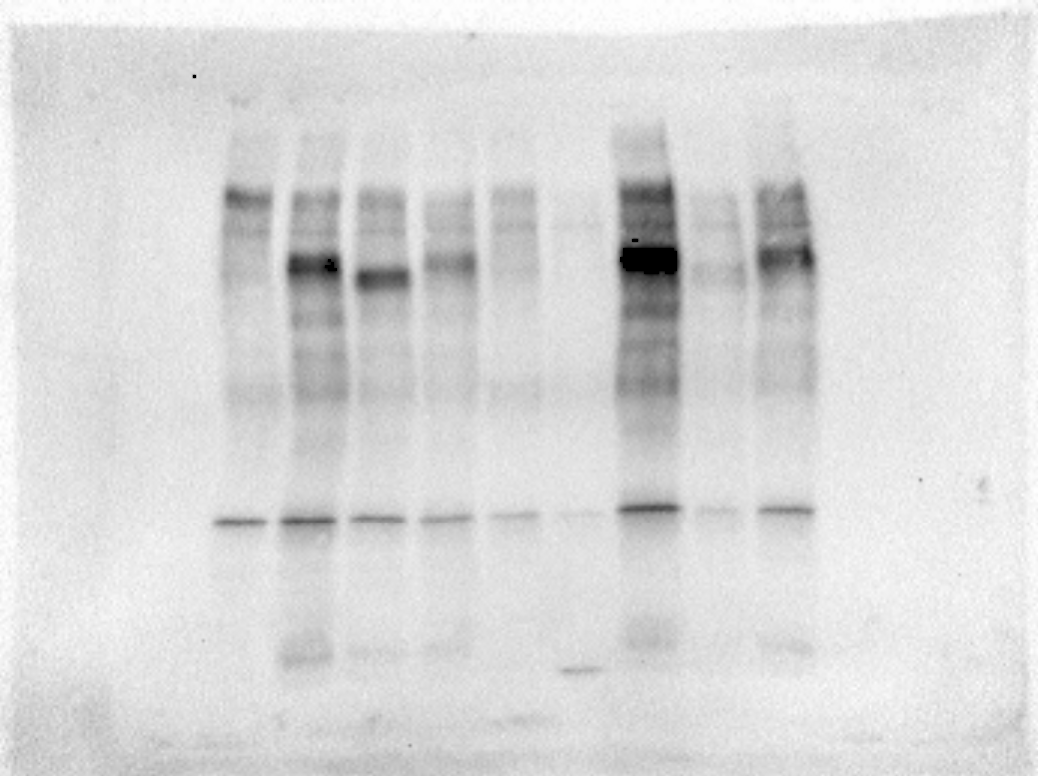

Supplement: Figure 6—figure supplement 1—source data 2. [file elife-104233-fig6-figsupp1-data2.zip › Figure 6 - figure supplement 1/anti-GFP.tif]

Mat $\alpha$ 4>

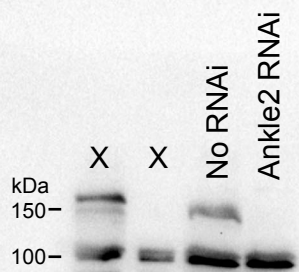

$\alpha$ -Ankle2

Supplement: Figure 7—figure supplement 1—source data 2. [file elife-104233-fig7-figsupp1-data2.zip › Figure 7 - figure supplement 1/Figure 7 - figure supplement 1A.pdf]

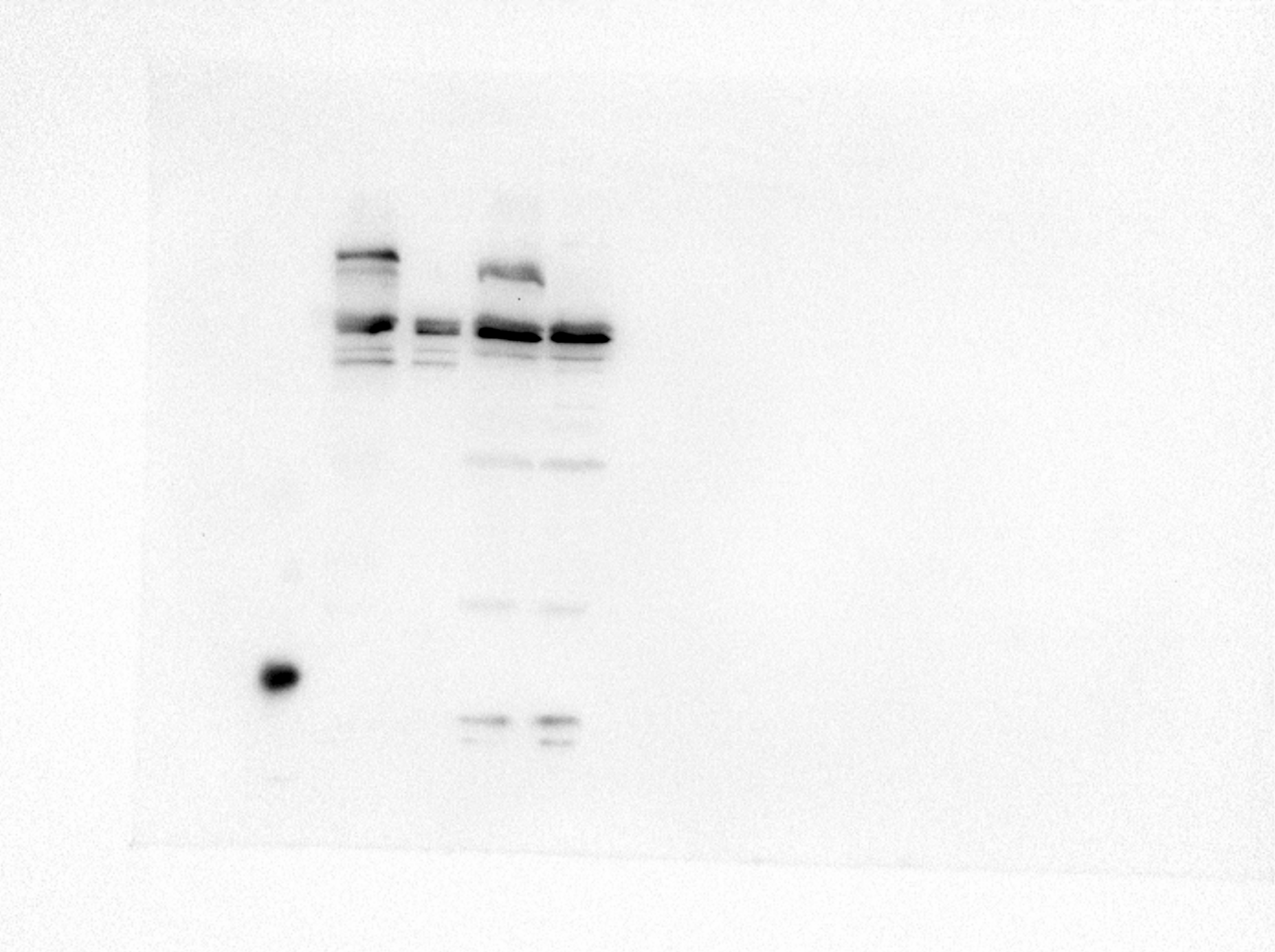

Supplement: Figure 7—figure supplement 1—source data 3. [file elife-104233-fig7-figsupp1-data3.zip › Figure 7 - figure supplement 1/anti-Ankle2.tif]
