# Supplementary material for: Mechanisms of PP2A-Ankle2 dependent nuclear reassembly after mitosis
Source: eLife. 2025 Feb 18;13:RP104233. doi: 10.7554/eLife.104233 (PMC11835388; doi:10.7554/eLife.104233)
Supplement: Figure 4—source data 1. [file elife-104233-fig4-data1.zip › Figure 4/Figure 4B.pdf]

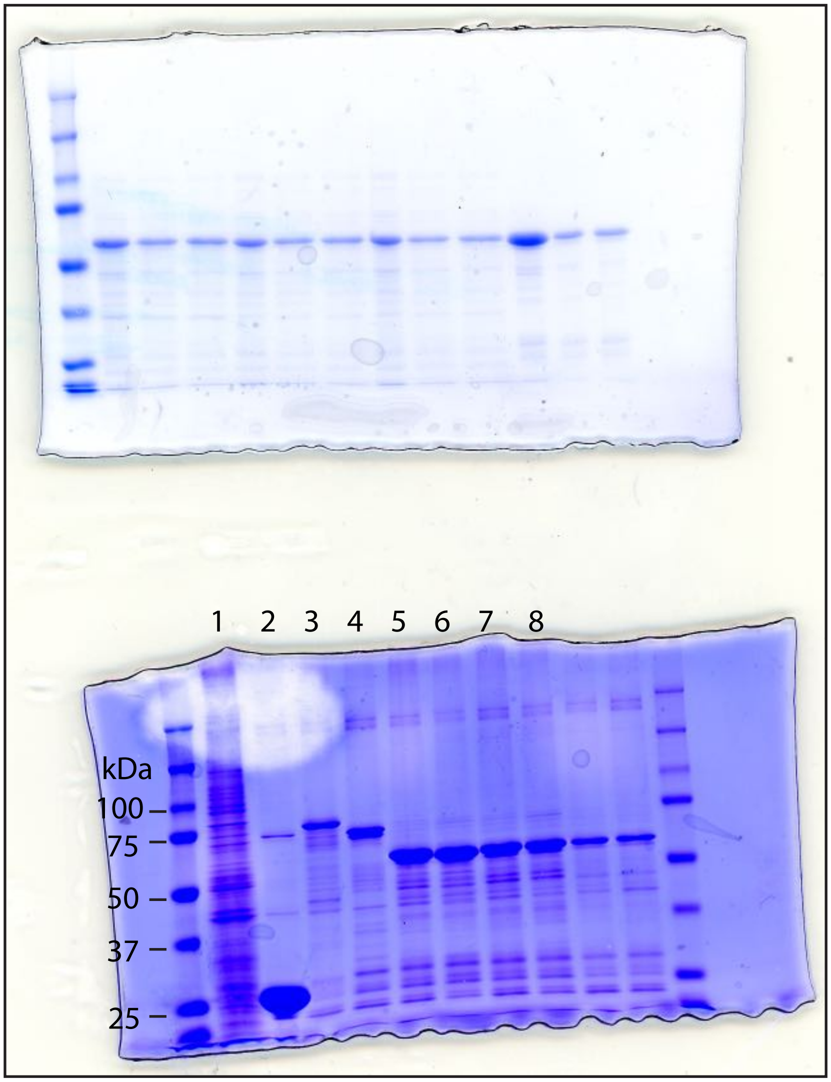

1: WCL
2: GST
3: GST-Ankle2 1-450
4: GST-Ankle2 451-909
5: GST-Ankle2 910-1174
6: GST-Ankle2 910-1174+Fm
7: GST-Ankle2 910-1174+FL2m
8: GST-Ankle2 910-1174+FL1m

CB

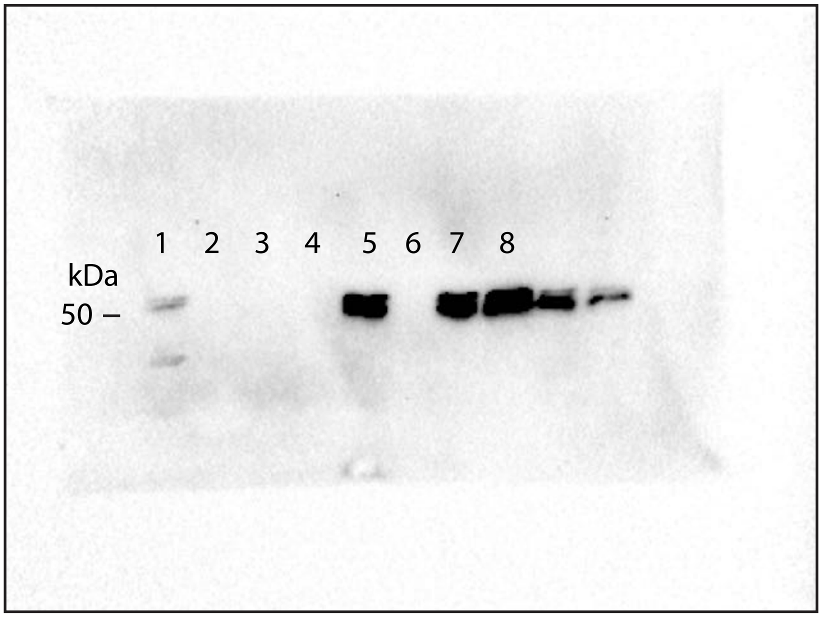

1: WCL
2: GST
3: GST-Ankle2 1-450
4: GST-Ankle2 451-909
5: GST-Ankle2 910-1174
6: GST-Ankle2 910-1174+Fm
7: GST-Ankle2 910-1174+FL2m
8: GST-Ankle2 910-1174+FL1m

α-Myc
