# Supplementary material for: Mechanisms of PP2A-Ankle2 dependent nuclear reassembly after mitosis
Source: eLife. 2025 Feb 18;13:RP104233. doi: 10.7554/eLife.104233 (PMC11835388; doi:10.7554/eLife.104233)
Supplement: Figure 4—source data 1. [file elife-104233-fig4-data1.zip › Figure 4/Figure 4E.pdf]

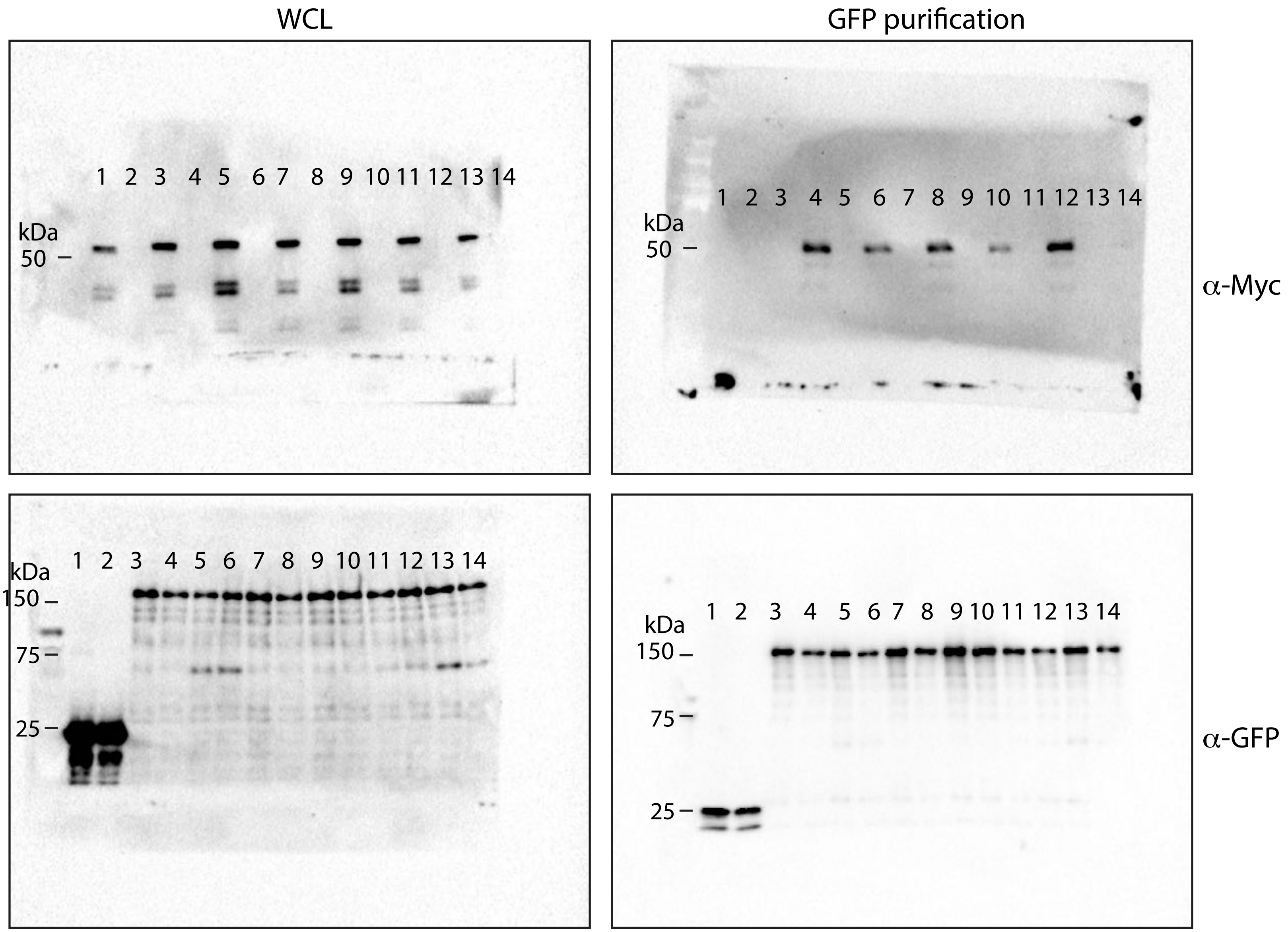

1: GFP-Flag
2: GFP-Flag+ Vap33-myc
3: Ankle2-GFP
4: Ankle2-GFP+Vap33-myc
5: Ankle2^{Fm}-GFP
6: Ankle2^{Fm}-GFP+Vap33-myc
7: Ankle2^{FL2m}-GFP
8: Ankle2^{FL2m}-GFP+Vap33-myc
9: Ankle2^{FL1m}-GFP
10: Ankle2^{FL1m}-GFP+Vap33-myc
11: Ankle2$^{Fm+FL2m}$-GFP
12: Ankle2$^{Fm+FL2m}$-GFP+Vap33-myc
13: Ankle2$^{Fm+FL1m}$-GFP
14:Ankle2$^{Fm+FL1m}$-GFP+Vap33-myc
