# Supplementary material for: Mechanisms of PP2A-Ankle2 dependent nuclear reassembly after mitosis
Source: eLife. 2025 Feb 18;13:RP104233. doi: 10.7554/eLife.104233 (PMC11835388; doi:10.7554/eLife.104233)
Supplement: Figure 5—source data 2. [file elife-104233-fig5-data2.zip › Figure 5/Figure 5D.pdf]

α-Ankle2

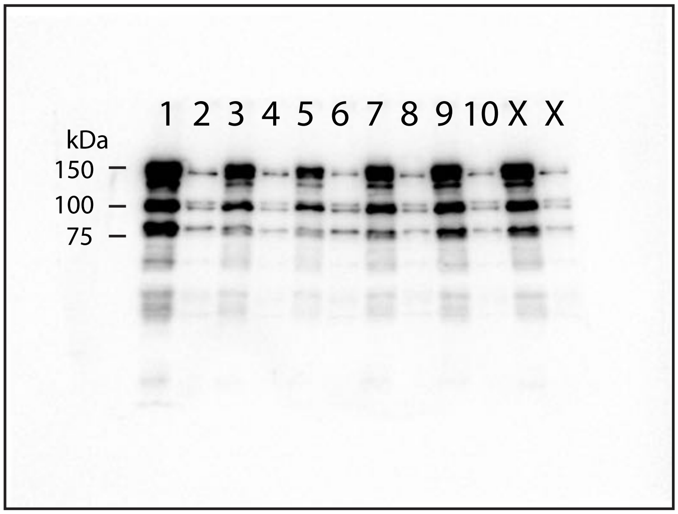

α-Tubulin

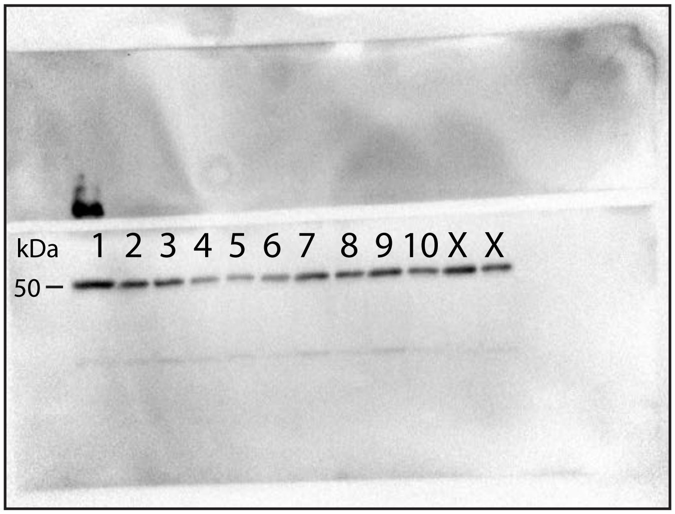

α-GFP

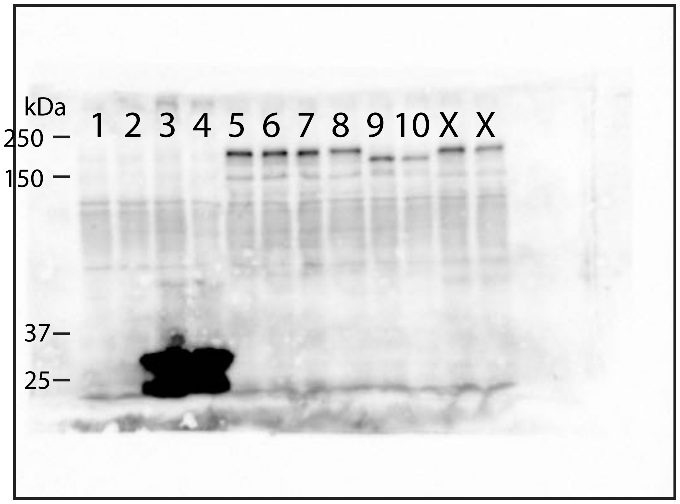

PhosTag+α-BAF

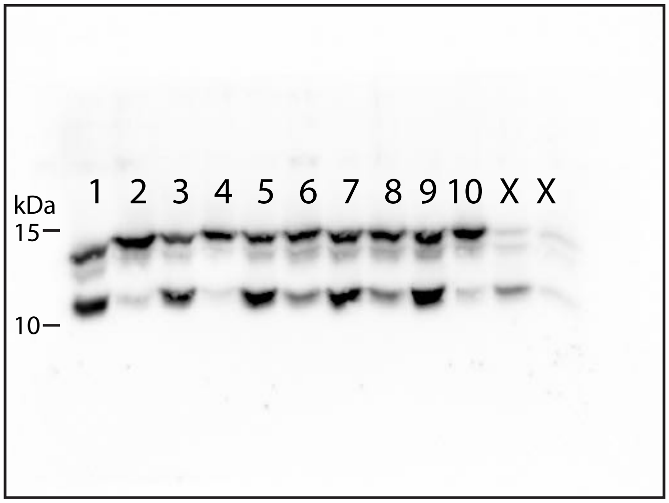

1: dsRNA control<br>2: dsRNA Ankle2	None
3: dsRNA control<br>4: dsRNA Ankle2	Flag-GFP
5: dsRNA control<br>6: dsRNA Ankle2	Ankle2^{WT}-GFP
7: dsRNA control<br>8: dsRNA Ankle2	Ankle2$^{Fm+FL1m}$-GFP
9: dsRNA control<br>10: dsRNA Ankle2	Ankle2$^{\Delta ANK}$-GFP
